# Mechanism of O_2_ Activation and Cysteine Oxidation by the Unusual Mononuclear Cu(I) Active Site of the Formylglycine-Generating Enzyme

**DOI:** 10.1021/acscentsci.5c00183

**Published:** 2025-04-04

**Authors:** Ioannis Kipouros, Hyeongtaek Lim, Mason J. Appel, Katlyn K. Meier, Britt Hedman, Keith O. Hodgson, Carolyn R. Bertozzi, Edward I. Solomon

**Affiliations:** † Department of Chemistry, 6429 Stanford University, Stanford, California 94305, United States,; ‡ Department of Molecular and Cell Biology, University of California, Berkeley, California 94720, United States,; § Stanford Synchrotron Radiation Lightsource, SLAC National Accelerator Laboratory, Stanford University, Menlo Park, California 94025, United States_,_; ∥ Sarafan ChEM-H and Howard Hughes Medical Institute, Stanford University, Stanford, California 94305, United States

## Abstract

The formylglycine-generating enzyme (FGE) catalyzes the selective oxidation of a peptidyl-cysteine to form formylglycine, a critical cotranslational modification for type I sulfatase activation and a useful bioconjugation handle. Previous studies have shown that the substrate peptidyl-cysteine binds to the linear bis-thiolate Cu­(I) site of FGE to form a trigonal planar tris-thiolate Cu­(I) structure that activates O_2_ for the oxidation of the C_β_–H of the cysteine substrate via an unknown mechanism. Here, we employed a combination of stopped-flow kinetic, spectroscopic (UV–vis absorption, XAS, and EPR), and computational (DFT/TD-DFT calculations) methods to observe and characterize the key intermediates in this reaction for FGE from *Streptomyces coelicolor*_._ Our results define the reaction coordinate of FGE, which involves H-atom abstraction from the C_β_–H bond of the cysteine substrate by a reactive Cu­(II)–O_2_^•–^ species to form the now experimentally observed Cu­(I)–OOH intermediate bound to a peptidyl-thioaldehyde, which proceeds to oxidize one of the protein-derived cysteine residues to a sulfenate that is end-on O-coordinated to Cu­(I). These results provide fundamental insights into how the unusual mononuclear Cu­(I) site of FGE activates O_2_ for cysteine oxidation and stores oxidizing equivalents during catalysis by employing a Cu­(I)–sulfenate intermediate with an end-on O-coordination that is unprecedented in biology.

## Graphical abstract

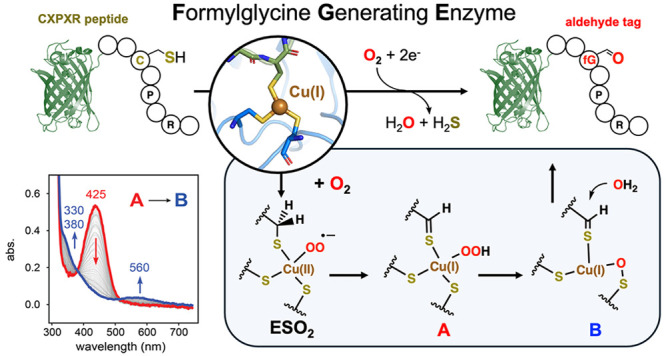

## 1 Introduction

The formylglycine-generating enzyme (FGE) uses O_2_ for the selective conversion of the peptidyl-cysteine residue in a CXPXR minimum consensus protein sequence into the electrophilic formylglycine (fGly) group (Figure 1A).^1^ In its biological context, FGE is required for the co- or post-translational activation of type I sulfatases, wherein fGly is hydrated to form the vicinial diol that functions as a nucleophile in the covalent catalysis of organosulfate hydrolysis (Figure 1A, gray box).^1^ FGE has been employed as a biocatalyst for bioconjugation applications outside its native context for close to two decades^2^ but suffered from poor catalytic efficiency and low yields in specific contexts until the recent discovery that Cu­(I) is a required cofactor for its reactivity.^3,4^ This discovery generated significant interest in elucidating the reaction mechanism of FGE both to enhance its native reactivity for biotechnology applications and to explore broader fundamental questions in Cu/O_2_ bioinorganic chemistry.

**Figure 1:**
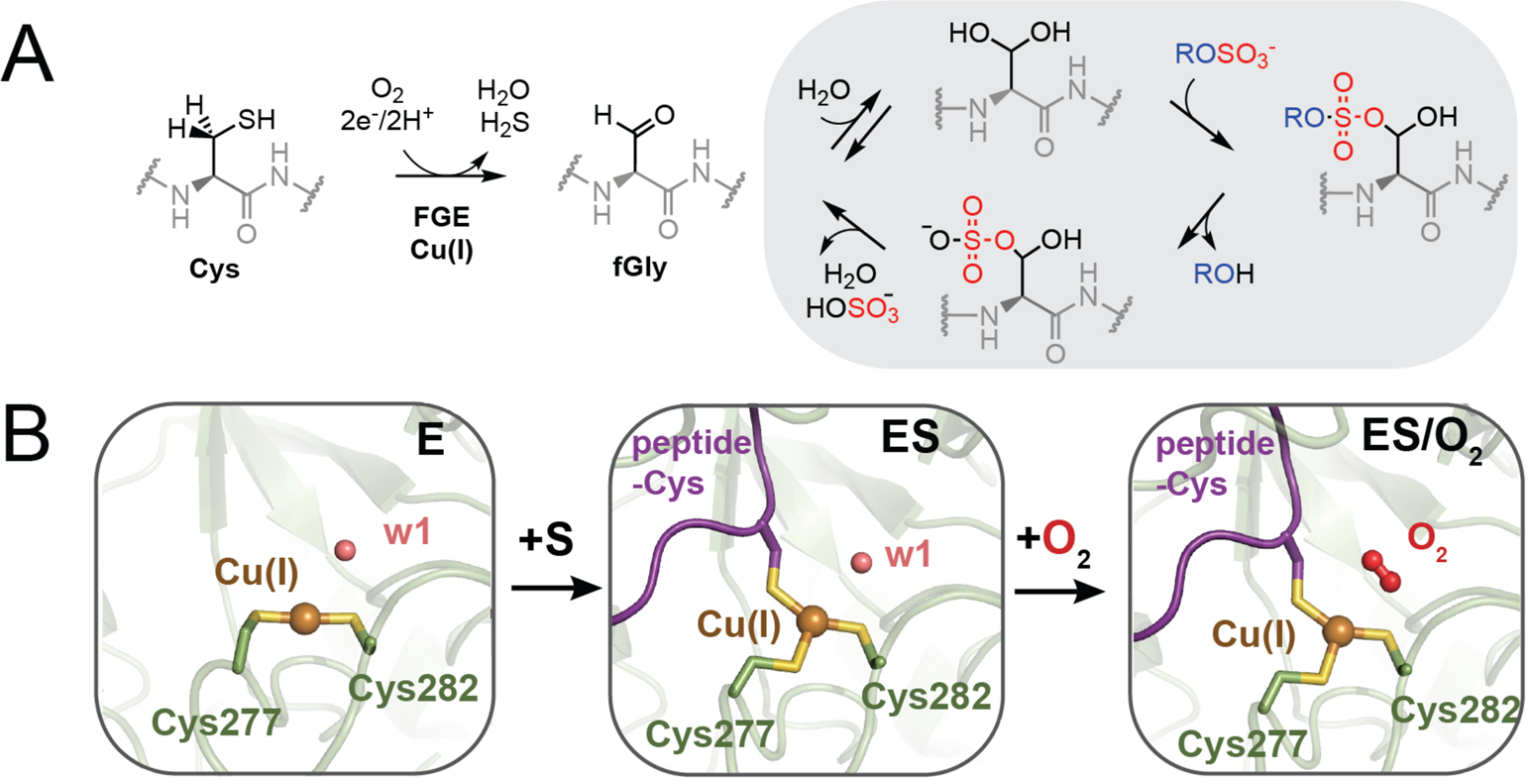
Peptidyl cysteine oxidation to fGly by FGE. (A) In its native biological context, FGE catalyzes the selective oxidation of a cysteine residue to fGly, a post-translational modification required for the catalytic function of type I sulfatases (in the gray box). (B) Crystallographic structures of early steps in the FGE mechanism including its Cu­(I)-bound state (E, PDB: 6MUJ, *S. coelicolor*),^14^ its substrate-bound complex (ES, PDB: 6S07, *T. curvata*),^19^ and its noncoordinating O_2_-bound state (ES/O_2_, PDB: 6XTQ, *T. curvata)*.^15^ The FGE protein is shown in green (residue numbers for FGE from *S. coelicolor* are used), the substrate peptide is shown in purple, the Cu­(I) site is shown as a brown sphere, the crystallographic water (w1) is shown as a pink sphere, and the bound O_2_ cosubstrate is shown in red.

Most O_2_-activating metalloenzymes containing Cu­(I) sites, such as the coupled binuclear copper enzymes, the multicopper oxidases, and the heme-copper oxidases, employ multiple metal sites that provide the required electrons for controlled O_2_ reduction.^5^ A few metalloenzymes, including galactose oxidase (GaOx) and amine oxidase, employ mononuclear Cu sites; however, these also contain redox-active covalently bound cofactors, which together couple substrate oxidation to the 2e-reduction of O_2_ to H_2_O_2_.^5–7^ Metalloenzymes that activate O_2_ at mononuclear Cu­(I) active sites without employing additional redox-active metals or organic cofactors are exceedingly rare in biology, with only three proposed cases: (i) the lytic polysaccharide monooxygenases (LPMOs), which appear to preferably utilize H_2_O_2_ over O_2_ as the cosubstrate oxidant;^8–10^ (ii) the particulate methane monooxygenases (pMMOs), for which binuclear, and trinuclear Cu­(I) sites have also been invoked in addition to mononuclear Cu­(I) sites;^11,12^ and (iii) FGE, which remains the only oxidase enzyme with a bona fide mononuclear Cu­(I) site that utilizes O_2_ as its native oxidant.^13,14^ Thus, in addition to its utility in biotechnological and therapeutic applications, FGE offers an O_2_-activating mononuclear Cu­(I) site to explore a novel metalloenzyme mechanism.

Under oxidizing conditions, the two active-site cysteines of apo-FGE form a disulfide bond.^3^ Reduction of this disulfide bond allows FGE to bind Cu­(I) with high affinity (*K*_d_ ∼ 10^–17^ M) via a linear bis-thiolate ligation (E, Figure 1B).^13^ The CXPXR-containing substrate binds first to E via coordination of its peptidyl-Cys to form a trigonal tris-thiolate Cu­(I) site (ES, Figure 1B),^14^ followed by the binding of O_2_ to a proximal protein pocket site via displacement of a structured water (w1 in Figure 1B) and without metal coordination (ES/O_2_, Figure 1B).^15^ The unique trithiolate ligand set in the Cu­(I) site of FGE is reminiscent of the well-characterized metallochaperone proteins involved in copper homeostasis (e.g., Atox1, Hah1),^16^ but unprecedented in metalloxidases or metallooxygenases, suggesting that FGE employs a novel mechanism for O_2_ activation. Steady-state kinetic studies using a deuterated β-cysteine substrate revealed a normal primary C–H/D kinetic isotope effect (KIE) on *k*_cat_, indicating that the rate-limiting step involves H atom abstraction (HAA) from the substrate C–H bond by a reactive Cu/O_2_ intermediate.^17^ However, this reactive species and the subsequent post-HAA intermediates had not been observed experimentally, and thus their geometric and electronic structures had remained a subject of debate and only explored computationally.^18^ Thus, the O_2_ activation and peptidyl-cysteine oxidation mechanism of FGE, as well as its structure–function correlations to other O_2_-activating metal sites in biology, remain open questions.

In this study, we employed a combination of kinetic, spectroscopic, and computational methods to observe and characterize a series of key intermediates in the catalytic cycle of FGE from *Streptomyces coelicolor* and defined their geometric and electronic structures. Our results provide molecular-level insights into how the tris-thiolate Cu­(I) site of the ES complex enables O_2_ binding and activation to generate the proposed Cu­(II)–O_2_^•–^ species that performs the HAA step from the substrate C–H bond. This leads to the now spectroscopically observed post-HAA intermediate containing the thioaldehyde product coordinated to a Cu­(I)–OOH site, which proceeds to oxygenate one of the protein-derived cysteinate ligands to sulfenate. The resulting Cu­(I)–sulfenate bond in FGE is defined by X-ray absorption spectroscopy (XAS) to be a new type of end-on sulfenate­(O)–Cu­(I) coordination in biology. Overall, within the context of previous biochemical, crystallographic, and spectroscopic work,^3,13–15,17,18^ this study reveals the complete mechanism of FGE, including the O_2_ activation and cysteine oxidation steps by its unusual mononuclear Cu­(I) site, and further delineates the multiple redox functions of its active site cysteine ligands, ranging from the control of metal binding to storage of oxidizing equivalents during catalysis.

## 2 Results and Analysis

### 2.1 Single-Turnover Kinetics for the Reaction of the FGE/Cu­(I)/Substrate Complex with O_2_

To obtain insights into the O_2_ activation and peptidyl-cysteine oxidation reactions of FGE, the single-turnover reaction of the FGE/Cu­(I)/substrate complex (ES; substrate is the 14mer-peptide with the CXPXR recognition motif shown in Scheme S1) with O_2_ was investigated by stopped-flow absorption (SF-Abs) kinetics. Upon 1:1 (v/v) stopped-flow mixing of an anaerobic solution of the ES complex (0.1 mM, postmixing; FGE from *S. coelicolor*) with an O_2_-saturated buffer solution (∼1.1 mM, postmixing), at pH 9.0 and 4 °C, the time-dependent UV–vis absorption spectra of this reaction showed the sequential formation and decay of three chromophoric species. First, at early reaction times (0–0.6 s), an intense absorption feature at 425 nm develops (Figure 2A), which corresponds to the first observed reaction intermediate (intermediate A). We previously reported the observation of this chromophoric species in FGE,^14^ but in the absence of additional spectroscopic and kinetic data its assignment remained elusive. This 425 nm feature decays with the concomitant formation of three new absorption features at 330, 380, and 560 nm (Figure 2B). The presence of two isosbestic points (marked by asterisks in Figure 2B) indicates the direct conversion of intermediate A to a new species (intermediate B). Notably, rapid chemical quench in tandem with product quantification by high-performance liquid chromatography (HPLC) reveals that the formation of intermediate B is coupled to product formation (either as the final fGly product or as its possible thioaldehyde precursor that would immediately hydrolyze to fGly under acidic chemical quenching conditions; Figure S1). Finally, the 560 nm feature decays slowly along with an increase in the intensity of the high-energy bands at 330–380 nm (Figure 2C). The presence of another isosbestic point (marked by the asterisk in Figure 2C) indicates that intermediate B is directly converted to another intermediate (intermediate C). Intermediate C is stable and appears to be the final species in the single-turnover FGE reaction. No reaction is observed upon the further addition of O_2_ to a solution containing intermediate C that has been equilibrated with additional substrate equivalents. However, upon treatment with the 2e^–^ reductant, dithiothreitol (DTT), and subsequent equilibration with the substrate, intermediate C reacts anew with O_2_ to generate the same reaction intermediates observed in the initial reaction of the ES complex with O_2_ (Figure S2).

**Figure 2:**
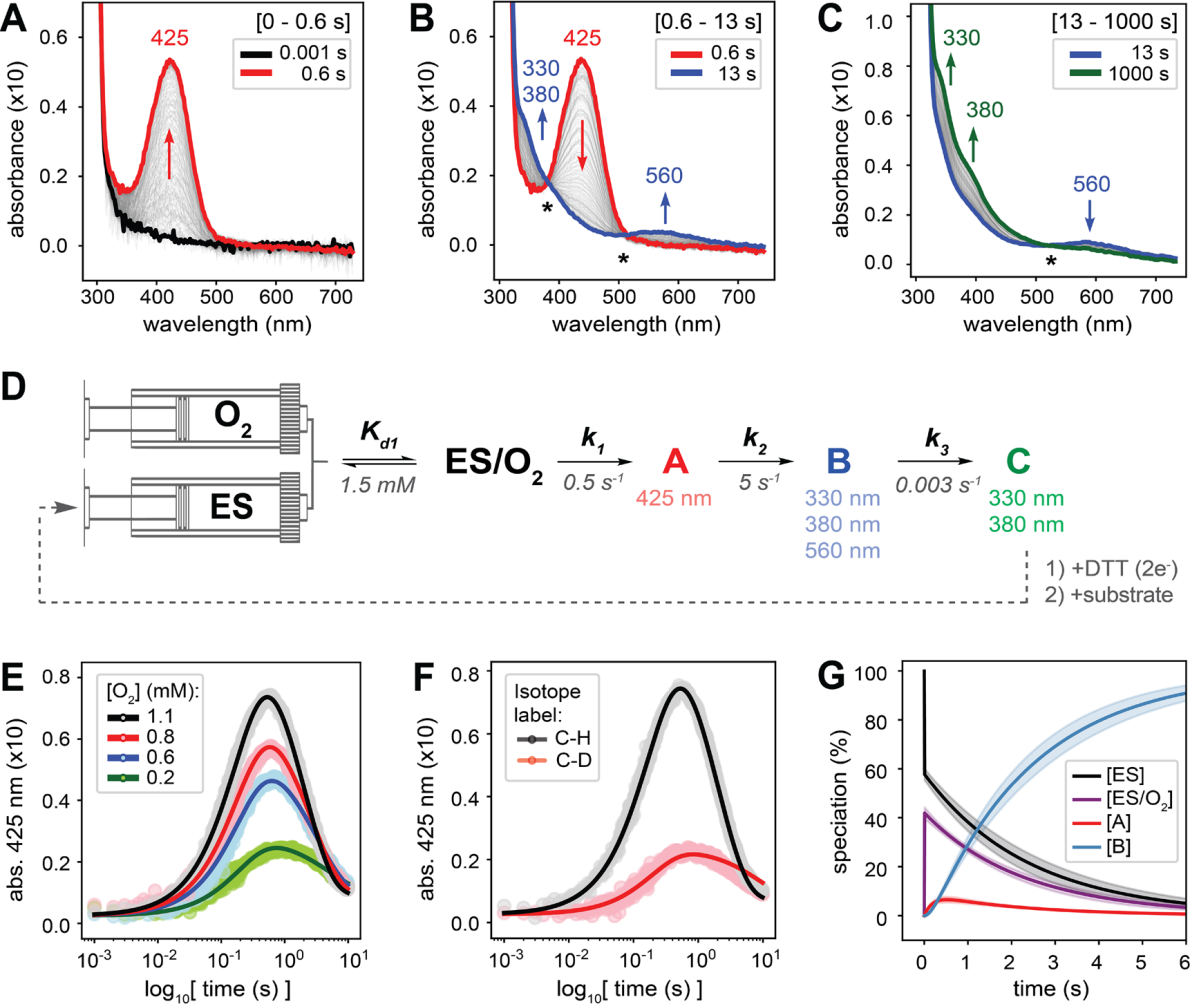
Stopped-flow absorption kinetics for the single-turnover reaction of the FGE/Cu­(I)/substrate (ES) complex with O_**2**_. (A–C) Time-dependent absorption spectra for the reaction of ES (0.1 mM, postmix; FGE from *S. coelicolor*) with O_2_ (∼1.1 mM, postmix) in 50 mM Tris buffer (0.5 M NaCl, pH 9.0, 4 °C) show the sequential formation and decay of chromophoric species (isosbestic points indicated with asterisks): (A) 0–0.6 s, (B) 0.6–13 s, and (C) 13–1000 s. (D) The kinetic scheme for the stopped-flow single-turnover FGE reaction (based on observations from panels A–C) used for the kinetic fitting. After reduction with DTT and subsequent addition of substrate (dashed gray line), the final intermediate C reacts again with O_2_ to generate the same chromophoric intermediates (Figure S2). The reported rate constants are obtained from the SF-Abs experiments shown in panels A–C. (E and F) Dependence of the 425 nm absorbance trace on (E) the O_2_ concentration and (F) substrate C–H/D isotopic labeling (for *K*_d1_ = 1.5 mM). Data points are shown as circles, and kinetic fits are shown as solid lines (for kinetic fitting details for these plots, see SI Methods 1.7). (G) Early time-course speciation of enzyme intermediates (as % of total enzyme concentration) using the fitted kinetic parameters for the reaction of ES (0.1 mM) with O_2_ (1.1 mM) reveals that intermediate A accumulates at ∼6% (at 0.6 s) while intermediate B accumulates at ∼100% (6–20 s). The shaded regions indicate the standard deviations for each speciation curve.

Within the context of previous crystallographic and steady-state kinetics work,^14,15,17^ the above SF-Abs results from this study define a general kinetic scheme for the single-turnover reaction of FGE that is shown in Figure 2D. This kinetic scheme initiates with the mixing of ES and O_2_ to form the ES/O_2_ intermediate via the fast and reversible binding (*K*_d1_) of O_2_ to the FGE protein pocket. The ES/O_2_ intermediate would correspond to the intermediate observed crystallographically by Seebeck and co-workers, where O_2_ binds to the FGE protein pocket without coordination to the Cu­(I) (ES/O_2_ in Figure 1B)^15^ and is distinct from the putative reactive Cu­(II)–O_2_^•–^ species (ESO_2_). Neither ES/O_2_ nor ESO_2_ is assigned as the experimentally observed intermediate A (Figure 2A); instead, they are assigned as earlier intermediates based on two observations. First, a primary substrate C–H/D KIE is observed on the formation of intermediate A (presented below), and second, the distinct UV–vis spectrum of intermediate A, which exhibits a single absorption feature at 425 nm (Figure 2A), is significantly different from the UV–vis absorption spectrum expected for the ESO_2_ species, which should exhibit multiple absorption features corresponding to the thiolate → Cu­(II) and O_2_^•–^ → Cu­(II) charge transfer (CT) transitions (vide infra).^20–23^ The dependence of the 425 nm absorbance trace on the initial O_2_ concentration (Figure 2E) provides additional support for the above assignment. Indeed, fitting our [O_2_]-dependent SF-Abs data under a kinetic model where intermediate A corresponds to ESO_2_, formed via the binding of O_2_ to ES, requires uncharacteristically slow O_2_ binding and dissociation rates (*k*_on_ = 1.5 mM^–1^s^–1^, *k*_off_ = 2.6 s^–1^; Figure S3).^24^ On the contrary, the kinetic model in Figure 2D, where intermediate A forms via an irreversible step (*k*_1_) from ESO_2_, allows for fast and reversible O_2_ binding to ES, consistent with the expected kinetic behavior for small molecule binding to the protein pocket.^24^ Importantly, the kinetic fitting in Figure 2E also sets a lower limit for O_2_ dissociation (*K*_d1_ > ∼1 mM, Figure S4) from ES/O_2_. Together with the reported crystallographic conditions for the generation of ES/O_2_ (Figure 1B), which set a high limit for O_2_ dissociation (*K*_d1_ < ∼2 mM),^15^ our results establish 1–2 mM as the range for *K*_d1_.

After establishing the general kinetic scheme for the single-turnover reaction for FGE (Figure 2D), an isotopically labeled C–D substrate analogue (i.e., 3,3-*d*_2_-cysteine of the 14mer-peptide, Scheme S1) was used to measure KIEs in the single-turnover reaction of ES with O_2_. The early time SF-Abs data show significant differences in the 425 nm absorbance traces between the two substrate isotopes (C–H versus C–D; Figure 2F). Fitting these 425 nm traces using the kinetic model in Figure 2D reveals a primary KIE on the formation of intermediate A (*k*_(C–H)_/*k*_(C–D)_ = 2.0–3.6, depending on the specific *K*_d1_ value within the established 1–2 mM range discussed above and in Figure S4) but not on its conversion to intermediate B (see extended analysis in Figure S5). These results indicate that intermediate A forms via the homolytic cleavage of the substrate C­(sp^3^)–H bond and thus the step defined by *k*_1_ is associated with the HAA from the substrate by the putative reactive ESO_2_ intermediate. This experimentally supports proposals in the noncoupled binuclear enzymes (i.e., Peptidylglycine α-amidating monooxygenase, PHM; Dopamine β-monooxygenase, DβM; Tyramine β-monooxygenase, TβM) which also involve a similar Cu­(II)–O_2_^•–^ intermediate for HAA.^25,26^ It should be noted that Figure 2D describes the simplest kinetic model required to fit the SF-Abs data, where the HAA step is associated with *k*_1_. However, this step can be expanded into two elementary steps consisting of the fast and reversible formation of the endergonic ESO_2_ (via O_2_ coordination to ES/O_2_ described by K_d1_′ in Scheme S2) and the subsequent slow and irreversible HAA step (*k*_1_′ in Scheme S2). While *K*_d1_′ cannot be probed experimentally in our SF-Abs kinetics, under the fast *K*_d1_′ regime, *k*_1_ *′* would share the same value as *k*_1_. Thus, the kinetic scheme in Figure 2D is sufficient to describe HAA and the subsequent steps involving intermediates A–C.

To evaluate whether intermediates A–C can be cryogenically trapped at accumulations suitable for further spectroscopic characterization, the above kinetic model and associated kinetic parameters were employed to simulate the early- and late-time speciation for the reaction of ES with O_2_ (Figure 2G and Figure S6, respectively). These results indicate that intermediate A exhibits a maximum accumulation of ∼6% of the total enzyme concentration (at 0.6 s). This low accumulation of intermediate A prevents its further characterization by many bioinorganic spectroscopic techniques, such as electron paramagnetic resonance (EPR) and X-ray absorption spectroscopy (XAS). However, the primary KIE on the formation of intermediate A (*k*_1_) and its UV–vis absorption spectrum obtained from SF-Abs experiments provide valuable experimental results that can be correlated to electronic structure calculations that allow its definition (in section 2.3). Both intermediates B and C accumulate at ∼100% at 15 s and 15 min, respectively (Figure 2G and Figure S6). Therefore, in addition to obtaining their UV–vis absorption spectra from the SF-Abs experiments, intermediates B and C are suitable for hand-quenched freeze-trapping and detailed spectroscopic characterization (section 2.2).

### 2.2 Spectroscopic Definition of Intermediates

The electronic absorption spectra for intermediates A–C were extracted from our SF-Abs kinetic data (Figure 3A-C) using the early- and late-time speciation plots (Figure 2G and Figure S6B, respectively). Subsequent band-shape analysis with Gaussian fits provided quantitative spectral information about the associated UV–vis transitions of these intermediates (Table S1). Intermediate A exhibits a single, intense absorption band at 423 nm (ε_423 nm_ = 8360 M^–1^ cm^–1^; Figure 3A). Intermediate B exhibits two high-energy bands at 323 and 363 nm and a very weak low-energy band at 544 nm (ε_544 nm_ = 35 M^–1^ cm^–1^; Figure 3B). Intermediate C exhibits two high-energy bands at 328 and 366 nm (Figure 3C). The different energies and intensities of these UV–vis absorption bands reflect the distinct electronic and geometric structures of each intermediate and are correlated to TD-DFT calculations for a series of possible intermediates proposed to be involved in the FGE mechanism (in section 2.3).

**Figure 3:**
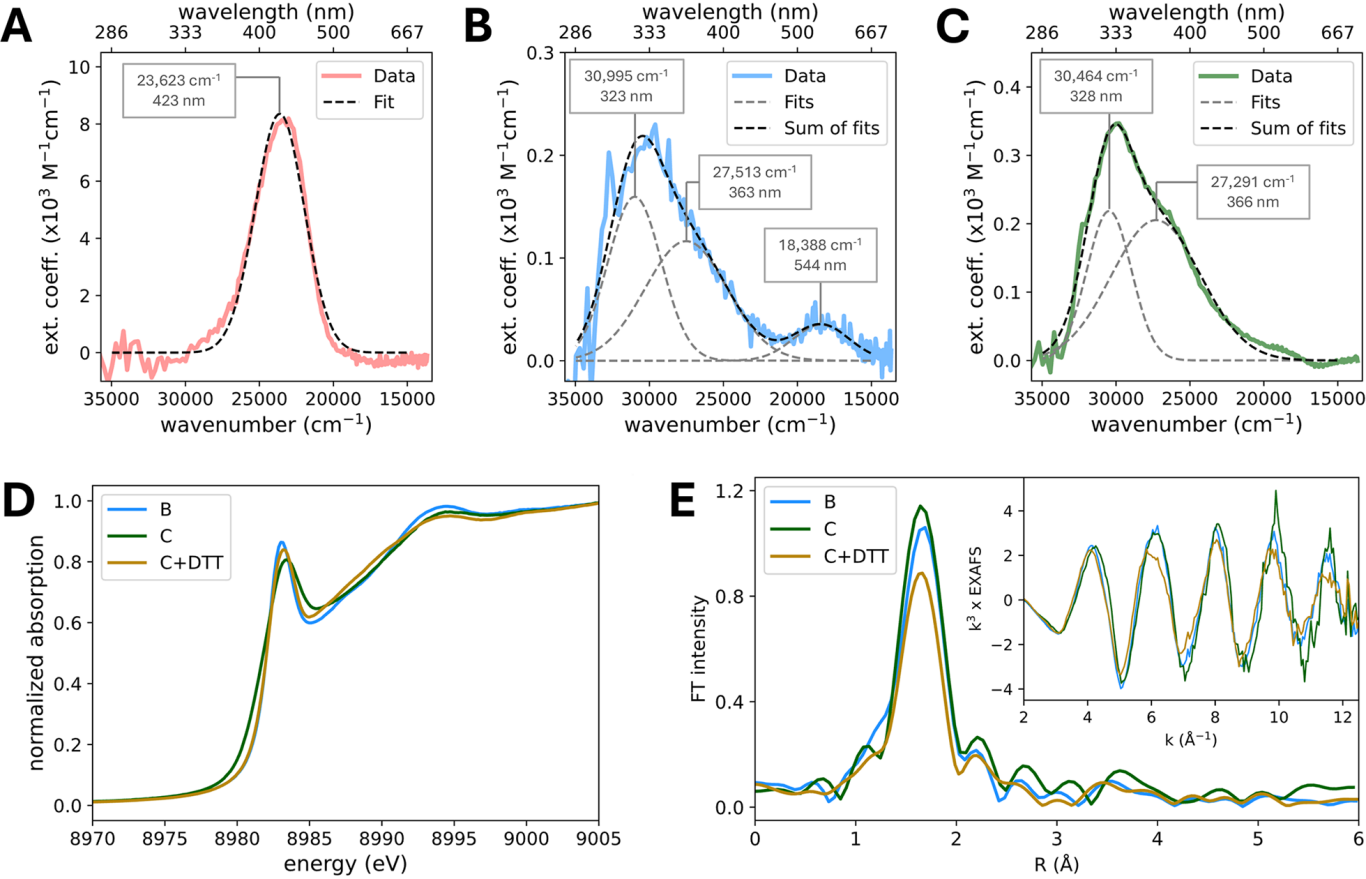
Spectroscopic definition of key intermediates in FGE. (A–C) The UV–vis absorption spectra for (A) intermediate A, (B) intermediate B, and (C) intermediate C obtained from SF-Abs kinetics and corrected for background, with Gaussian fits of their absorption bands (dashed curves; see SI Methods 1.8; results from spectral analysis are summarized in Table S1). (D) Normalized Cu K-edge XANES spectra for intermediate B, intermediate C, and intermediate C after the addition of DTT (C+DTT), and (E) the corresponding EXAFS data (inset) and their non-phase-shift-corrected Fourier transforms.

For intermediates B and C, in addition to obtaining their electronic absorption spectra from SF-Abs experiments, we also cryogenically trapped them for further spectroscopic analysis by EPR and XAS. The EPR spectra of intermediates B and C show that both of these intermediates are EPR-silent (Figure S7), suggesting that each contains either a mononuclear Cu­(I) site or a Cu­(II) site that is magnetically coupled to another spin center (i.e., a radical species). In the Cu K-edge X-ray absorption near edge structure (XANES) region, Cu­(I) complexes show characteristic Cu 1s → 4p transition features at 8983–8984 eV with the normalized absorption amplitude and shape of this pre-edge feature used to probe the coordination geometry of Cu­(I) sites in enzymes.^27^ In our previous study, the normalized absorption amplitudes of the Cu 1s → 4p transition feature of the reduced FGE were observed to be ∼1.0 (characteristic of two-coordinate) and ∼0.77 (three-coordinate) for E and ES, respectively (Figure S8A).^14^ These, together with the extended X-ray absorption fine structure (EXAFS) data analysis (Figure S8B), allowed for the determination of the coordination geometry of the Cu­(I) sites in E and ES (2 Cu–S at 2.14 Å for E and 3 Cu–S at 2.22 Å for ES),^14^ which are in close agreement with the reported crystallographic distances.^14,19^ Figure 3D shows the comparison of the Cu K-edge XANES spectra of intermediates B and C. Both intermediates B and C show a similar pre-edge feature (Cu 1s → 4p transition) at 8983–8984 eV, demonstrating that these both are Cu­(I) species. The normalized absorption amplitudes of this pre-edge feature for intermediates B (∼0.86) and C (similar to or slightly higher than ∼0.86, considering the lower energy resolution for the data of intermediate C; see SI Methods 1.10 and Figure S9) have values in-between those of E (∼1.0) and ES (∼0.77), suggesting that both intermediates contain a two-coordinate Cu­(I) having a weaker third ligand (a “2 + 1” site; see extended discussion in Figure S9).^27^

The Cu K-edge EXAFS data and their Fourier transforms (FT) of intermediates B and C are presented in Figure 3E. The EXAFS and FT data of intermediates B and C do not exhibit significant differences. Close examination shows that the EXAFS spectrum of intermediate B has a slightly higher frequency, which reflects a slightly longer bond distance than intermediate C. Fits to the EXAFS and FT data were systematically performed considering 2-coordinate, 3-coordinate, and “2 + 1”-coordinate models. For intermediate B, the “2 + 1”-coordinate models of 2S + 1S, 2S + 1O, 1S1O + 1S, and 1S1O + 1O give better fits (see the error *F* values of Fit B8–B11 in Table S2) and are consistent with the coordination number obtained from the analysis of the Cu 1s → 4p transition feature. Bond valence sum (BVS) analysis, which uses a library of complexes having known structures and oxidation states to estimate an oxidation state of a metal ion in an unknown complex from each metal–ligand bond, was performed to further evaluate the best EXAFS fits (see SI Methods 1.10 for details on the BVS analysis).^28–31^ The BVS method has been used in the EXAFS analysis in various Cu proteins to correlate the metal oxidation state with the structure.^32–36^ Examination of the error *F* values and the sum of the bond valences (*V*) in the different fits shows that the best fit is obtained with the 1S1O + 1S model (Fit B10 in Table S2; see Figure S10A for this fit), as this fit gives the lowest error *F* value of 0.196 and *V* = 0.98, which is closest to the Cu­(I) oxidation state. Thus, the XANES and EXAFS analyses indicate that the Cu site in intermediate B is in its +1 oxidation state and has a “2 + 1”-coordinate structure with 1 Cu–S at 2.17 Å, 1 Cu–O at 1.95 Å, and 1 Cu–S at 2.80 Å (the high value of *σ* ^2^ for the longer Cu–S bond is acceptable considering the long bond distance). It is noteworthy that inclusion of the Cu–low *Z* atom (i.e., O) interaction is required to obtain a good fit, which is evidenced by comparing Fit B8 (2S + 1S) and Fit B10 (1S1O + 1S) in Table S2, since Fit B8 has the slightly higher error *F* value (0.202 *versus* 0.196) and overestimates the oxidation state (*V* = 1.28 *versus* 0.98).

Fits to the EXAFS and FT data for intermediate C were conducted in the same systematic manner as performed for intermediate B. Since no significant difference is observed in the EXAFS and FT data between intermediates B and C, the best EXAFS fit for intermediate C is also obtained using a “2 + 1”-coordinate model of 1S1O + 1S (lowest error *F* value of 0.310 and *V* = 1.00 for Fit C10 in Table S3; see Figure S10B for this fit). This reveals that the “2 + 1”-coordinate Cu­(I) site in intermediate C has 1 Cu–S at 2.16 Å, 1 Cu–O at 1.95 Å, and 1 Cu–S at 2.77 Å. We note that, as for intermediate B, intermediate C also requires the Cu–low *Z* atom (i.e., O) interaction to have a good fit (Fit C8 (2S + 1S) versus Fit C10 (1S1O + 1S) in Table S3). The best EXAFS fits for intermediates B and C show that while the distances of the two shorter Cu–S and Cu–O bonds are almost the same within error (for intermediates B versus C, 2.17 versus 2.16 Å for Cu–S and 1.95 versus 1.95 Å for Cu–O), the longer Cu–S bond distance is shorter for intermediate C (2.77 Å, versus 2.80 Å for intermediate B).

The Cu K-edge XANES spectrum as well as the EXAFS and FT data for the reduced intermediate C (C+DTT) are also shown in Figure 3D and E, respectively. While the conditions required to prepare this XAS sample (i.e., high enzyme concentration) resulted in incomplete reduction of intermediate C, a qualitative examination of changes in the XANES data upon DTT treatment shows a modest change in the overall spectral shape and a slight decrease in the amplitude of the 8983–8984 eV peak relative to intermediate C (comparison at the same energy resolution), implying changes in the Cu­(I) site. Furthermore, the EXAFS and FT intensities of intermediate C decrease upon DTT treatment reflecting an increase in σ^2^ values and thus, an increase in heterogeneity of the Cu site. These suggest that partial reduction of intermediate C changes the coordination geometry of its Cu­(I) site and results in deviations in the bond distances that can reflect some ligand exchange.

### 2.3 Correlation of Experimental Results to Electronic Structure Calculations

Different reaction coordinates, each with distinct intermediates, have been previously proposed for the catalytic cycle of the FGE. Figure 4A summarizes the different proposed reaction coordinates and the related Cu/O_2_ species. These describe fundamentally different enzymatic reaction mechanisms for the unique mononuclear Cu active site of FGE. The experimental results on intermediates A, B, and C (sections 2.1 and 2.2) were used to systematically evaluate the geometric and electronic structures for a series of candidate species (in Figure 4A), including the previously proposed intermediates.^14,18,19^

**Figure 4:**
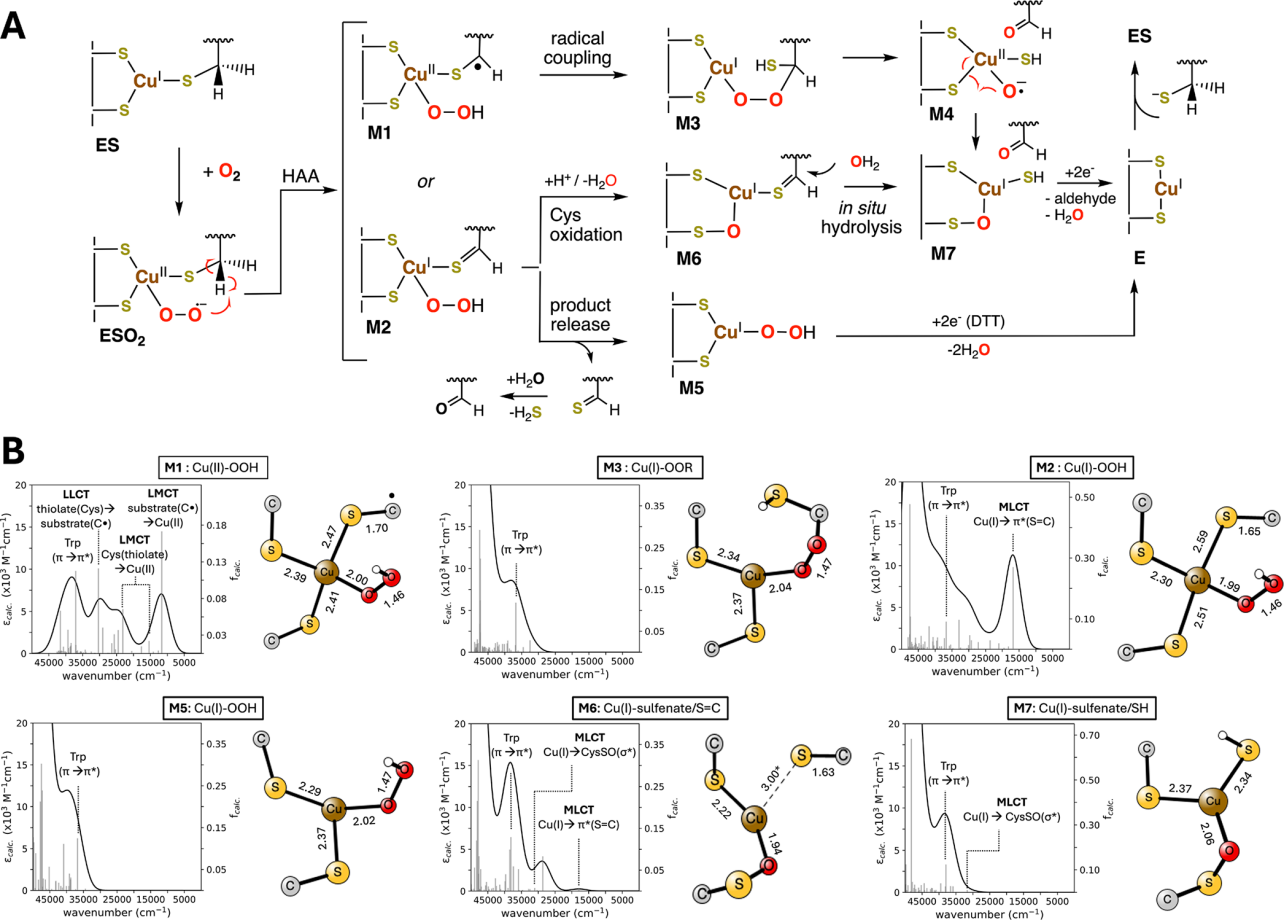
Composite of previously proposed intermediates of FGE and results from DFT/TDDFT calculations. (A) Proposed intermediates for different reaction coordinates after the HAA step. Note that from the results of the present study, intermediate A is assigned as M2, intermediate B is assigned as M6, and intermediate C is assigned as M7. (B) The TD-DFT calculated spectra (camB3LYP/def2TZVP/ε=4.0) for the DFT-optimized structures of proposed intermediates shown in panel A. The TD-DFT transitions are shown as vertical gray lines based on their *f*_calc_ values, and the simulated spectra are shown in black based on their *ε*_calc_ values (see SI Methods 1.12). See Figures S12–19 for extended TD-DFT analyses. Key bond lengths for the first-coordination sphere of selected DFT-optimized structures are shown (Å) next to their associated spectra. The structures for M1–4 were obtained from a previous computational study,^18^ and those for M5–7 were optimized at the B3LYP/def2-SVP/ε=4.0 level of theory (see SI Methods 1.11).

As presented in section 2.1, the presence of a primary KIE on the formation of intermediate A (Figure 2F) indicates that this intermediate forms after the HAA step performed by the putative Cu­(II)–O_2_^•–^ species (ESO_2_ in Figure 4A), which is expected to be a triplet species (S = 1) from previous spectroscopic studies on synthetic model complexes.^37,38^ Consistently, the calculated TD-DFT spectrum for the DFT-optimized structure of the ESO_2_ intermediate contains multiple intense transitions in the UV–vis region (Figure S12), unlike the experimental absorption spectrum for intermediate A (Figure 3A). Thus, for intermediate A, we considered the post-HAA possible intermediates M1, M2, and M3 (Figure 4A). The calculated TD-DFT spectra for M1 (Figure 4B and Figure S13) and M3 (Figure 4B and Figure S14) exclude both as intermediate A, since the former contains multiple intense transitions in the UV–vis region (35 000–15 000 cm^–1^) while the latter lacked the single, lower-energy transition of intermediate A (Figure 3A). In contrast, the TD-DFT spectrum for M2 (Figure 4B and Figure S15) contains a single, lower-energy Cu­(I) → thioaldehyde (π*) transition at an energy (16 766 cm^–1^) and intensity (ε_calc_ = 12 × 10^3^ M^–1^ cm^–1^) that are in general agreement with the one intense absorption band of the experimentally obtained spectrum of intermediate A (23 600 cm^–1^ and 8 × 10^3^ M^–1^ cm^–1^; Figure 3A and Table S1). Therefore, on the basis of both the observed C–H/D KIE in the formation of intermediate A and the correlation of spectroscopic data to TD-DFT calculations, intermediate A is assigned as M2, a Cu­(I)–OOH species with a coordinated thioaldehyde.

For intermediate B, we systematically evaluated the post-HAA intermediates M3, M4, M5, and M6 (Figure 4A) by correlating both their geometric features (i.e., coordination numbers and bond lengths) from their DFT-optimized structures and their electronic structure features from DFT/TD-DFT calculations to the experimental XAS (XANES and EXAFS; Figure 3D and E) and electronic absorption spectra (Figure 3B), respectively. The only structure that reproduces all of the experimental data of intermediate B, including the +1 oxidation state of the Cu site (from XANES; blue spectrum in Figure 3D), the 2S1O (i.e., one light first sphere atom) coordination (from EXAFS; blue spectra in Figure 3E and Table S2), and the UV–vis absorption spectrum (Figure 3B), is M6 (see Extended Analysis S1 for a detailed discussion on this systematic correlation for intermediate B). Notably, the Cu­(I) → thioaldehyde (π*) metal-to-ligand charge transfer (MLCT) transition (17 900 cm^–1^, *f*_calc_ = 6 × 10^–3^) in M6 (Figure 4B and Figure S18) reproduces reasonably well the characteristic lower-energy and weak-intensity absorption band in the experimental spectrum of intermediate B (18 400 cm^–1^, *f*_exp_ = 7 × 10^–4^; Figure 3B). Therefore, intermediate B is assigned as the 2S1O Cu­(I)-sulfenate species with a long Cu­(I)–S­(thioaldehyde) bonding interaction (M6).

As discussed in section 2.2, the XANES and EXAFS results for intermediates B and C indicate that both contain very similar 2S1O Cu­(I) sites (Figure 3D and E). Their UV–vis absorption spectra also exhibit similar high-energy absorption features (Figure 3B and C). However, a key difference between these intermediates is the presence of the low-energy and weak-intensity absorption feature associated with the Cu­(I) → thioaldehyde­(π*) MLCT transition in intermediate B, which is absent in intermediate C (Figure 3B versus C). Starting from the assigned structure for intermediate B (M6), in situ hydrolysis of the thioaldehyde ligand would lead to a HS^–^/sulfenate-coordinated Cu­(I) species (M7 in Figure 4A). The +1 oxidation state and 2S1O coordination of this Cu site are in agreement with the pre-edge of the XANES data (Figure 3D) and the EXAFS analysis (Figure 3E and Table S3), respectively, for intermediate C. The TD-DFT spectrum of M7 lacks any lower-energy features (<29 800 cm^–1^; Figure 4B) consistent with the loss of the Cu­(I) → thioaldehyde (π*) MLCT transition. However, the TD-DFT spectra for both M6 and M7 exhibit Cu­(I) → sulfenate (σ*) MLCT transitions at higher energies and with similar intensities (Figure S18 and Figure S19, respectively) in reasonable agreement with those observed experimentally (Figure 3B and C). Therefore, intermediate C is assigned as the M7 species, with its aldehyde product either fully dissociated or bound to the protein pocket without coordination to Cu­(I). Finally, the XANES and EXAFS results on the partially DTT-reduced intermediate C suggest changes in the Cu­(I) site (Figure 3D and E). This is consistent with the structural changes observed in the in crystallo reaction of the ES complex with O_2_ in the presence of DTT,^15^ showing the partial formation of a bis-thiolate Cu­(I) site with the peptidyl-aldehyde product bound in the protein pocket but not coordinated to Cu­(I). Therefore, the 2e^–^ reduction of intermediate C in solution would result in the loss of its Cu–O interaction along with the loss of the HS^–^ ligand and dissociation of the peptidyl-aldehyde product to restore the bis-thiolate Cu­(I) site of E and restart the catalytic cycle (M7→E, Figure 4A).

## 3 Discussion

This study employed a combination of kinetic, spectroscopic, and computational methods to elucidate the mechanism of the activation of O_2_ and peptidyl-cysteine C–H oxidation by FGE, shown in Figure 5A. The reaction of the ES complex with O_2_ results in the reversible binding of O_2_ to the protein pocket (ES/O_2_) followed by its reversible and endergonic coordination to the metal site (ESO_2_), which performs the HAA from the cysteine C_β_–H of the substrate. While this reactive ESO_2_ intermediate does not accumulate during the single-turnover reaction, the HAA step is now directly observed via the presence of a primary KIE in the formation of intermediate A, which is found to contain a four-coordinate thioaldehyde-bound Cu­(I)–OOH site and exhibit an intense absorption feature at 23 600 cm^–1^ (Figure 3A) associated with its Cu­(I) → thioaldehyde­(π*) CT transition. These results provide further support for the involvement of a Cu­(II)–O_2_^•–^ reactive species as the elusive ESO_2_ intermediate and offer new insights into the O_2_ activation mechanism by the mononuclear Cu­(I) site of the ES complex. Combined with the results from previous crystallographic and spectroscopic studies,^14,15,19^ our study identifies three key mechanistic features that enable O_2_ activation by the mononuclear Cu­(I) site in FGE: (i) the crystallographically observed noncoordinating O_2_ binding in the protein pocket via displacement of a bound water (w1 in E and ES, Figure 1B) to form ES/O_2_ that compensates the entropic cost associated with a bimolecular reaction and increases the local effective concentration of O_2_ near the Cu­(I) site of the ES complex; (ii) the tris-thiolate Cu­(I) site of ES/O_2_ is preorganized for O_2_ coordination to Cu­(I) and formation of the Cu­(II)–O_2_^•–^ species, which is calculated to be 2 kcal/mol more favorable than in the equivalent bis-thiolate Cu­(I) site of E/O_2_ (see DFT analysis in Figure S20); and (iii) in addition to favoring O_2_ activation, peptide binding to E leading to the formation of the ES complex brings the substrate pro-R-hydrogen to an accessible position (O_distal_–H_pro‑R_ = ∼2.5 Å) for the irreversible HAA by the reactive Cu­(II)–O_2_^•–^ species, which provides the required driving force to carry the reaction forward.

**Figure 5:**
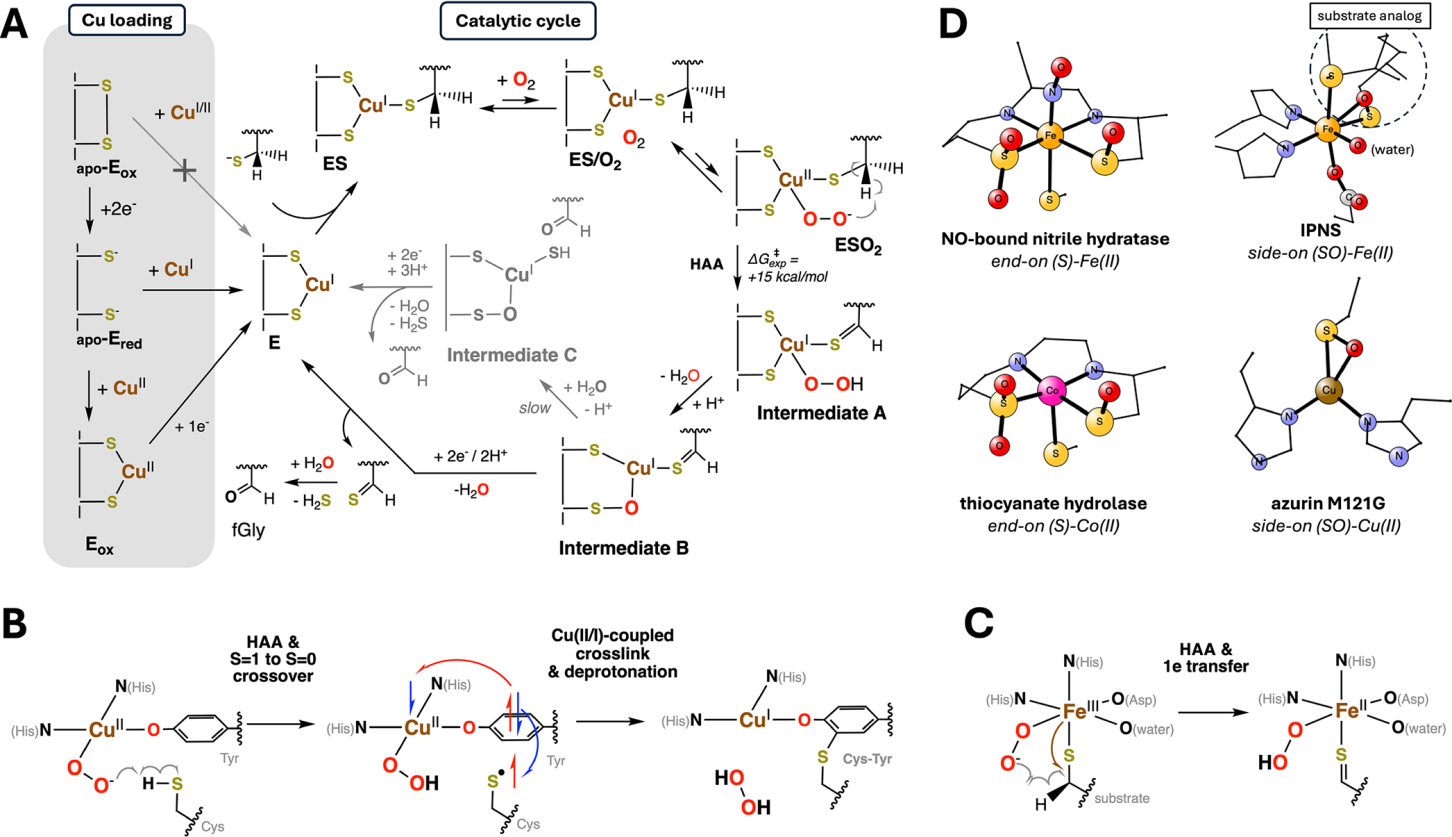
The experimentally supported mechanism of FGE contains an unprecedented end-on sulfenate (S–O)–Cu­(I) coordination. (A) The mechanism of FGE, including the metal loading steps (in gray box, left) and the catalytic cycle for O_2_ activation and peptidyl-cysteine oxidation (right), supported by this study and previous work.^3,13–15^ (B and C) The structurally distinct active sites of (B) the galactose oxidase biogenesis reaction and (C) isopenicillin N synthase exhibit mechanistic parallels with those of FGE. (D) Other known metal–sulfenate centers in biology include (i) the Fe–sulfenate center with an end-on (S)-coordination in the NO-inhibited crystal structure of the nitrile hydratase from *Rhodococcus erythropolis* (PDB: 2AHJ),^52^ (ii) the Co–sulfenate center with an end-on (S)-coordination in the crystal structure of the thiocyanate hydrolase from *Thiobacillus thioparus* (PDB: 2DXB),^53^ (iii) the Fe–sulfenate center with a side-on (SO)-coordination mode in the crystal structure of isopenicillin *N*-synthase from *Aspergillus nidulans* in complex with a substrate analogue (PDB: 2VBB),^44^ and (iv) the Cu­(II)–sulfenate center with side-on (SO)-coordination in azurin M121G,^50^ a type I blue copper protein from *Pseudomonas aeruginosa*.

The all-cysteine-coordinated Cu­(I) site of FGE is structurally similar to that of metallochaperone proteins involved in copper homeostasis (e.g., Atox1, Hah1);^16^ however, these are functionally distinct as they lack any enzymatic activity. This is consistent with the elucidation of the FGE structure defining a novel protein fold.^39^ Interestingly, FGE exhibits notable mechanistic parallels to other O_2_-activating metalloenzymes that are phylogenetically unrelated and contain fundamentally different metal active sites. The Cu­(II)/Cu­(I) redox couple along the HAA step (ESO_2_ → intermediate A, Figure 5A) is also observed to drive the cofactor biogenesis reaction in GaOx^36^ and the initial steps in cysteine substrate oxidation in isopenicillin N synthase (IPNS).^40–43^ In GaOx biogenesis, a Cu­(II)–O_2_^•–^ intermediate abstracts an H atom from a cysteine residue, which then forms a cross-link to a nearby Cu­(II)-coordinated tyrosine residue with the concerted 1e^–^ reduction of the Cu­(II) site (Figure 5B).^36^ In IPNS, the HAA from a metal-coordinated cysteine of the peptidyl substrate by an Fe­(III)-O_2_^•–^ intermediate is coupled to 1e^–^ reduction of the Fe­(III) site and formation of a thioaldehyde ligand (Figure 5C),^40–43^ similar to that in FGE (intermediate A, Figure 5A). However, with its Cu–thiolate ligation, FGE utilizes the ^–^OOH for cysteine oxidation to sulfenate in contrast to formation of the Fe­(IV)–oxo site in IPNS.^44^

Following HAA, the hydroperoxo ligand of the four-coordinate Cu­(I) site in intermediate A proceeds to oxidize one of the protein-derived cysteine ligands to form a three-coordinate sulfenate/thioaldehyde-bound Cu­(I) species (intermediate B, Figure 5A), which is accompanied by a decrease in the energy and intensity of the Cu­(I) → thioaldehyde (π*) MLCT transition (Figure 3A and B) that is associated with the decrease in coordination number (4-coordinate → 3-coordinate) and the increase in the Cu­(I)–S­(thioaldehyde) bond length. From intermediate B, the slow in situ hydrolysis of the thioaldehyde ligand and release of the aldehyde product lead to loss of the Cu­(I) → thioaldehyde (π*) feature (Figure 3C) and formation of a stable three-coordinate HS^–^/sulfenate-bound Cu­(I) species (intermediate C, Figure 5A), which upon its subsequent 2e^–^ reduction, via DTT regenerates the reduced state (E, Figure 5A) and closes the catalytic cycle of FGE. Note that while the physiological reductant of FGE remains to be identified, DTT and glutathione were both shown to be competent reductants.^45^ While the SF-Abs kinetics for intermediates A and B indicate that these are catalytically relevant, the slow formation of intermediate C (Figure 2C and Figure S6) suggests that it is not part of the catalytic cycle of FGE, and it only accumulates in the absence of an external reductant.

Notably, the experimentally validated structures for intermediates B and C contain metal–sulfenate bonds (Figure 5A). While co- and post-translationally cysteine-derived sulfenates are known to play many important roles in biology,^46–48^ metal–sulfenate bonds in enzyme active sites remain exceedingly rare. In fact, to date, only four other proteins have been experimentally found to contain metal–sulfenate bonds with either SO side-on or S end-on coordination (Figure 5D). Recently, a fifth metal–sulfenate bond was proposed for the unusual S = 1 Fe­(IV)–oxo intermediate in the nonheme Fe enzyme, OvoA; however, its specific end-on (S vs O) coordination mode could not be established.^49^ Our EXAFS analysis for intermediates B and C in FGE reveals a light-atom first-shell ligand defining the presence of a Cu­(I)–sulfenate bond with an end-on (O) coordination mode. The functional roles of metal–sulfenate sites in different enzymes and with different coordination modes remain mostly unexplored. In the M121G azurin mutant (Figure 5D), the cysteine ligand is oxidized to sulfenate via the non-native reaction of the Cu­(II) site with hydrogen peroxide and serves as a structural model.^50^ In IPNS (Figure 5D), the sulfenate ligand is generated from the oxidation of a peptidyl-cysteine of a substrate analogue by the Fe­(III)–O_2_^•–^ intermediate and serves as a mechanistic probe. In nitrile hydratase and thiocyanate hydrolase (Figure 5D), the oxidation of the two cysteine ligands to sulfenate and sulfinate is required for the native reactivity, but their respective sulfur oxidation states do not change during catalysis,^51^ unlike the Cu­(I)–sulfenate center in FGE, which is generated from its cysteinate–Cu­(I)–OOH precursor during the catalytic cycle (Figure 5A, right). Our study demonstrates that this cysteinate/sulfenate redox cycling is critical for the O_2_ activation mechanism of FGE, as it enables the temporary storage of the two additional oxidizing equivalents generated during peptidyl-cysteine oxidation (Figure 5A). Previous biochemical work has shown that the metal loading process in FGE also involves redox changes of its cysteine ligands, with disulfide bond reduction required for copper binding to apo-FGE (gray box in Figure 5A).^3,14^

In conclusion, this study employed spectroscopic, kinetic, and computational methods to define a series of key reaction intermediates in FGE, which enabled the elucidation of its catalytic mechanism (Figure 5A). These results provide new fundamental insights into O_2_ activation and cysteine oxidation by mononuclear Cu sites in biology and open the way for exploring structure–function correlations across both other metalloenzymes as well as metal–sulfenate centers with distinct coordination modes and reactivities (Figure 5D). This new mechanistic understanding could further inform and inspire protein engineering and rational design efforts toward expanding the utility and applications of FGE in biotechnology, chemical biology, and biocatalysis.

## Supplementary Material



## Notes

Detailed methods, supporting figures and analysis, and DFT coordinates (PDF)

## ∇

Miller Research Institute for Basic Research, University of California Berkeley, Berkeley, California 94720, United States

## ○

IDEAYA Biosciences, Inc., 5000 Shoreline Ct, Suite 300, South San Francisco, California 94080, United States

## ☆

Department of Chemistry, University of Miami, Miami, Florida 33146, United States

## Abbreviations

Formylglycine-generating enzyme: FGE
formylglycine: fGly
lytic polysaccharide monooxygenases: LPMOs
particulate methane monooxygenase: pMMO
enzyme–substrate complex: ES
enzyme–substrate–O_2_ complex: ESO_2_
kinetic isotope effect: KIE
H atom abstraction: HAA
stopped-flow absorption: SF-Abs
high-performance liquid chromatography: HPLC
dithiothreitol: DTT
electron paramagnetic resonance: EPR
X-ray absorption spectroscopy: XAS
X-ray absorption near edge structure: XANES
extended X-ray absorption fine structure: EXAFS
Fourier transform: FT
bond valence sum: BVS
density functional theory: DFT
time-dependent density functional theory: TD-DFT
charge transfer: CT
ligand-to-metal charge transfer: LMCT
metal-to-ligand charge transfer: MLCT
ligand-to-ligand charge transfer: LLCT
isopenicillin N synthase: IPNS, Galactose oxidase, GaOx

## References

[1] Appel M. J., Bertozzi C. R. (2015). Formylglycine, a
Post-Translationally Generated Residue
with Unique Catalytic Capabilities and Biotechnology Applications. ACS Chem. Biol..

[2] Carrico I. S., Carlson B. L., Bertozzi C. R. (2007). Introducing
Genetically Encoded Aldehydes
into Proteins. Nat. Chem. Biol..

[3] Knop M., Engi P., Lemnaru R., Seebeck F. P. (2015). In Vitro Reconstitution
of Formylglycine-Generating Enzymes Requires Copper­(I). ChemBioChem..

[4] Holder P. G., Jones L. C., Drake P. M., Barfield R. M., Bañas S., De Hart G. W., Baker J., Rabuka D. (2015). Reconstitution of Formylglycine-Generating
Enzyme with Copper­(II) for Aldehyde Tag Conversion. J. Biol. Chem..

[5] Solomon E. I., Heppner D. E., Johnston E. M., Ginsbach J. W., Cirera J., Qayyum M., Kieber-Emmons M. T., Kjaergaard C. H., Hadt R. G., Tian L. (2014). Copper Active Sites
in Biology. Chem. Rev..

[6] Whittaker J. W. (2005). The Radical
Chemistry of Galactose Oxidase. Arch. Biochem.
Biophys..

[7] Mure M., Mills S. A., Klinman J. P. (2002). Catalytic Mechanism
of the Topa Quinone
Containing Copper Amine Oxidases. Biochemistry.

[8] Bissaro B., Røhr Å. K., Müller G., Chylenski P., Skaugen M., Forsberg Z., Horn S. J., Vaaje-Kolstad G., Eijsink V. G. H. (2017). Oxidative Cleavage of Polysaccharides by Monocopper
Enzymes Depends on H_2_O_2_. Nat. Chem. Biol..

[9] Hangasky J. A., Iavarone A. T., Marletta M. A. (2018). Reactivity
of O_2_ versus
H_2_O_2_ with Polysaccharide Monooxygenases. Proc. Natl. Acad. Sci. U.S.A..

[10] Jones S. M., Transue W. J., Meier K. K., Kelemen B., Solomon E. I. (2020). Kinetic
Analysis of Amino Acid Radicals Formed in H_2_O_2_ -Driven Cu^I^ LPMO Reoxidation Implicates Dominant Homolytic
Reactivity. Proc. Natl. Acad. Sci. U.S.A..

[11] Koo C. W., Tucci F. J., He Y., Rosenzweig A. C. (2022). Recovery
of Particulate Methane Monooxygenase Structure and Activity in a Lipid
Bilayer. Science.

[12] Chang W.-H., Lin H.-H., Tsai I.-K., Huang S.-H., Chung S.-C., Tu I.-P., Yu S. S.-F., Chan S. I. (2021). Copper Centers in
the Cryo-EM Structure of Particulate Methane Monooxygenase Reveal
the Catalytic Machinery of Methane Oxidation. J. Am. Chem. Soc..

[13] Knop M., Dang T. Q., Jeschke G., Seebeck F. P. (2017). Copper Is a Cofactor
of the Formylglycine-Generating Enzyme. ChemBioChem..

[14] Appel M. J., Meier K. K., Lafrance-Vanasse J., Lim H., Tsai C.-L., Hedman B., Hodgson K. O., Tainer J. A., Solomon E. I., Bertozzi C. R. (2019). Formylglycine-Generating Enzyme Binds Substrate Directly
at a Mononuclear Cu­(I) Center to Initiate O_2_ Activation. Proc. Natl. Acad. Sci. U.S.A..

[15] Leisinger F., Miarzlou D. A., Seebeck F. P. (2021). Non-Coordinative
Binding of O_2_ at the Active Center of a Copper-Dependent
Enzyme. Angew. Chem. Int. Ed.

[16] Rosenzweig A. C. (2001). Copper
Delivery by Metallochaperone Proteins. Acc.
Chem. Res..

[17] Meury M., Knop M., Seebeck F. P. (2017). Structural Basis for Copper-Oxygen
Mediated C-H Bond Activation by the Formylglycine-Generating Enzyme. Angew. Chem..

[18] Wu Y., Zhao C., Su Y., Shaik S., Lai W. (2023). Mechanistic
Insight into Peptidyl-Cysteine Oxidation by the Copper-Dependent Formylglycine-Generating
Enzyme. Angew. Chem. Int. Ed.

[19] Miarzlou D. A., Leisinger F., Joss D., Häussinger D., Seebeck F. P. (2019). Structure of Formylglycine-Generating
Enzyme in Complex
with Copper and a Substrate Reveals an Acidic Pocket for Binding and
Activation of Molecular Oxygen. Chem. Sci..

[20] Bhadra M., Transue W. J., Lim H., Cowley R. E., Lee J. Y. C., Siegler M. A., Josephs P., Henkel G., Lerch M., Schindler S., Neuba A., Hodgson K. O., Hedman B., Solomon E. I., Karlin K. D. (2021). A Thioether-Ligated Cupric Superoxide
Model with Hydrogen Atom Abstraction Reactivity. J. Am. Chem. Soc..

[21] Ginsbach J. W., Peterson R. L., Cowley R. E., Karlin K. D., Solomon E. I. (2013). Correlation
of the Electronic and Geometric Structures in Mononuclear Copper­(II)
Superoxide Complexes. Inorg. Chem..

[22] Peterson R.
L., Ginsbach J. W., Cowley R. E., Qayyum M. F., Himes R. A., Siegler M. A., Moore C. D., Hedman B., Hodgson K. O., Fukuzumi S., Solomon E. I., Karlin K. D. (2013). Stepwise Protonation
and Electron-Transfer Reduction of a Primary Copper-Dioxygen Adduct. J. Am. Chem. Soc..

[23] Debnath S., Laxmi S., McCubbin
Stepanic O., Quek S. Y., Van Gastel M., DeBeer S., Krämer T., England J. (2024). A Four-Coordinate End-On
Superoxocopper­(II) Complex: Probing the Link between Coordination
Number and Reactivity. J. Am. Chem. Soc..

[24] Quist D. A., Diaz D. E., Liu J. J., Karlin K. D. (2017). Activation of Dioxygen
by Copper Metalloproteins and Insights from Model Complexes. J. Biol. Inorg. Chem..

[25] Cowley R. E., Tian L., Solomon E. I. (2016). Mechanism
of O_2_ Activation
and Substrate Hydroxylation in Noncoupled Binuclear Copper Monooxygenases. Proc. Natl. Acad. Sci. U.S.A..

[26] Zhu H., Sommerhalter M., Nguy A. K. L., Klinman J. P. (2015). Solvent and Temperature
Probes of the Long-Range Electron-Transfer Step in Tyramine β-Monooxygenase:
Demonstration of a Long-Range Proton-Coupled Electron-Transfer Mechanism. J. Am. Chem. Soc..

[27] Kau L. S., Spira-Solomon D. J., Penner-Hahn J. E., Hodgson K. O., Solomon E. I. (1987). X-Ray Absorption
Edge Determination of the Oxidation State and Coordination Number
of Copper. Application to the Type 3 Site in Rhus Vernicifera Laccase
and Its Reaction with Oxygen. J. Am. Chem. Soc..

[28] Brown I. D., Altermatt D. (1985). Bond-Valence Parameters Obtained from a Systematic
Analysis of the Inorganic Crystal Structure Database. Acta Crystallogr. B Struct Sci..

[29] Brese N. E., O’Keeffe M. (1991). Bond-Valence
Parameters for Solids. Acta Crystallogr. B Struct
Sci..

[30] Thorp H. H. (1992). Bond Valence
Sum Analysis of Metal-Ligand Bond Lengths in Metalloenzymes and Model
Complexes. Inorg. Chem..

[31] Liu W., Thorp H. H. (1993). Bond Valence Sum
Analysis of Metal-Ligand Bond Lengths
in Metalloenzymes and Model Complexes. 2. Refined Distances and Other
Enzymes. Inorg. Chem..

[32] Dooley D. M., Scott R. A., Knowles P. F., Colangelo C. M., McGuirl M. A., Brown D. E. (1998). Structures of the
Cu­(I) and Cu­(II)
Forms of Amine Oxidases from X-Ray Absorption Spectroscopy. J. Am. Chem. Soc..

[33] Osborne J. P., Cosper N. J., Stälhandske C.
M. V., Scott R. A., Alben J. O., Gennis R. B. (1999). Cu XAS Shows a Change in the Ligation
of Cu_B_ upon Reduction of Cytochrome *Bo*
_3_ from *Escherichia Coli*. Biochemistry.

[34] Shearer J., Szalai V. A. (2008). The Amyloid-β
Peptide of Alzheimer’s Disease
Binds Cu ^I^ in a Linear Bis-His Coordination Environment:
Insight into a Possible Neuroprotective Mechanism for the Amyloid-β
Peptide. J. Am. Chem. Soc..

[35] Arcos-López T., Qayyum M., Rivillas-Acevedo L., Miotto M. C., Grande-Aztatzi R., Fernández C. O., Hedman B., Hodgson K. O., Vela A., Solomon E. I., Quintanar L. (2016). Spectroscopic and Theoretical Study
of Cu^I^ Binding to His111 in the Human Prion Protein Fragment
106–115. Inorg. Chem..

[36] Cowley R. E., Cirera J., Qayyum M. F., Rokhsana D., Hedman B., Hodgson K. O., Dooley D. M., Solomon E. I. (2016). Structure of the
Reduced Copper Active Site in Preprocessed Galactose Oxidase: Ligand
Tuning for One-Electron O_2_ Activation in Cofactor Biogenesis. J. Am. Chem. Soc..

[37] Woertink J. S., Tian L., Maiti D., Lucas H. R., Himes R. A., Karlin K. D., Neese F., Würtele C., Holthausen M. C., Bill E., Sundermeyer J., Schindler S., Solomon E. I. (2010). Spectroscopic and Computational Studies
of an End-on Bound Superoxo-Cu­(II) Complex: Geometric and Electronic
Factors That Determine the Ground State. Inorg.
Chem..

[38] Lanci M. P., Smirnov V. V., Cramer C. J., Gauchenova E. V., Sundermeyer J., Roth J. P. (2007). Isotopic Probing of Molecular Oxygen
Activation at Copper­(I) Sites. J. Am. Chem.
Soc..

[39] Dierks T., Dickmanns A., Preusser-Kunze A., Schmidt B., Mariappan M., Von Figura K., Ficner R., Rudolph M. G. (2005). Molecular Basis
for Multiple Sulfatase Deficiency and Mechanism for Formylglycine
Generation of the Human Formylglycine-Generating Enzyme. Cell.

[40] Brown C. D., Neidig M. L., Neibergall M. B., Lipscomb J. D., Solomon E. I. (2007). VTVH-MCD
and DFT Studies of Thiolate Bonding to {FeNO}^7^/{FeO_2_}^8^ Complexes of Isopenicillin *N* Synthase: Substrate Determination of Oxidase versus Oxygenase Activity
in Nonheme Fe Enzymes. J. Am. Chem. Soc..

[41] Brown-Marshall C. D., Diebold A. R., Solomon E. I. (2010). Reaction Coordinate of Isopenicillin
N Synthase: Oxidase versus Oxygenase Activity. Biochemistry.

[42] Lundberg M., Siegbahn P. E. M., Morokuma K. (2008). The Mechanism for Isopenicillin N
Synthase from Density-Functional Modeling Highlights the Similarities
with Other Enzymes in the 2-His-1-Carboxylate Family. Biochemistry.

[43] Tamanaha E., Zhang B., Guo Y., Chang W., Barr E. W., Xing G., St. Clair J., Ye S., Neese F., Bollinger J. M., Krebs C. (2016). Spectroscopic Evidence
for the Two
C-H-Cleaving Intermediates of *Aspergillus Nidulans* Isopenicillin *N* Synthase. J. Am. Chem. Soc..

[44] Ge W., Clifton I. J., Stok J. E., Adlington R. M., Baldwin J. E., Rutledge P. J. (2008). Isopenicillin N
Synthase Mediates
Thiolate Oxidation to Sulfenate in a Depsipeptide Substrate Analogue:
Implications for Oxygen Binding and a Link to Nitrile Hydratase?. J. Am. Chem. Soc..

[45] Ennemann E. C., Radhakrishnan K., Mariappan M., Wachs M., Pringle T. H., Schmidt B., Dierks T. (2013). Proprotein Convertases Process and
Thereby Inactivate Formylglycine-Generating Enzyme*. J. Biol. Chem..

[46] Paulsen C. E., Carroll K. S. (2013). Cysteine-Mediated
Redox Signaling: Chemistry, Biology,
and Tools for Discovery. Chem. Rev..

[47] Michalek R.
D., Nelson K. J., Holbrook B. C., Yi J. S., Stridiron D., Daniel L. W., Fetrow J. S., King S. B., Poole L. B., Grayson J. M. (2007). The Requirement of Reversible Cysteine Sulfenic Acid
Formation for T Cell Activation and Function. J. Immunol..

[48] Poole L. B. (2003). Formation
and Functions of Protein Sulfenic Acids. Curr.
Protoc. Toxicol..

[49] Paris J. C., Hu S., Wen A., Weitz A. C., Cheng R., Gee L. B., Tang Y., Kim H., Vegas A., Chang W., Elliott S. J., Liu P., Guo Y. (2023). An *S* = 1 Iron­(IV) Intermediate Revealed in a Non-Heme
Iron Enzyme-Catalyzed
Oxidative C-S Bond Formation. Angew. Chem. Int.
Ed.

[50] Sieracki N. A., Tian S., Hadt R. G., Zhang J.-L., Woertink J. S., Nilges M. J., Sun F., Solomon E. I., Lu Y. (2014). Copper-Sulfenate
Complex from Oxidation of a Cavity Mutant of *Pseudomonas Aeruginosa* Azurin. Proc. Natl. Acad. Sci. U.S.A..

[51] Murakami T., Nojiri M., Nakayama H., Dohmae N., Takio K., Odaka M., Endo I., Nagamune T., Yohda M. (2000). Post-translational
Modification Is Essential for Catalytic Activity of Nitrile Hydratase. Protein Sci..

[52] Nagashima S., Nakasako M., Dohmae N., Tsujimura M., Takio K., Odaka M., Yohda M., Kamiya N., Endo I. (1998). Novel Non-Heme Iron Center of Nitrile
Hydratase with a Claw Setting
of Oxygen Atoms. Nat. Struct Mol. Biol..

[53] Arakawa T., Kawano Y., Katayama Y., Nakayama H., Dohmae N., Yohda M., Odaka M. (2009). Structural Basis for Catalytic Activation
of Thiocyanate Hydrolase Involving Metal-Ligated Cysteine Modification. J. Am. Chem. Soc..

