# Supplementary material for: Mechanism of O2 Activation and Cysteine Oxidation by the Unusual Mononuclear Cu(I) Active Site of the Formylglycine-Generating Enzyme
Source: ACS Cent Sci. 2025 Apr 4;11(5):683–93. doi: 10.1021/acscentsci.5c00183 (PMC12123546; doi:10.1021/acscentsci.5c00183)
Supplement: Supplementary file 1 [file oc5c00183_si_001.pdf]

Supplementary Information for

Mechanism of O_2 activation and cysteine oxidation by the unusual mononuclear Cu(I) active site of the formylglycine-generating enzyme

Ioannis Kipouros[a], Hyeongtaek Lim[a], Mason J. Appel[a,b], Katlyn K. Meier[a], Britt Hedman[c], Keith O. Hodgson[a,c], Carolyn R. Bertozzi[a,d*], and Edward I. Solomon[a,c,*]

[a] Department of Chemistry, Stanford University, Stanford, CA 94305, [b] Department of Molecular and Cell Biology, University of California, Berkeley, CA 94720, [c] Stanford Synchrotron Radiation Lightsource, SLAC National Accelerator Laboratory, Stanford University, Menlo Park, CA 94025, [d] Howard Hughes Medical Institute, Stanford University, Stanford, CA 94305

Corresponding authors:
E.I.S.: solomone@stanford.edu
C.R.B.: bertozzi@stanford.edu,

SI Table of Contents

1. Materials & Methods

1.1. *General safety comments* S3

1.2. *Chemicals* S3

1.3. *Peptide synthesis & characterization* S3

1.4. *Molecular biology, protein expression & purification* S4

1.5. *Anaerobic constitution of the FGE-Cu(I)-peptide complex* S4

1.6. *Rapid chemical-quench kinetics* S5

1.7. *Stopped-flow absorption (SF-Abs) data collection & analysis:* S5

1.8. *Spectral analysis of the UV-Vis absorption data* S5

1.9. *Electron paramagnetic resonance (EPR) spectroscopy* S6

1.10. *X-ray absorption spectroscopy (XAS)* S6

1.11. *Density functional theory (DFT) calculations* S8

1.12. *Time-dependent density functional theory (TD-DFT) calculations & spectral simulations* S8

2. SI Figures & Analysis

Scheme S1. *Structure of the isotopically labeled peptide substrate used in this study* S4

Figure S1. *Product formation kinetics measured by rapid chemical quench* S10

Figure S2. *Reaction of intermediate C (with or without prior DTT treatment) with O_2* S11

Figure S3. *Kinetic fits for intermediate A assigned to the Cu(II)-$O_2^{\bullet-}$ (ESO_2) species* S12

Figure S4. *Determining the O_2 binding constant (K_d) range for the FGE/Cu(I)/peptide (ES) complex* S13

Scheme S2. *Simplified & extended kinetic schemes* S15

Figure S5. *Extended kinetic isotope effect (KIE) analysis* S16

Figure S6. *Kinetic fitting & time-course speciation of the conversion of intermediate B to C* S17

Table S1. *Summary of results from spectral analysis of intermediates A, B, and C* S18

Figure S7. *EPR spectra of intermediates B and C* S19

Figure S8. *Comparison of XANES/EXAFS spectra* S20

Figure S9. *XANES spectral broadening for intermediates B* S21

Figure S10. *EXAFS data with best fits for intermediates B and C* S22

Figure S11. *Normalized Cu K-edge XANES spectra of a Cu foil* S22

Table S2. *EXAFS fitting results and BVS values for intermediate B* S23

Table S3. *EXAFS fitting results and BVS values for intermediate C* S24

Figure S12. *Extended TD-DFT analysis for the ESO_2 (S=1) species* S25

Figure S13. *Extended TD-DFT analysis for the M1 species* S27

Figure S14. *Extended TD-DFT analysis for the M3 species* S29

Figure S15. *Extended TD-DFT analysis for the M2 species* S31

Extended Analysis S1. *Systematic correlation of model M3-6 to intermediate B* S33

Figure S16. *The DFT-optimized structure & extended TD-DFT analysis for the M4 species* S34

Figure S17. *Extended TD-DFT analysis for the M5 species* S36

Figure S18. *Extended TD-DFT analysis of M6 & evaluation of the Cu(I)-S(thiolate) bond distance* S37

Figure S19. *Extended TD-DFT analysis for the M7 species* S40

Figure S20. *DFT calculations for O_2 binding & activation by E vs ES* S41

3. SI References S43

4. SI Data: *Cartesian coordinates for DFT-optimized structures* S45

1.1. General safety comments: No experiments used particularly dangerous chemicals. The rapid chemical quench experiments (SI Methods 1.6) used concentrated HCl, which is a highly corrosive acid. The preparation and storing of samples for XAS and EPR experiments involved the use of cryogens (liquid nitrogen, 77 K) which requires specialized training, careful handling and protection. The collection of the XAS and EPR data also involved other cryogens (liquid helium, <10 K). The collection of the XAS data (SI Methods 1.10) involved high-power X-ray radiation experiments that require specialized training and radiation safety protocols.

1.2. Chemicals: All aqueous solutions were made with water purified to a resistivity >17 MΩ cm^{-1} using a Barnstead Nanopure deionizer. All chemicals were purchased from Sigma unless noted otherwise.

1.3. Peptide synthesis & characterization: The peptide synthetic procedures were carried out as previously described,[1] and restated in the following text. The substrates were synthesized manually on Fmoc-Rink Amide MBHA low loading resin (EMD Millipore) using commercially available N_α-Fmoc protected amino acid monomers. A 3,3-D_2-Fmoc-L-cys(S-trityl)-OH monomer with an isotopic enrichment of 99.6% (Cambridge Isotope Laboratories) was used to synthesize the isotopically labeled peptide (Scheme S1) used for the stopped-flow and rapid-quench KIE experiments. A 2- to 10-fold molar excess of amino acid monomer was used in each coupling step with reaction times of 30 min up to overnight with agitation by N_2 gas. Fmoc groups were deprotected by treatment with 20% piperidine in DMF for 15 min. All peptides used herein were amidated at the C-terminus, and the N-terminus was acetylated with acetic anhydride. Following peptide synthesis, peptides were cleaved from solid phase and side chains globally deprotected using a cocktail containing 94:2.5:2.5:1TFA/H_2O/ethane dithiol/triisopropylsilane for 3 h under N_2. The cleavage mixture was filtered to remove resin, concentrated under vacuum, and triturated by dropwise addition to Et_2O at 4 °C. Precipitated crude peptide was collected by centrifugation and washed twice more with diethyl ether prior to dissolution in H_2O and lyophilization. Crude peptides were purified using reverse-phase chromatography with a gradient of 20-30% MeCN/water + 0.1% TFA over 60 min on a Microsorb 100-5 C18 Dynamax 250 x 21.4 mm column (Agilent Technologies). Peptides were characterized by HPLC/UV-vis to be of greater than 98% purity. Unlabeled and deuterated peptide solutions were quantified prior to all experiments by absorbance at 280 nm.

Peptides were sequenced using liquid chromatography-electrospray ionization spectrometry. Purified peptides were diluted to 0.5 µM with 0.1 % formic acid. 1 µL (500 fmol) of each peptide was injected on an Acclaim PepMap RSLC C18 column (Thermo Fisher Scientific) using a Dionex UtiliMate 3000 HPLC (Thermo Fisher Scientific) at a flow rate of 0.3 µL/min, connected in-line to an Orbitrap Fusion mass spectrometer (Thermo Fisher Scientific). Instrument method parameters were as follows: MS1 resolution: 60,000 at 400 m/z; scan range: 300−1500 m/z. The most abundant ions (top speed at n = 3 seconds) were subjected to higher-energy collision induced dissociation (HCD), collision induced dissociation (CID), and electron transfer dissociation (ETD). HCD parameters: 30% normalized collision energy; CID parameters: activation q 0.25, 35% normalized collision energy; ETD parameters: calibrated ETD reaction times, AGC reagent target at 10^5. For all MS2s, isolation width was set at 2 m/z. Dynamic exclusion was enabled with a repeat count of 3, a repeat duration of 10 s, and an exclusion duration of 10 s. Data analysis was accomplished using the Xcalibur software (Thermo Fisher Scientific).

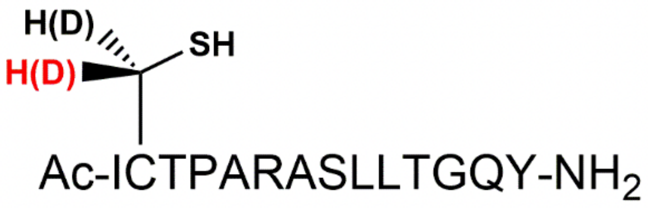

Scheme S1. Structure of the isotopically labeled peptide substrate used in this study. The pro-(R)-β-hydrogen is highlighted in red.

1.4. Molecular biology, protein expression & purification: The molecular biology, and protein expression and purification methods were followed as previously described,[1] and restated in the following text. A *Streptomyces coelicolor* FGE construct was previously cloned into the pET151 expression vector.[2] Expression and purification of FGE were performed as follows. Single clones of *Escherichia coli* BL21 DE3 cells (New England Biolabs) transformed with pET151-scFGE were used to inoculate 250 mL of LB with ampicillin and grown for 16 hours at 37 ºC. Terrific broth containing ampicillin and glycerol was inoculated at a ratio of 40:1 with overnight culture and grown to OD 0.4–0.6 at 37 ºC. Cultures were cooled to 18 ºC and induced with isopropyl-α-thiogalactoside at a final concentration of 0.1-1 mM. Following 18-20 h of growth, cells were harvested by centrifugation at 5000 x g for 30 min at 4 ºC. Cell pellets were resuspended with lysis buffer (0.5 M NaCl, 50 mM Tris-HCl pH 8.0, 25 mM imidazole, 1 mM tris(2-carboxyethyl)phosphine hydrochloride, 10% glycerol (v/v)) at approximately 5 mL per 1 g of cell pellet, 1:10000 Pierce Universal Nuclease (v/v) (Thermo Fisher Scientific), and with 1 EDTA-free protease inhibitor cocktail tablet (Roche) per 50 mL of resuspension. The cell suspension was first homogenized to a single-cell suspension with a Dounce homogenizer. Then cell lysis was accomplished by 3 passes through an Emulsiflex C3 homogenizer (Avestin) at 15 kPa. Lysate was cleared by centrifugation at 20,000 x g for 45 min at 4 ºC. The supernatant was applied to a 5 mL HisTrap HP Ni-NTA column (GE Life Science) connected to an Akta Pure FPLC (GE Healthcare Life Sciences). Flow through was monitored by A280 and washed with lysis buffer until absorbance reached baseline. His6-tagged protein was eluted with a gradient of 0-50% Elution buffer (Lysis buffer containing 0.5 M imidazole) over 10 column volumes. The N-terminal His6 tag was removed from combined fractions by cleavage with Tobacco Etch Virus (TEV) protease at a ratio of 0.02-0.05 (w/w) to FGE overnight at 4 ºC, followed by TEV removal with a subtractive Ni-NTA column. TEV protease was isolated in-house using a published procedure.[3] His6-free FGE was concentrated and further purified by gel-filtration chromatography on a Superdex 75 HiLoad column (GE Healthcare Life Sciences) in storage buffer (50 mM Tris-HCl pH 7.5, 0.5 M NaCl, 1 mM dithiothreitol (DTT), 10% glycerol (v/v)). The concentration of purified proteins was determined by absorbance at 280 nm under denaturing conditions, using the method of Gill and von Hippel.[4] Typical final purity was greater than or equal to 90% and the final yield was approximately 30 mg/L of *E. coli* culture. Proteins were aliquoted, flash frozen with liquid N_2, and stored at -80 ºC until use.

1.5. Anaerobic constitution of the FGE-Cu(I)-peptide complex: The anaerobic reconstitution procedures were followed as previously described,[1] and restated in the following text. Solutions of the FGE enzyme and buffer were transferred into an anaerobic glovebox under N_2 atmosphere

to equilibrate overnight. To remove DTT and adjust the pH, FGE was exchanged from storage buffer into 50 mM Tris-HCl pH 9.0, 0.5 M NaCl, 10% glycerol (v/v) using pre-equilibrated Bio-Spin 6 buffer exchange spin columns (Bio-Rad Laboratories). The buffer-exchanged protein was reconstituted at 4 °C in pH 9.0 buffer with 1:1 FGE:Cu(I) at a final concentration of 0.5 mM each using a freshly prepared 10 mM $Cu(MeCN)_4{}^+PF_6$ stock in anhydrous MeCN. A second buffer exchange step removed unbound Cu(I) and MeCN. FGE was concentrated as necessary using 10 kDa molecular weight cutoff spin filters (EMD Millipore). This procedure yielded Cu(I)- FGE with 98-99 mol % copper as measured by the colorimetric biquinoline assay.[5]

1.6. Rapid chemical-quench kinetics: To prepare the instrument, O_2 was removed from the loading and rapid mixing compartments by incubating these compartments and their solvent lines with a solution of sodium dithionite ($Na_2S_2O_4$) for 45 minutes. Excess $Na_2S_2O_4$ solution was removed by flushing with degassed 50 mM Tris-HCl pH 9.0, 0.5 M NaCl, 10% glycerol (v/v) buffer transferred and loaded with gastight syringes. Next, solutions of the anaerobic Cu(I)-FGE:peptide complex (1:1, 750 μM each, prepared in the glovebox) and O_2-saturated buffer (at 4 °C, approximately 2.2 mM) were prepared separately in pH 9.0 buffer, loaded into gastight syringes, and attached to a RQF-3 rapid mixing instrument (Kintek Corporation). For single-turnover kinetic assays, syringes 1 and 2 were combined to react for a programmed mixing time and ejected into a vessel containing a quench solution of HCl at a final concentration of 100 mM. Formation of the formylglycine product in acid-quenched reactions was measured by HPLC. All rapid mixing experiments were performed at 4 °C, maintained by a chilled recirculating bath.

1.7. Stopped-flow absorption (SF-Abs) data collection & analysis: SF-Abs data were collected using an Applied Photophysics SX.19 SF-Abs spectrophotometer equipped with a Hg/Xe arc lamp, a PDA1 photodiode array detector, and a cell path length of 1 cm. The SF-Abs mixing chamber was kept in a purge box (Cleatech Isolation Glove Box 2100) equipped with an O_2 sensor (Neutronics Model 1100) and maintained under an Argon atmosphere. Both injector ports of the stopped-flow apparatus were degassed with a solution of sodium dithionite (~3.0 mM) for ~20 min and then washed with the degassed buffer thoroughly before use. The temperature of all SF-Abs experiments was kept constant at 4 °C using a water temperature bath (Fisher Scientific Isotemp 3016). All anaerobic solutions were loaded into gastight syringes (Hamilton) inside a glovebox and were loaded in the stopped-flow instrument using three-way valves to prevent O_2 contamination.

Kinetic fitting and analysis of the time-resolved spectra from SF-Abs experiments were analyzed in COPASI (v.4.27-build 217) using the kinetic models shown in Scheme S2. Fitting of kinetic traces from wavelengths corresponding to the growth and decay of absorption features associated with reaction intermediates was performed using the "Parameter estimation" functionality of COPASI and the "Particle swarm" fitting method. In the cases of simultaneously fitting multiple experiments (*i.e.,* for the [O_2]-dependence kinetics shown in Fig.2E of the main text and Fig.S4, and the C-H/D KIE experiments shown in Fig.2F of the main text and Fig.S5), the fitting was carried out by setting all parameters as shared between experiments except the experimental variables or kinetic parameters of interest (*i.e.,* the $[O_2]_{t=0}$ experimental variable in the [O_2]-dependence kinetics fitting, and the specific rate constant variables in the KIE fitting).

1.8. Spectral analysis of the UV-Vis absorption data: Since the chromophoric species in the SF-Abs kinetics are temporally resolved, their spectral contributions were deconvoluted by employing the time-course speciation plots generated from COPASI (Fig.2G in the main text, and

Fig.S6). To obtain the electronic absorption spectra for intermediates A, B, and C (Fig.3A-C in the main text) the following procedure was followed: (i) background contributions (including that from the ~280 nm protein band) were corrected by subtracting the first time-point (at 1-2 ms), which contains only non-chromophoric Cu/O_2 species, (ii) the spectrum of intermediate B (100% accumulation at 13 s) was obtained, and (iii) the spectra for intermediates A and C were obtained at the 0.6 and 1000 s, respectively, after subtraction of spectral contributions from the minor presence of intermediate B at those reaction timepoints (15% and 4%, respectively), and (iv) intensity normalization was performed. The Gaussian fitting was performed utilizing the curve_fit function from the SciPy package in Python. The f_{exp} was calculated using equation S1:

$$f_{exp} = 4.33 * 10^{-9} * AUC \quad [S1]$$

Where area-under-curve (AUC) for a Gaussian curve is calculated from equation S2:

$$AUC = 1.065 * FWHM * \varepsilon_{exp} \quad [S2]$$

1.9. Electron paramagnetic resonance (EPR) spectroscopy: The EPR samples for intermediates B and C were prepared by 1:1 (v/v) mixing of anaerobic solutions of the FGE ES complex (~1.0 mM, pre-mix) in 50 mM Tris, 0.5 M NaCl, pH 9.0 with O_2-saturated (~1.0 mM, pre-mix) solutions of the same buffer at 4 °C. At the selected reaction timepoints corresponding to a high accumulation of each intermediate, ~0.15 mL of the reaction mixture was transferred to an EPR tube and frozen in liquid N_2. Samples were stored in liquid N_2 until data collection.

EPR data were collected as previously described.[1] X-band EPR spectra were obtained with a Bruker EMX spectrometer, an ER 041 XG microwave bridge, and an ER4116DM cavity. Samples were measured at 77 K in a liquid N_2 finger dewar. EPR settings were as follows: Freq. ≈ 9.6 GHz, Power ≈ 10 mW, Mod. Amp. = 4.00 G. All spectra were averaged over 10 scans. EPR spin quantitation of the paramagnetic copper content was performed using a 0.945 mM AAS Cu standard solution, Specpure (purchased from Alfa Aesar) in MES (pH 6.0) and 40% glycerol. EPR spectra were simulated using the SpinCount simulation software.[6]

1.10. X-ray absorption spectroscopy (XAS): The XAS samples for intermediates B and C were prepared by 1:1 (v/v) mixing of anaerobic solutions of the FGE ES complex (~1.0 and ~0.75 mM for intermediates B and C, respectively) in 50 mM Tris, 0.5 M NaCl, pH 9.0 with O_2-saturated solutions in 50 mM Tris, 0.5 M NaCl, 10% (v/v) glycerol, pH 9.0 at 4 °C. At the selected reaction timepoints corresponding to the high accumulation of each intermediate, the reaction mixture was loaded into delrin XAS sample holders with 38 μm Kapton tape windows and stored in liquid N_2 until data collection. The sample of intermediate C treated with DTT (C+DTT) (~0.7 mM ES) was prepared similarly as above, in which, before cryogenic trapping, the reaction mixture was equilibrated with 2.0 equivalents (relative to enzyme concentration) of DTT.

The Cu K-edge XAS data were collected at the Stanford Synchrotron Radiation Lightsource (SSRL) on the 16-pole 2 T wiggler side-station beamline 9–3 under the ring conditions of 3 GeV and ~500 mA. A Si(220) double-crystal monochromator was used for energy selection.

A Rh-coated M_0 mirror was used for harmonic rejection and vertical collimation, and a cylindrical Rh-coated M_1 mirror was used for beam focusing. The samples were maintained at a constant temperature of ~10 K during data collection using an Oxford Instruments liquid He cryostat. A Canberra 100-element Ge monolith solid-state detector and Soller slits equipped with a Ni filter were used to collect Cu Kα fluorescence data. Internal energy calibration was accomplished by simultaneous measurement of the absorption of a Cu foil placed between two ionization chambers situated after the sample. The first inflection point of the Cu foil spectrum was assigned to 8980.3 eV. The EAXFS data are reported to k = 12.8 $Å^{-1}$ to avoid interference from possible Zn contamination. Photodamage and photoreduction were not observed during the course of data collection. The XAS data presented in this study include an average of 6 scans, 10 scans, and 10 scans for intermediates B, C, and C+DTT, respectively. The energy calibration and the average of the scans were conducted with EXAFSPAK.[7] Background subtraction and normalization of the averaged data were performed using PySpline.[8] The data were processed by fitting a second-order polynomial to the pre-edge region and subtracting this from the entire spectrum as a background. A three-region polynomial spline of orders 2, 3, and 3 was used to model the smoothly decaying post-edge region. The data were normalized by scaling the spline function to an edge jump of 1.0 at 9000 eV.

Comparison of the XANES data of intermediates B and C showed a lower energy resolution in the data of intermediate C. We note that the XAS data of intermediates B and C were not measured at the same beam time. The lower energy resolution in the data of intermediate C is considered to arise from the M_0 mirror not being fully optimized since comparison of the XANES data of a Cu foil, which were simultaneously measured with intermediates B and C for the energy calibration, also showed a lower energy resolution in the foil data measured with intermediate C (Fig.S11). For the comparison of the data having the different energy resolutions, one can try to improve the lower resolution data by deconvolving with an appropriate broadening function. However, this is numerically unstable and needs accurate knowledge of a broadening function. Thus, in this study, a simpler procedure of broadening the higher resolution data for intermediate B was attempted. A proper full width at half maximum (FWHM) value of a broadening function was determined from the least-squares minimization of the difference between the lower resolution Cu foil XANES data and the broadened higher resolution Cu foil XANES data which were calculated by convolving with Gaussian and Lorentzian broadening functions having an iteratively varied FWHM value. This least-squares minimization, which was performed using the LMFIT package in Python,[9] showed that the FWHM values of 1.83 and 0.38 eV for the Gaussian and Lorentzian broadening functions, respectively, were necessary to broaden the higher resolution Cu foil XANES data to the equivalent lower resolution data. These broadening functions with the proper FWHM are then convolved with the higher resolution XANES data of intermediate B to generate the equivalent lower resolution data for quantitative comparison (Fig.S9). We note that the energy resolution difference in the data had no effect on the EXAFS data.

The EXAFS curve fitting analysis program OPT in EXAFSPAK was used to fit the EXAFS data.[7] The theoretical phase and amplitude functions were calculated by FEFF (version 7.0).[10–13] Preliminary EXAFS fit parameters were obtained using structural models based on the DFT optimized structures. Then, the more accurate theoretical phase and amplitude functions were generated from the improved structural models. During the fitting process, the bond distance (R) and the mean-square thermal and static deviation in R (σ^2), which is related to the Debye–Waller factor, were allowed to vary. The non-structural parameter ΔE_0 (E_0 is the threshold energy where k is 0) was also allowed to vary but was constrained as a common value for all components in a

given fit. The amplitude reduction factor (S_0^2) was fixed to a value of 1.0. The coordination numbers were systematically varied in the course of the fit but were fixed within a given fit.

Bond valence sum (BVS) analysis was performed to further evaluate the best EXAFS fit.[14–17] In BVS, the sum of bond valences (V) is the oxidation state of the metal ion and is calculated using equation S3 where s_i is the individual bond valence for the bond between the metal ion and ligand i, r_i is the observed bond distance between the metal ion and ligand i (e.g., the bond distance from EXAFS), and r_0 and B are the empirically determined parameters.

$$V = \sum_i s_i = \sum_i \exp\left(\frac{r_0 - r_i}{B}\right) \quad [S3]$$

In this study, B is set equal to a universal value of 0.37 Å and r_0 was calculated using equation S4.[14]

$$r_0 = r_c + A \times r_a + P - D - F \quad [S4]$$

In equation S4, r_c is the contribution to r_0 from the cation (1.895 Å for Cu), A is the empirical parameter (0.8 for transition metal ions), r_a is the contribution to r_0 from the anion (0.000 Å for O and 0.490 Å for S), and P, D, and F are corrections for the number of p, d, and f electrons, respectively, in the cation (P = 0.035 Å and F = 0.000 Å for Cu, and D = 0.380 Å for d^{10} systems). This gives the r_0 values of 1.550 and 1.942 Å for the Cu–O and Cu–S bonds, respectively. Accordingly, the V values were calculated for the different EXAFS fits and are shown in Tables S2 and S3.

1.11. Density functional theory (DFT) calculations: Starting structures of FGE intermediates for the DFT optimizations performed in this study were generated in GaussView 6 (version 6.0.16) either from the published crystallographic structures, PDB: 6S07 and PDB: 6XTQ,[18,19] or from the related QM/MM-optimized structures previously reported.[20] The active site models in this study include the Cu atom, its first-coordination cysteine residues and the bound cysteine-containing peptide (which was truncated up to a tripeptide), the O_2 co-substrate, as well as key second-sphere residues (Trp239 and Ser274, and in some models Arg276) and selected active-site water molecules. Protein residues were truncated at either their C_α-atom or a side-chain C-atom, which was frozen in space during optimization. Similarly, the C_α-atoms of the bound peptide were frozen in space (to maintain its overall crystallographic conformation when bound to FGE) during optimization. DFT optimizations were performed in Gaussian (G16RevB.01), using the B3LYP functional and the def2SVP basis sets, using the GD3 dispersion and a PCM model with dielectric constant of $\varepsilon = 4.0$ and OFac = 0.8, RMin = 0.5.

1.12. Time-dependent density functional theory (TD-DFT) calculations & spectral simulations: TD-DFT calculations were employed on the DFT-optimized structures from this study (structures M5-7; as described in SI Methods 1.11) and the QM/MM-optimized structures reported previously (structures M1-4).[20] TD-DFT calculations were performed in Gaussian

(G16RevB.01) using the cam-B3LYP functional and the def2TZVP basis set, with N = 60 states and $\varepsilon = 4.0$. Visualization of the molecular orbitals (MOs) associated with TD-DFT transitions was done using Chemcraft (version 1.8, build 638m). The calculated absorption spectra were simulated as the sum of gaussian curves from the TD-DFT transitions using their respective calculated energies (in cm^{-1}) and oscillator strength (f_{calc}) intensities, and the default $\sigma = 0.4$ eV = 3,226 cm^{-1}. The simulated absorption spectra were calculated using equation S5 below, as described in the Gaussian manual (https://gaussian.com/uvvisplot/).

$$\varepsilon(\tilde{\nu}) = \sum_{i=1}^{n} \varepsilon_i(\tilde{\nu}) = \sum_{i=1}^{n} \left(1.3062974 \times 10^8 \cdot \frac{f_i}{\sigma} exp\left[-\left(\frac{\tilde{\nu} - \nu_i}{\sigma}\right)^2\right]\right)_i \qquad [S5]$$

2. Supplementary Figures & Analysis:

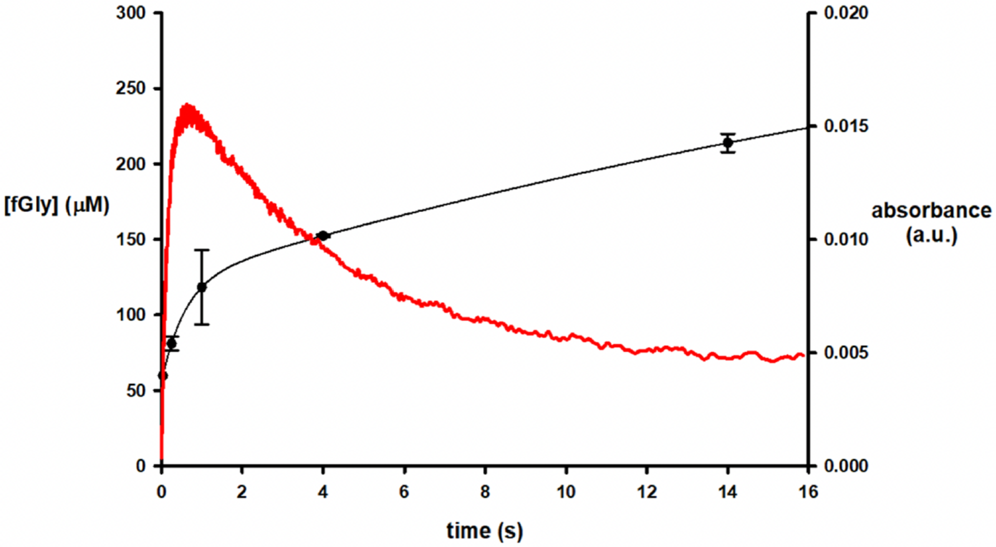

Figure S1. Product formation kinetics measured by rapid chemical quench. The anaerobic pre-formed FGE/Cu(I)/peptide complex (ES; 0.375 mM, post-mix concentration) was combined with O_2-saturated buffer (~1.1 mM, post-mix concentration) using a rapid-mixing instrument at 4 °C, pH 9.0. Time-point samples from 36 ms to 14 s were quenched by ejection into a vial containing HCl and the amount of produced formylglycine (fGly) was quantified by HPLC. Relative product formation was estimated by integration of the baseline resolved cysteine and formylglycine peptide peaks, using absorbance PDA detection at 280 nm, representing the absorption of the C-terminal tyrosine residue. Formation of fGly (black circles) overlayed with the SF-Abs kinetic trace at 420 nm (red trace) collected during parallel time courses indicates that the decay of intermediate A (and concomitant formation of intermediate B) are associated with product formation (either the final fGly product or its thioaldehyde precursor which would immediately hydrolyze to fGly under the acidic quenching conditions). Unlike the SF-Abs kinetics experiments where $[O_2]_{t=0}$ is in large excess (x10-fold) over $[ES]_{t=0}$ (0.1 mM, post-mix concentration), the relatively small (x3-fold) excess of $[O_2]_{t=0}$ over $[ES]_{t=0}$ appears to result in product formation rates that deviate from first-order kinetics (likely due to the effect of the earlier O_2 binding equilibria; Scheme S2) and explains the incomplete product formation (black circles) relative to the complete absorbance decay of the SF-Abs trace (in red) at late times (~15 s). The product formation data were fitted to a double-exponential model (black trace), and errors shown are the standard deviation of two or more replicates.

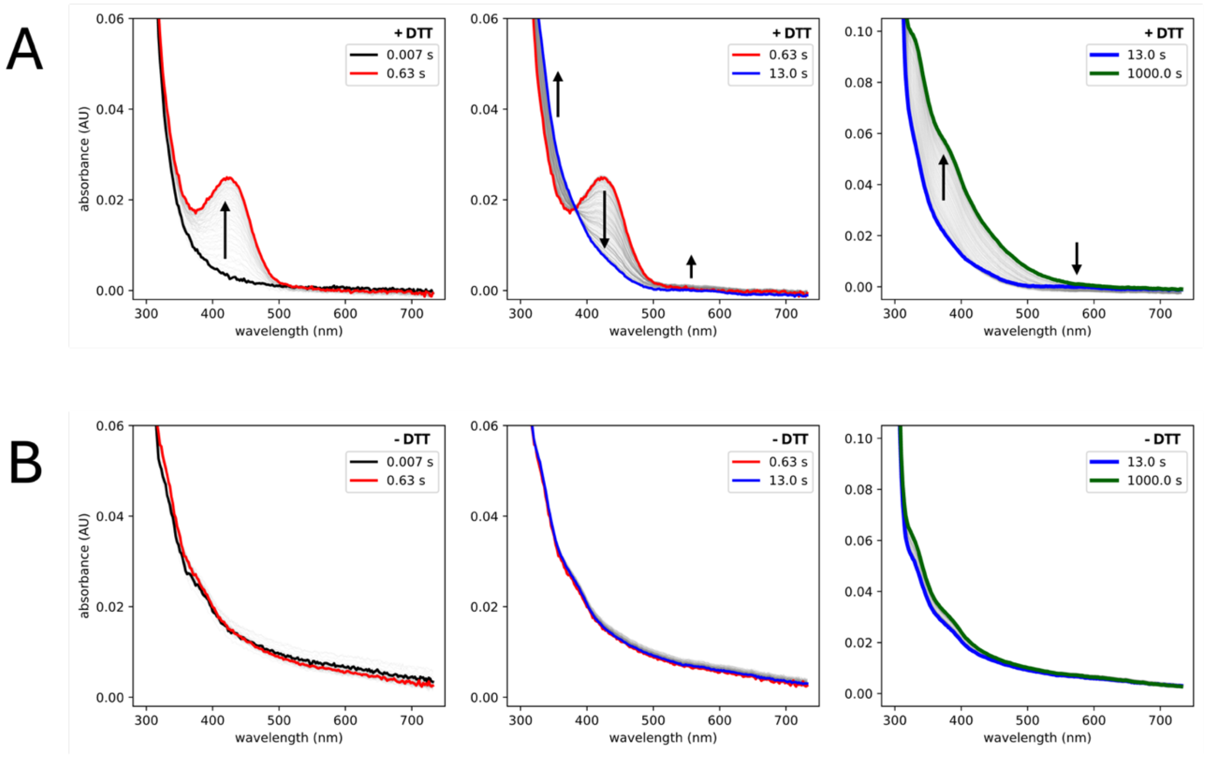

Figure S2. **Reaction of intermediate C (with or without prior DTT treatment) with O_2.** The post-mixing solution from the stopped-flow reaction of FGE/Cu(I)/peptide (ES; 0.1 mM, final concentration) with O_2 (~1.1 mM, final concentration; shown in Fig.2A-C in main text) was collected to evaluate whether the isolated form of the enzyme (with or without subsequent DTT treatment) can oxidize another equivalent of peptide substrate upon reaction with O_2 In short, the collected stopped-flow reaction product (i.e., the final stable intermediate C) was split in two solutions: the first solution was treated with 0.05 mM of DTT, while the second solution was not treated with DTT (control solution). Next both solutions were transferred inside the glove box and (i) were buffer-exchanged (to remove the aldehyde product, other reaction products, and the byproducts from DTT treatment), (ii) mixed with another equivalent (0.1 mM) of substrate peptide to form the equivalent ES complex, and (iii) were reacted with another solution of O_2 (~1.1 mM, final concentration) in the stopped-flow instrument. The SF-Abs spectra for these two reactions (A) with prior DTT treatment and (B) without DTT treatment (control) are shown for the same timescales as in the original reaction (Fig.2A-C in the main text). These results show that intermediate C must be reduced by $2e^-$ and supplied with another peptide substrate equivalent to react anew with O_2, in which case, the reaction proceeds to generate the same chromophoric intermediates as in the first single-turnover reaction. Note that treatment with >0.5 equivalents of DTT resulted in significant background reactions from DTT with O_2. Therefore, in Fig.S2A only 0.5 equivalents (relative to enzyme) of DTT were added leading to the 2e-reduction of only half the amount of intermediate C in solution (notice that the absorption in Fig.S2A is about half of that in Fig.2A-C of the main text). Note also that the lack of clearly resolved isosbestic points for the lower-energy bands in Fig.S2A (middle and right panels) is due to the lower amount ES concentration (due to use of 0.5 equivalents of DTT) and the effect of a small baseline drift in this dataset.

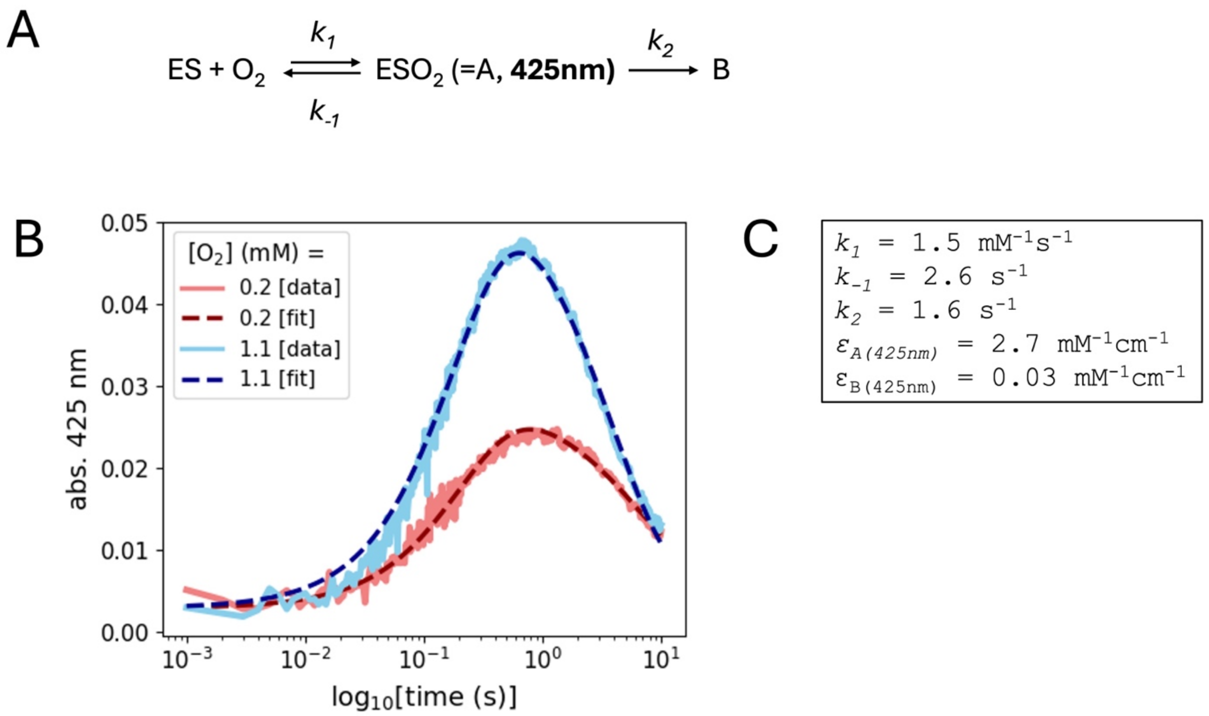

Figure S3. Kinetic fits for intermediate A assigned to the Cu(II)-$O_2^{\bullet -}$ (ESO_2) species. (A) Kinetic scheme where the chromophoric intermediate A is formed directly from the binding of O_2 to the FGE/Cu(I)/peptide (ES) complex and is assigned as the ESO_2 species. (B) Kinetic fits of the SF-Abs data from the reaction of FGE/Cu(I)/peptide (ES; 0.1 mM, post-mix) with O_2 at low (0.2 mM, post-mixing; in red) and high (1.1 mM, post-mixing; in blue) concentrations using the kinetic model shown in panel A. (C) The fitted kinetic parameters describe unexpectedly slow O_2 binding and dissociation rates to the ES complex (k_1 = 1.5 mM^{-1}s^{-1}, k_{-1} = 2.6 s^{-1}).[21]

A

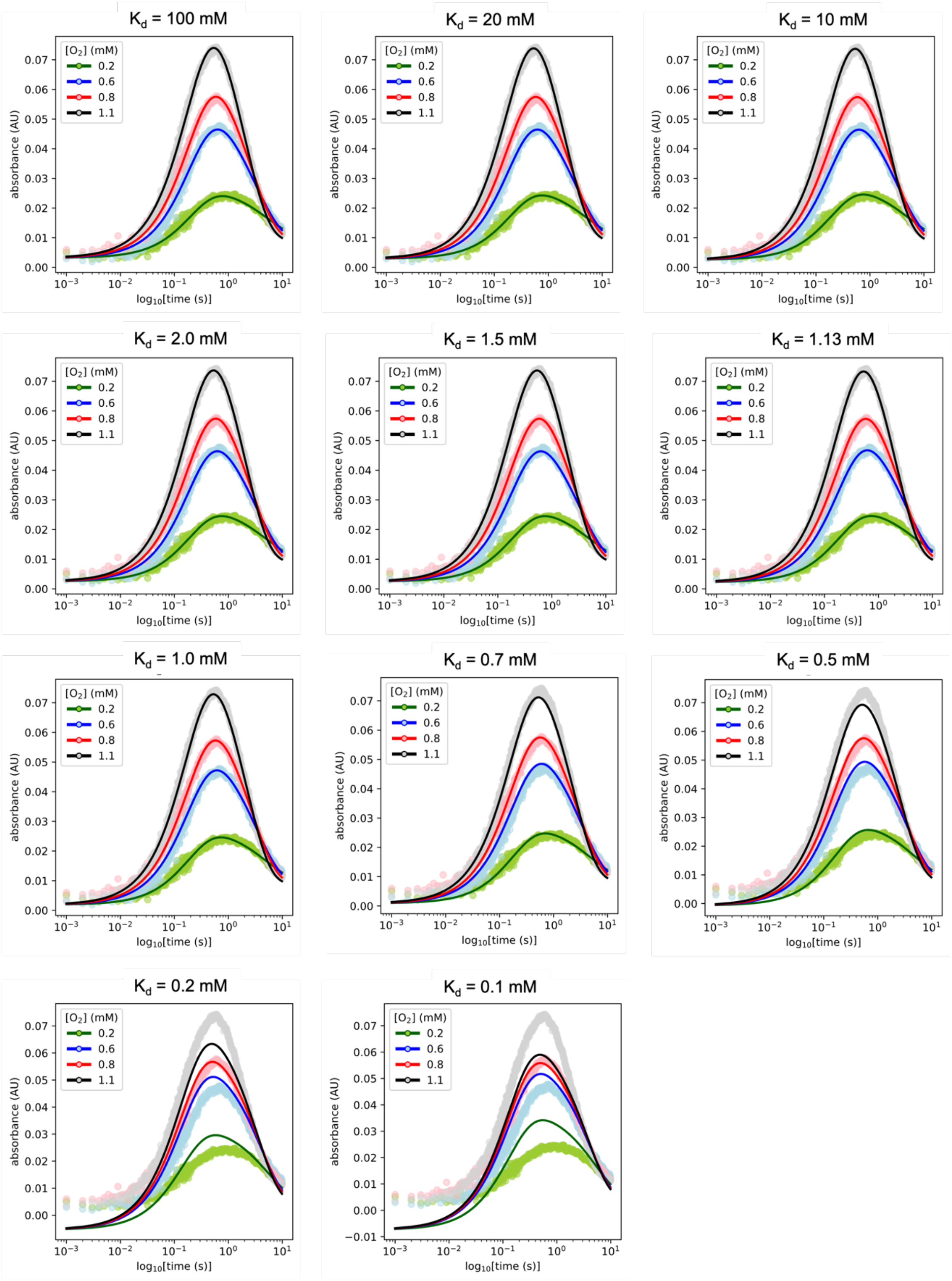
K_d = 100 mM
K_d = 20 mM
K_d = 10 mM
K_d = 2.0 mM
K_d = 1.5 mM
K_d = 1.13 mM
K_d = 1.0 mM
K_d = 0.7 mM
K_d = 0.5 mM
K_d = 0.2 mM
K_d = 0.1 mM
$[O_2]$ (mM)
0.2
0.6
0.8
1.1
absorbance (AU)
log_{10}[time (s)]

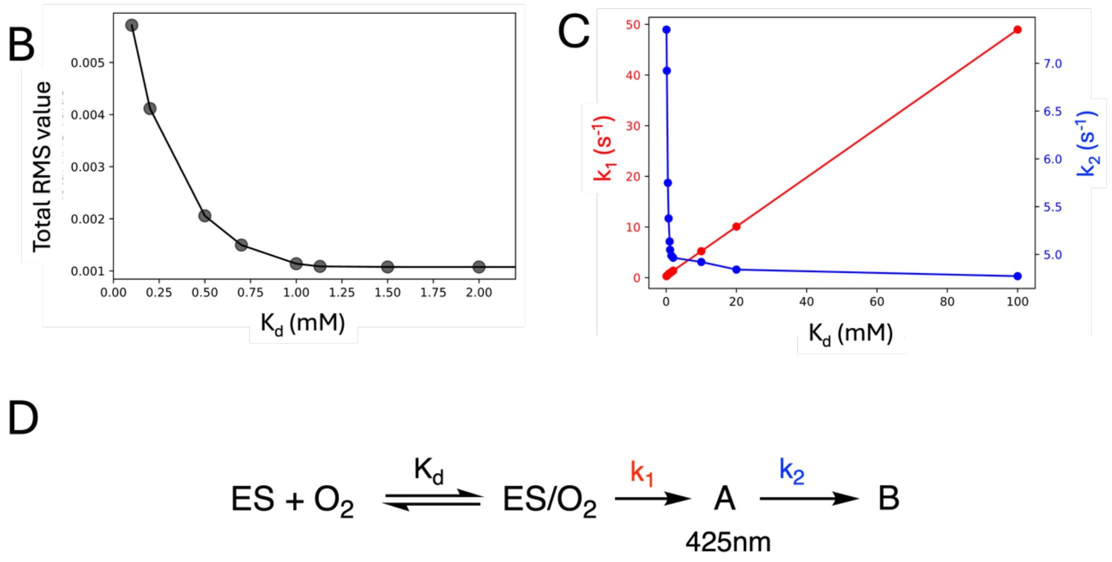

Figure S4. **Determining the O_2 binding constant (K_d) range for the FGE/Cu(I)/peptide (ES) complex.** (A) Simultaneous fitting of the 425 nm traces (intermediate A) at increasing O_2 concentrations and across a range of fixed values for the associated K_d using the kinetic scheme shown in panel D. (B) The total root-mean-square (RMS) deviations of the fits with different K_d values shown in panel A. The goodness of fit deteriorates significantly at $K_d < 1.0$ mM, which can also be clearly observed across the corresponding plots in panel A. (C) Dependence of the fitted values for the k_1 (red) and k_2 (blue) rate constants across different fixed values for K_d, indicating that different values of K_d would result in a different rate-limiting step in the FGE reaction. (D) The kinetic scheme employed to fit the SF-Abs data. (A-D) Overall, the analysis summarized in this figure places a lower limit on the value of K_d (based on goodness of fit) and, along with the crystallographically determined upper limit (i.e., the crystallographic ES/O_2 intermediate shows full occupancy for the bound O_2 under super-saturated O_2 conditions, [O_2] = ~5 mM generated by chlorite dismutase, therefore K_d << 5.0 mM),[19] define the possible range for O_2 binding to form the ES/O_2 intermediate to be ~1.0 mM < K_d < ~2.0 mM. Within this range, the elementary reaction step associated with k_2 remains rate-limiting.

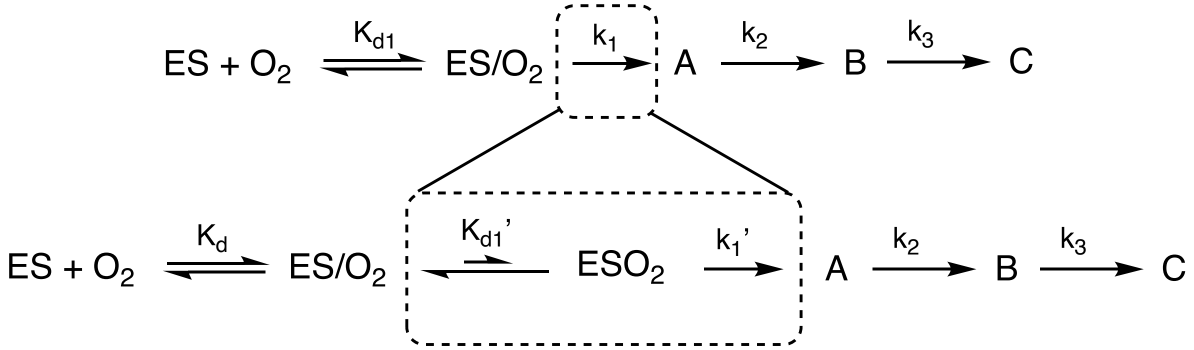

Scheme S2. Simplified & extended kinetic schemes. The simplified scheme (top) employed for the analysis of the SF-Abs data (see Fig.2 in the main text) can be extended to include the endergonic reactive species (ESO_2; presumed to be a Cu(II)-$O_2^{\bullet -}$ species capable of H-atom-abstraction from the bound substrate) that forms upon the fast and reversible coordination of the non-covalently bound O_2 in the ES/O_2 complex, as previously reported.[19] The k_1 value of the simplified model thus contains the elementary steps defined by K_{d1}' and k_1'. Since the reactive ESO_2 intermediate (or its ES/O_2 precursor) is not observed by SF-Abs kinetics, consistent with our expectation that this is a transient and/or high-energy intermediate, it is not possible to obtain the K_{d1}' and k_1' parameters from our SF-Abs data using the extended model. Instead, in our kinetic analysis for this study we employ the simplified model, since under the fast K_{d1}' regime, k_1' would share the same value as k_1.

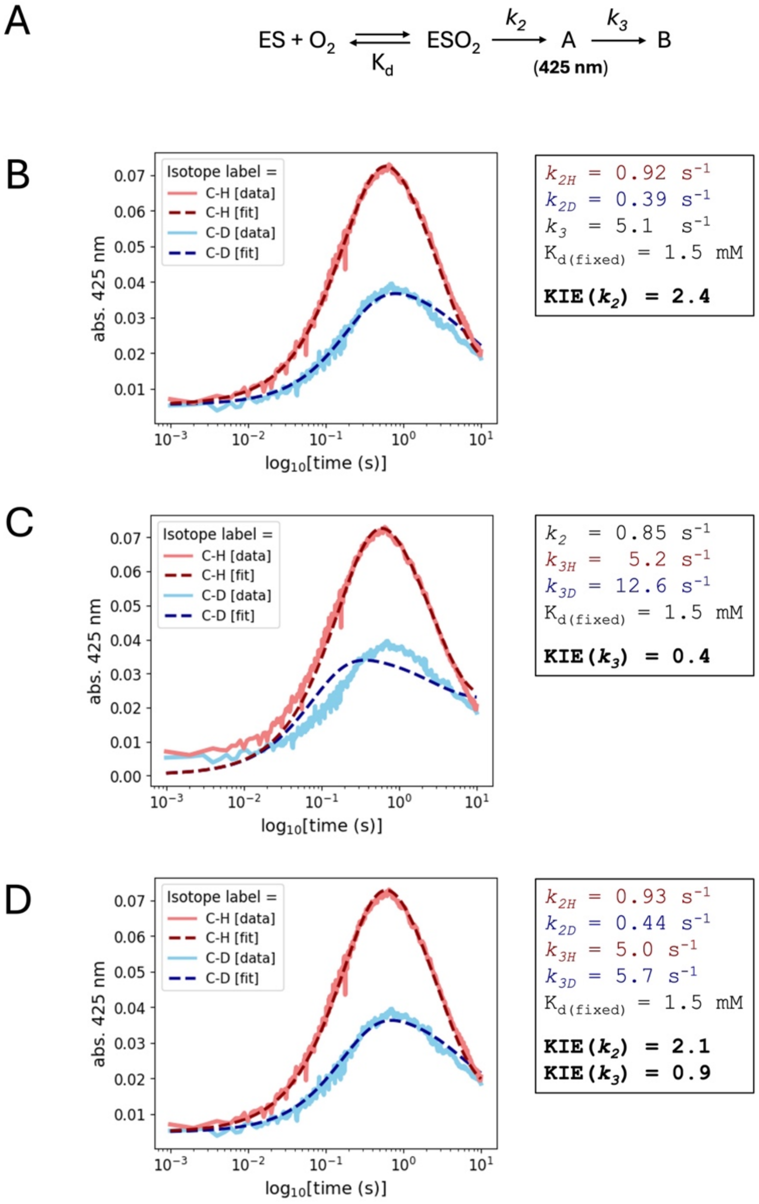

Figure S5. **Extended kinetic isotope effect (KIE) analysis.** (A) Kinetic scheme for the KIE analysis in COPASI. (B-D) SF-Abs data and fits for the reaction of FGE/Cu(I)/peptide (ES; 0.1 mM, post-mix) with O_2 (1.1 mM, post-mix). The ES complex was prepared either with the non-labeled substrate (C-H, in red) or the deuterated substrate (C-D in blue). For each case, the two traces for C-H/D are fit simultaneously with shared kinetic parameters (including a fixed value for K_d = 1.5 mM, which is within the acceptable range defined in Figure S4) except the rate constant(s) linked to a KIE: (B) k_2 only varies, (C) k_3 only varies, (D) both k_2 and k_3 vary. These results show that a primary KIE cannot be assigned on the decay of intermediate A (poor fit in panel C). On the other hand, the fits in panels B and D give similar results, indicating a large primary KIE on the formation of intermediate A. Note, that within the acceptable ~1.0 mM $< K_d <$ ~2.0 mM range, the primary remains on k_2 with values in the range 2 < KIE < 3.6 that are typical for the homolytic cleavage of C-H bonds.

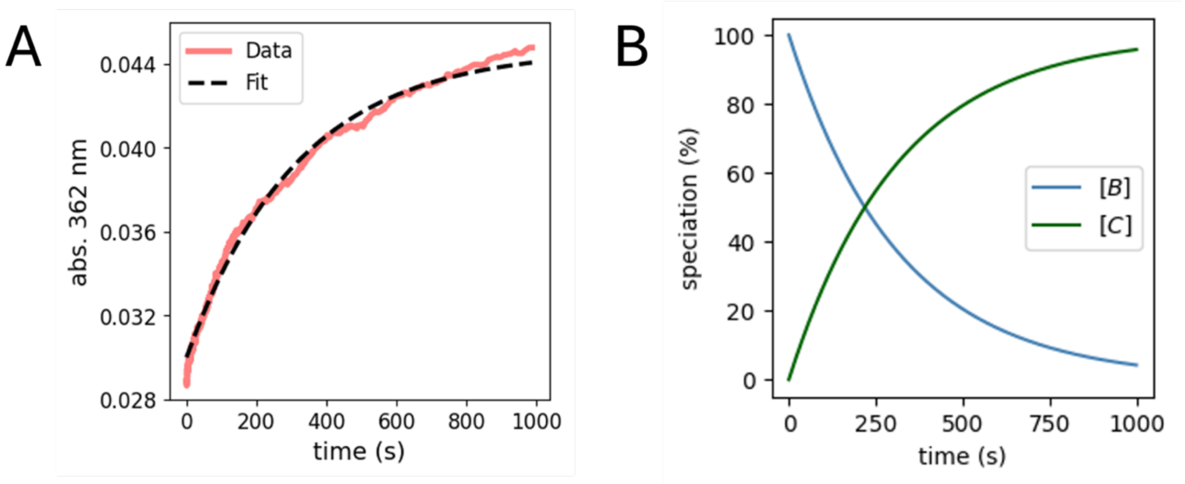

Figure S6. Kinetic fitting & time-course speciation of the conversion of intermediate B to C. (A) The 362 nm absorbance trace (solid red line) associated with the decay of intermediate B and the formation of intermediate C, and the kinetic fit (dashed black line) for the irreversible conversion of intermediate B to intermediate C. The kinetic fitting was performed in COPASI using the extinction coefficients shown in Table S1, with a fitted rate constant of 0.003 s^{-1}. To simplify the kinetic fitting (since the kinetic steps are effectively uncoupled) the initial time point was reset from 13 s (accumulation of intermediate B at 100% at that time) to 0 s. (B) The predicted time-course speciation for later reaction times (13-1000 s) based on the kinetic fitting from panel A (the corresponding early-time speciation plot is shown in Fig.2G in the main text).

Table S1. Summary of results from spectral analysis of intermediates A, B, and C. Their corresponding UV-Vis spectra are shown in Figure 3 A-C of the main text.[a]

	Intermediate A	Intermediate B	Intermediate C
**Peak1**			
mean (cm^{-1})	23,623	30,995	30,464
σ (cm^{-1})	1,659	1,802	1,509
ext. coeff. ($M^{-1}cm^{-1}$)	8,360	160	219
oscillator strength (f_{exp})	0.15	$3.1x10^{-3}$	$3.6x10^{-3}$
**Peak 2**			
mean (cm^{-1})	-	27,513	27,291
σ (cm^{-1})	-	2,772	3,020
ext. coeff. ($M^{-1}cm^{-1}$)	-	116	205
oscillator strength (f_{exp})	-	$3.5x10^{-3}$	$6.8x10^{-3}$
**Peak 3**			
mean (cm^{-1})	-	18,388	-
σ (cm^{-1})	-	1,887	-
ext. coeff. ($M^{-1}cm^{-1}$)	-	35	-
oscillator strength (f_{exp})	-	$7x10^{-4}$	-

[a] For details on spectral deconvolution, background corrections, Gaussian peak fitting, and f_{exp} estimation see SI Methods 1.8.

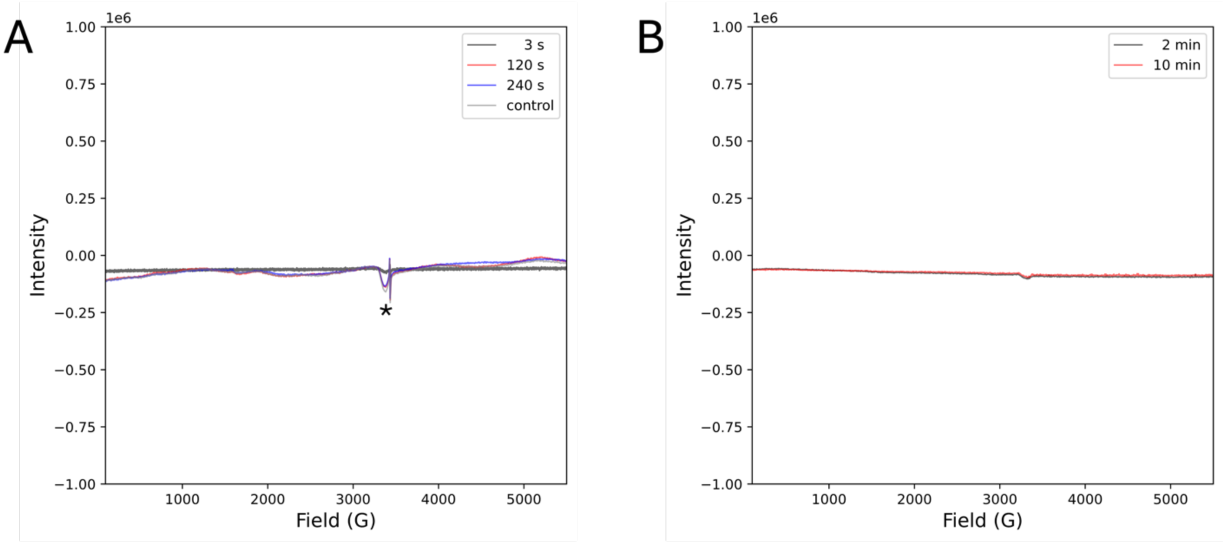

Figure S7. EPR spectra of intermediates B and C. (A) EPR spectra monitoring the formation and decay of intermediate B at different accumulations at 3 s (black spectrum, ~80% intermediate B during formation), 120 s (red spectrum, ~80% intermediate B during decay), and 240 s (blue spectrum, ~60% intermediate B during decay). The asterisk indicates an EPR artifact in this experiment also present in the buffer only control (grey spectrum). (B) EPR spectra monitoring the formation of intermediate C at different accumulations at 2 min (black spectrum, ~40% intermediate C, also consistent with blue spectrum in panel A) and 10 min (~ 80% intermediate C). (A-B) The lack of EPR features indicates that intermediates B and C contain EPR-silent species (note that a ~5% or higher % of Cu(II) signal would have been detected). The EPR spectra are collected for frozen rapid-freeze quench samples for different timepoints for the reaction of the anaerobic ES complex (Cu(I)-bound FGE in complex with the 14-mer peptide substrate, 0.5 mM final concentration) with an O_2-saturated solution (~0.5 mM final concentration) both in 50 mM Tris, 0.5 M NaCl, pH 9.0, 4 °C, in perpendicular mode, 13 dB, and 20K in panel A and 77K in panel B.

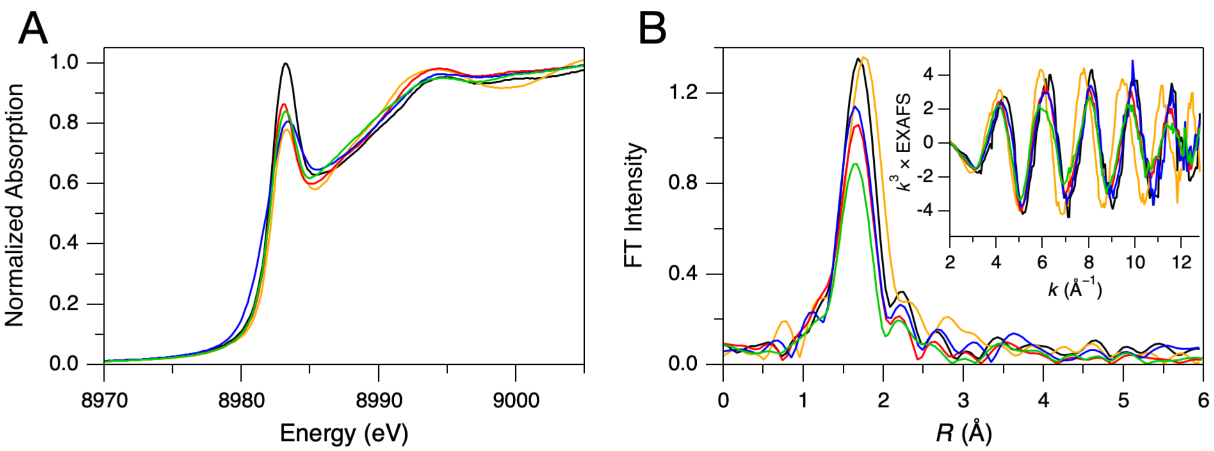

Figure S8. Comparison of XANES/EXAFS data. (A) Normalized Cu K-edge XANES spectra and (B) EXAFS data (inset) and their non-phase-shift-corrected Fourier transforms of intermediates B (red), C (blue), and C+DTT (green). The spectra of E (black) and ES (orange) are included for comparison.[1]

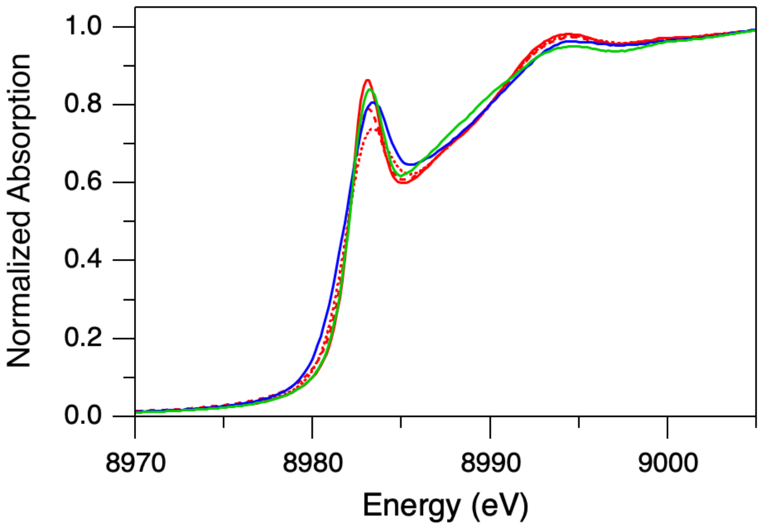

Figure S9. XANES spectral broadening for intermediate B. Normalized Cu K-edge XANES spectra of intermediates B (red), C (blue), and C+DTT (green). The spectra of intermediate B that were broadened by convolution with a Gaussian function (FWHM = 1.83 eV) or a Lorentzian function (FWHM = 0.38 eV) are shown with the dotted red and the dashed red lines, respectively. The normalized absorption amplitude of the Cu 1s→4p transition for intermediate B is ~0.86 (red spectrum in Fig.S9 and blue spectrum in Fig.3D), a value in-between that of E (~1.0) and ES (~0.77), indicating a two-coordinate Cu(I) having a weaker third ligand (a "2+1" site). The XANES data for intermediate C have the normalized absorption amplitude of ~0.81 for the Cu 1s→4p transition (blue spectrum in Fig.S9 and green spectrum in Fig.3D). Since the data for intermediate C were taken under lower energy resolution conditions, the higher resolution spectrum of intermediate B was convolved with Gaussian and Lorentzian broadening functions for a quantitative comparison; this gave an amplitude of ~0.74–0.79 for intermediate B (dotted and dashed red spectra in Fig.S9; see SI Methods 1.10). These broadened spectra for intermediate B indicate that the normalized absorption amplitude of intermediate C is close to or slightly higher than that of intermediate B with equivalent energy resolutions. Thus, the Cu(I) site in intermediate C has a similar coordination geometry as that in intermediate B (i.e., two-coordinate Cu(I) having one additional longer, weaker Cu–ligand interaction; a "2+1" site).

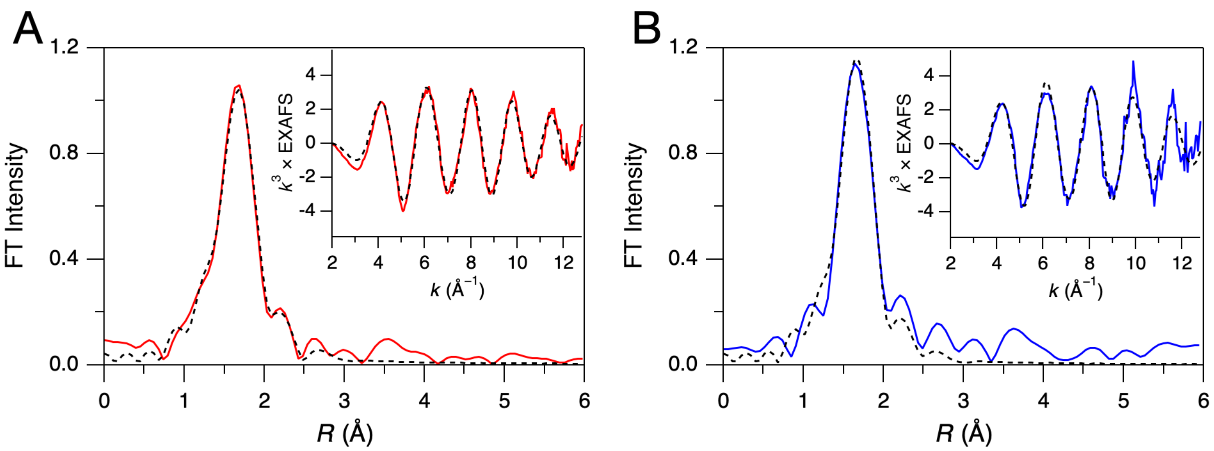

Figure S10. EXAFS data with best fits for intermediates B and C. Cu K-edge EXAFS data (inset) and their non-phase-shift-corrected Fourier transforms of (A) intermediates B (red) and (B) C (blue). The fits are shown with the dashed black lines and correspond to Fit B10 in Table S2 for intermediate B and Fit C10 in Table S3 for intermediate C.

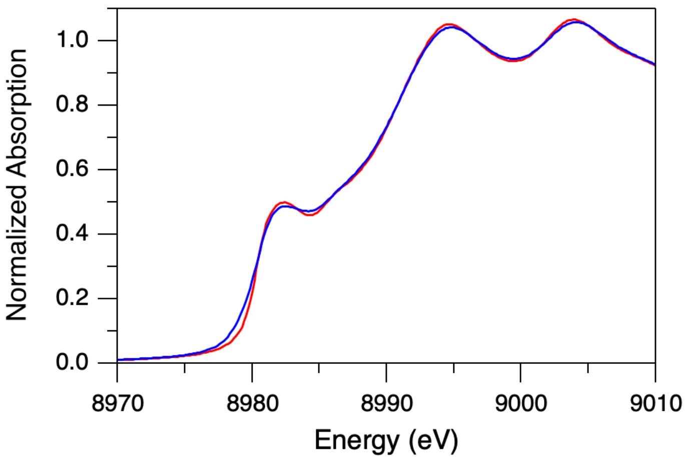

Figure S11. Normalized Cu K-edge XANES spectra of a Cu foil. The red and blue spectra are the foil data that were measured simultaneously with intermediates B and C, respectively (SI Methods 1.10). Note that the foil spectrum that was collected simultaneously with intermediate C+DTT is identical to the red spectrum.

Table S2. EXAFS fitting results and BVS values for intermediate B.

	CN/Path[a]	R (Å)[b]	σ^2 (Å^2)[c]	ΔE_0 (eV)	Error F[d]	V[e]
2-coordinate						
Fit B1	2 Cu–S	2.14	570	−21.59	0.231	1.17
Fit B2	1 Cu–S	2.17	272	−15.26	0.236	0.88
	1 Cu–O	1.95	408			
Fit B3	2 Cu–O	2.03	234	−1.77	0.321	0.55
3-coordinate						
Fit B4	3 Cu–S	2.14	863	−21.32	0.292	1.76
Fit B5	2 Cu–S	2.15	677	−17.47	0.240	1.41
	1 Cu–O	2.03	607			
Fit B6	1 Cu–S	2.17	433	−9.72	0.230	1.12
	2 Cu–O	2.01	770			
Fit B7	3 Cu–O	2.03	483	−2.63	0.339	0.82
2+1-coordinate[f]						
Fit B8	2 Cu–S	2.14	570	−21.46	0.202	1.28
	1 Cu–S	2.76	1533			
Fit B9	2 Cu–S	2.15	563	−20.34	0.212	1.21
	1 Cu–O	2.56	781			
Fit B10	1 Cu–S	2.17	267	−15.52	0.196	0.98
	1 Cu–O	1.95	439			
	1 Cu–S	2.80	1613			
Fit B11	1 Cu–S	2.18	277	−14.43	0.202	0.92
	1 Cu–O	1.96	422			
	1 Cu–O	2.57	903			
Fit B12	2 Cu–O	2.03	236	−1.98	0.303	0.61
	1 Cu–S	2.94	2125			
Fit B13	2 Cu–O	2.03	234	−1.53	0.296	0.61
	1 Cu–O	2.58	2549			

[a] CN is coordination number. Errors in CN is ±25% and those in the identity of the scatterer Z are ±1.

[b] Estimated standard deviations in R are ±0.02 Å.

[c] The σ^2 values are multiplied by 10^5.

[d] Error F is given by $[\sum k^6(\chi_{experimental} - \chi_{calculated})^2/\sum k^6\chi_{experimental}^2]^{1/2}$.

[e] V is the sum of the bond valences.

[f] Fits under "*2+1-coordinate*" were obtained by including one longer metal–ligand interaction in 2-coordinate fits.

Table S3. EXAFS fitting results and BVS values for intermediate C.

	CN/Path[a]	R (Å)[b]	σ^2 (Å^2)[c]	ΔE_0 (eV)	Error F[d]	V[e]
2-coordinate						
Fit C1	2 Cu–S	2.13	532	−21.91	0.338	1.20
Fit C2	1 Cu–S	2.17	301	−13.34	0.330	0.88
	1 Cu–O	1.95	220			
Fit C3	2 Cu–O	2.01	205	−1.99	0.407	0.58
3-coordinate						
Fit C4	3 Cu–S	2.13	816	−21.32	0.403	1.80
Fit C5	2 Cu–S	2.13	574	−18.40	0.339	1.48
	1 Cu–O	2.03	729			
Fit C6	1 Cu–S	2.15	480	−8.63	0.338	1.15
	2 Cu–O	2.01	573			
Fit C7	3 Cu–O	2.01	437	−2.64	0.436	0.87
2+1-coordinate[f]						
Fit C8	2 Cu–S	2.13	533	−21.80	0.326	1.33
	1 Cu–S	2.71	1332			
Fit C9	2 Cu–S	2.13	529	−20.53	0.335	1.27
	1 Cu–O	2.55	590			
Fit C10	1 Cu–S	2.16	303	−13.54	0.310	1.00
	1 Cu–O	1.95	249			
	1 Cu–S	2.77	1628			
Fit C11	1 Cu–S	2.17	317	−11.98	0.313	0.93
	1 Cu–O	1.96	198			
	1 Cu–O	2.58	444			
Fit C12	2 Cu–O	2.01	206	−2.25	0.391	0.66
	1 Cu–S	2.88	2311			
Fit C13	2 Cu–O	2.02	207	−1.68	0.381	0.64
	1 Cu–O	2.51	2139			

[a] CN is coordination number. Errors in CN is ±25% and those in the identity of the scatterer Z are ±1.

[b] Estimated standard deviations in R are ±0.02 Å.

[c] The σ^2 values are multiplied by 10^5.

[d] Error F is given by $[\sum k^6(\chi_{\text{experimental}} - \chi_{\text{calculated}})^2/\sum k^6\chi_{\text{experimental}}^2]^{1/2}$.

[e] V is the sum of the bond valences.

[f] Fits under "*2+1-coordinate*" were obtained by including one longer metal–ligand interaction in 2-coordinate fits.

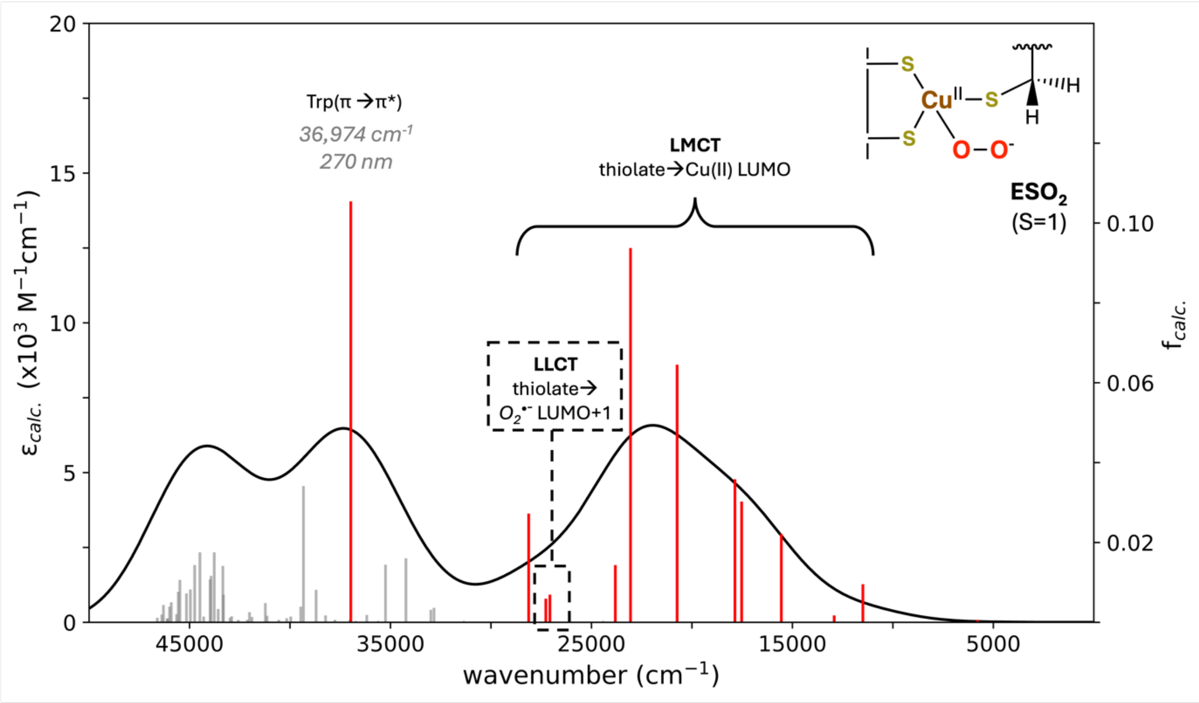

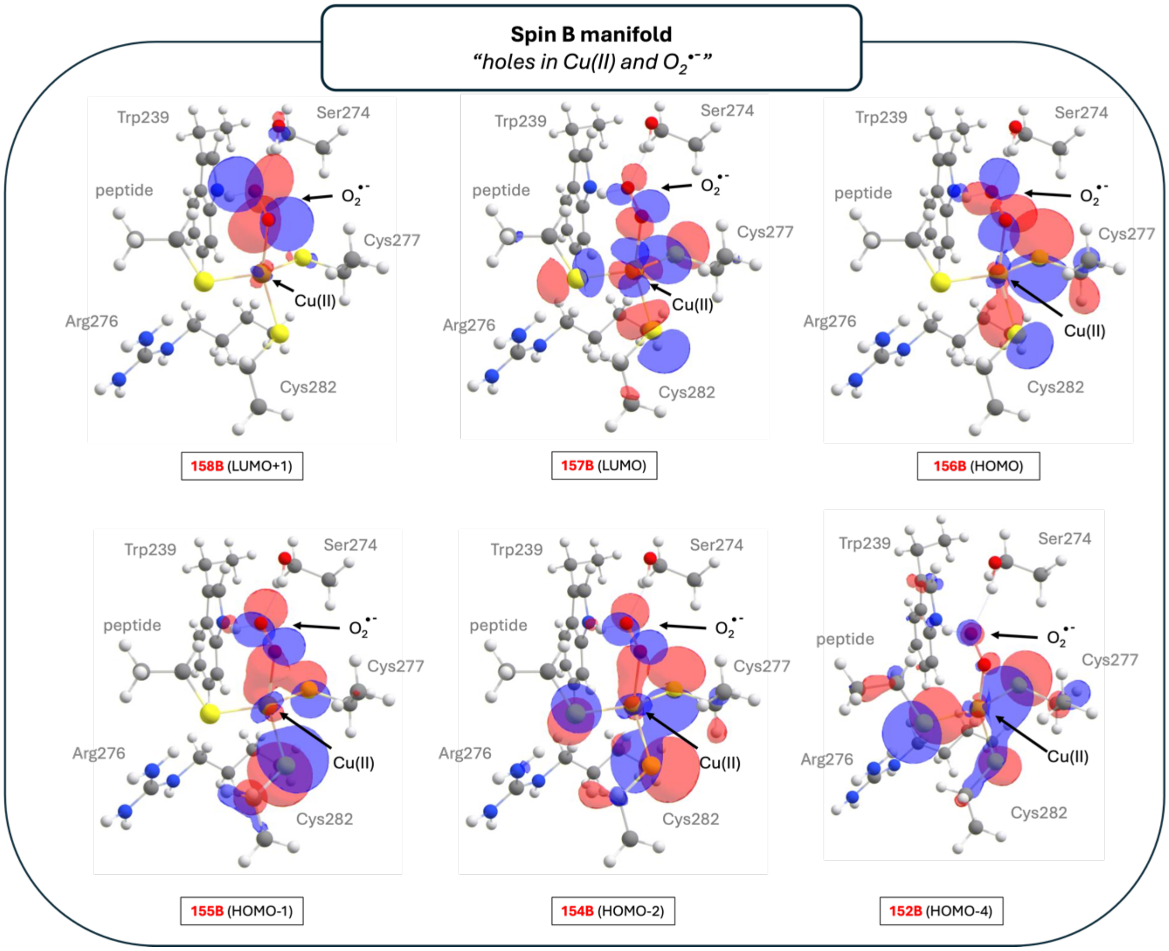

(**Fig.S12**, *continues to next page*)

Figure S12. **Extended TD-DFT analysis for the ESO_2 (S=1) species**. The calculated TD-DFT spectrum (cam-B3LYP/def2TZVP/N=60/ε=4.0) is shown in the top, with selected transitions highlighted (in red) of the Β spin manifold (with two holes in each of the Cu(II) and the superoxo ligand) including the lower-energy transitions from thiolate-centered MOs to the Cu(II) LUMO (LMCT) and to the superoxo LUMO+1 (LLCT; in dashed box), and the higher energy Trp $\pi \rightarrow \pi^*$ transition. The selected associated MOs are shown in the bottom. The DFT-optimized structure of ESO_2 (S=1; B3LYP/def2SVP/ε=4.0) is shown in Fig.S20B.

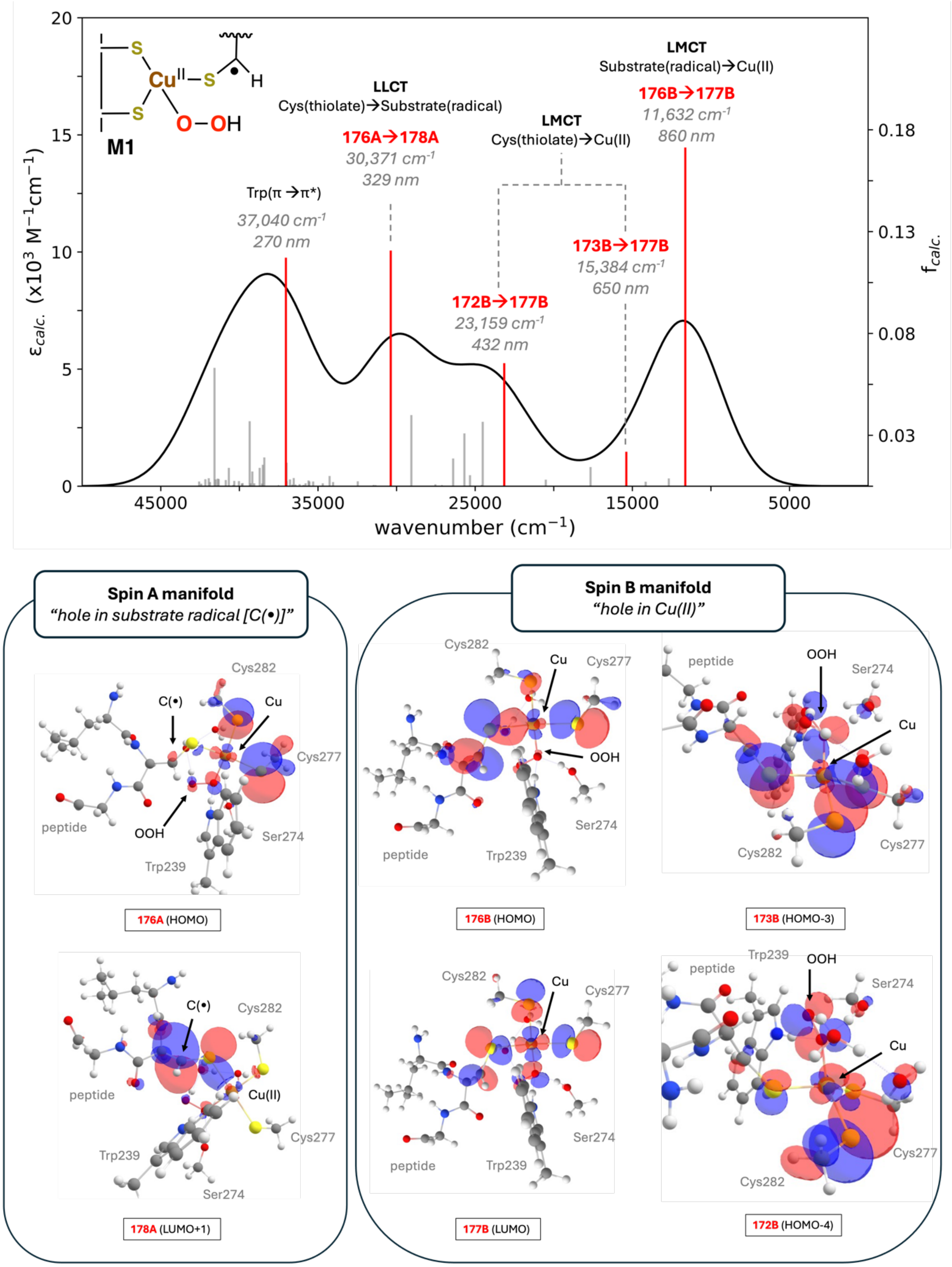

(**Fig.S13,** *continues to next page*)

Figure S13. **Extended TD-DFT analysis for the M1 species**. Selected transitions of the TD-DFT spectrum (*top;* cam-B3LYP/def2TZVP/N=60/ε=4.0) of M1 (S=0; using the reported DFT-optimized structure for this species)[20] are highlighted in red and are annotated with the numbers of their associated MOs (*bottom*). As shown above, the calculated TD-DFT spectrum of M1 contains several thiolate→Cu(II) and substrate(radical)→Cu(II) ligand-to-metal charge transfer (LMCT) and ligand-to-ligand CT (LLCT) transitions that would result in a highly chromophoric intermediate with several absorption bands in its UV-Vis region (35,000-15,000 cm^{-1}). This is inconsistent with the experimental absorption spectrum for intermediate A which exhibits a single absorption feature at 23,600 cm^{-1} (Fig.3A in main text). Thus M1 is excluded as intermediate A.

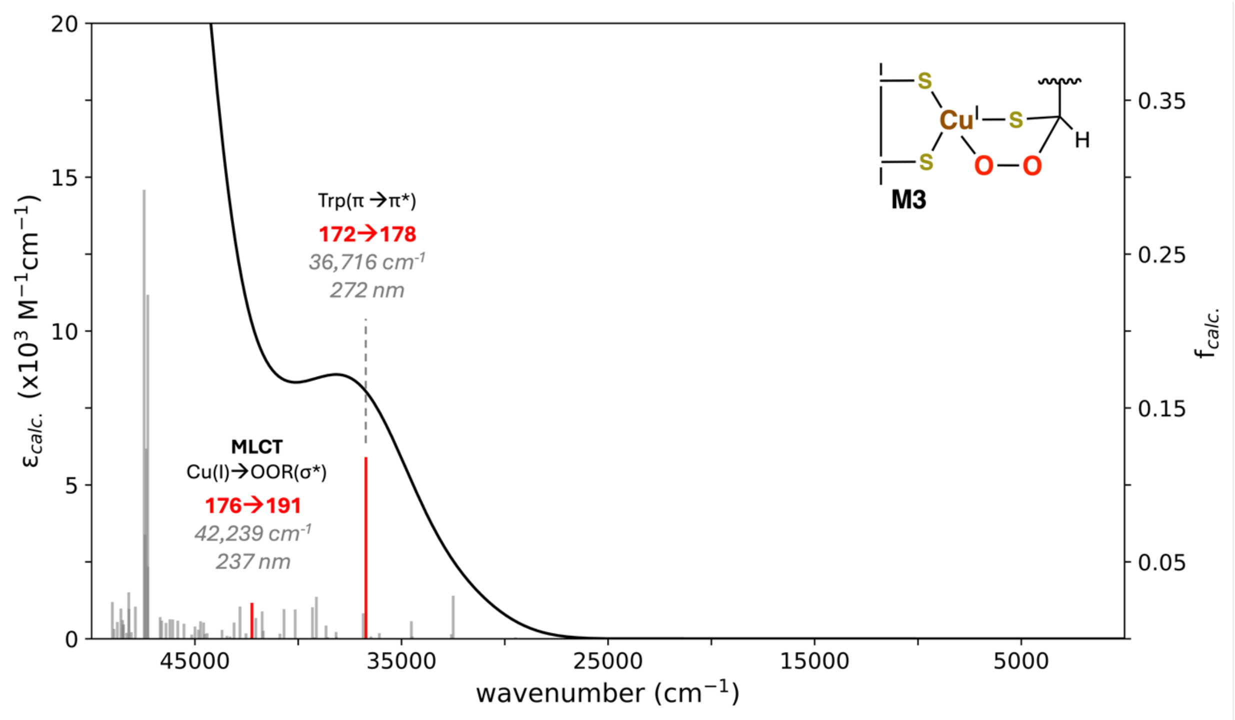

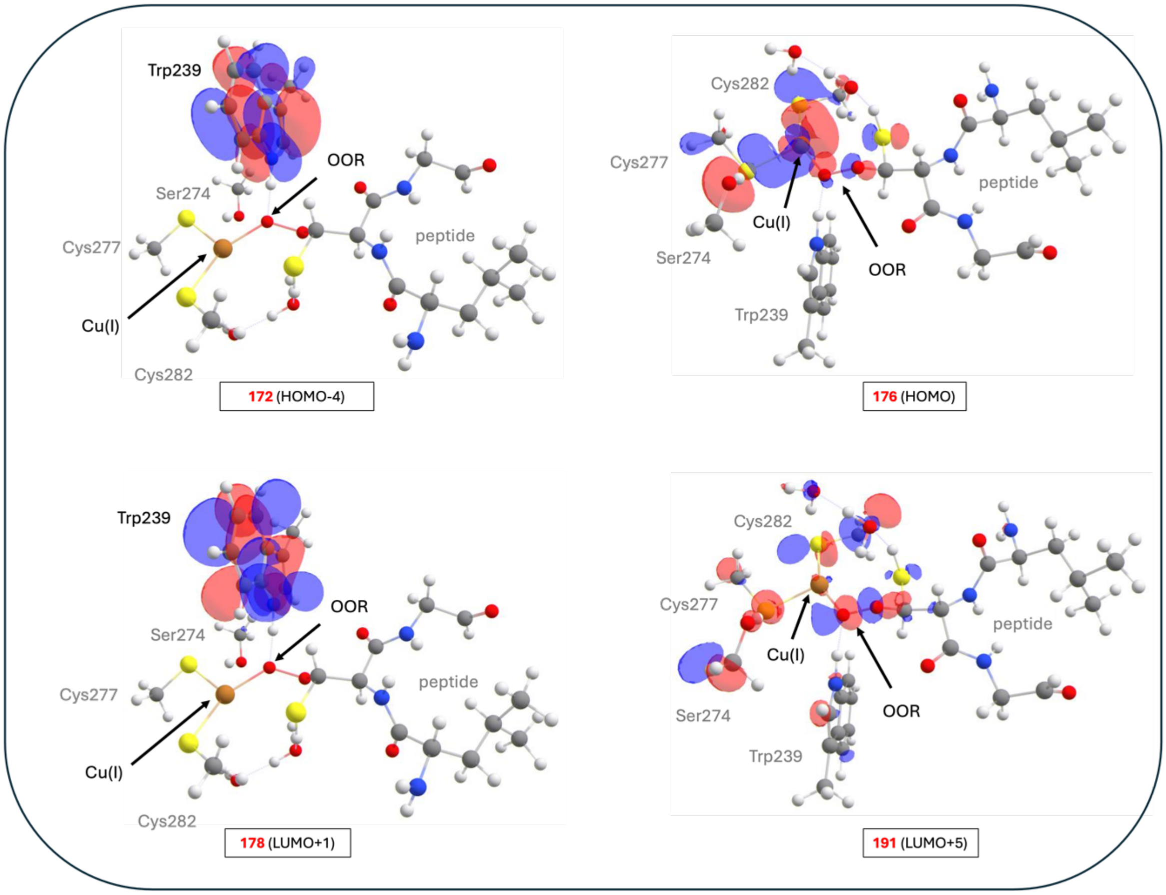

(**Fig.S14,** *continues to next page*)

Figure S14. Extended TD-DFT analysis for the M3 species. Selected transitions of the TD-DFT spectrum (*top*; cam-B3LYP/def2TZVP/N=60/ε=4.0) of M3 (S = 0) are highlighted in red and are annotated with the numbers of their associated MOs (*bottom*). This Cu(I)-OOR alkylperoxo species was proposed as an intermediate in the FGE mechanism by previous computational work.[20] The TD-DFT spectrum for M3 does not contain any transitions that would correspond to the 23,600 cm^{-1} of intermediate A (Fig.3A in main text). Notably, the Trp(π→π*) transition is present at 36,700 cm^{-1} (272nm; serving as an internal reference for the 280 nm protein absorption feature across the different computational models) and the Cu(I)→OOR(σ*) MLCT transition is at slightly higher energy (42,200 cm^{-1}). Thus, the Cu(I)-OOR (M3) species is excluded as intermediate A.

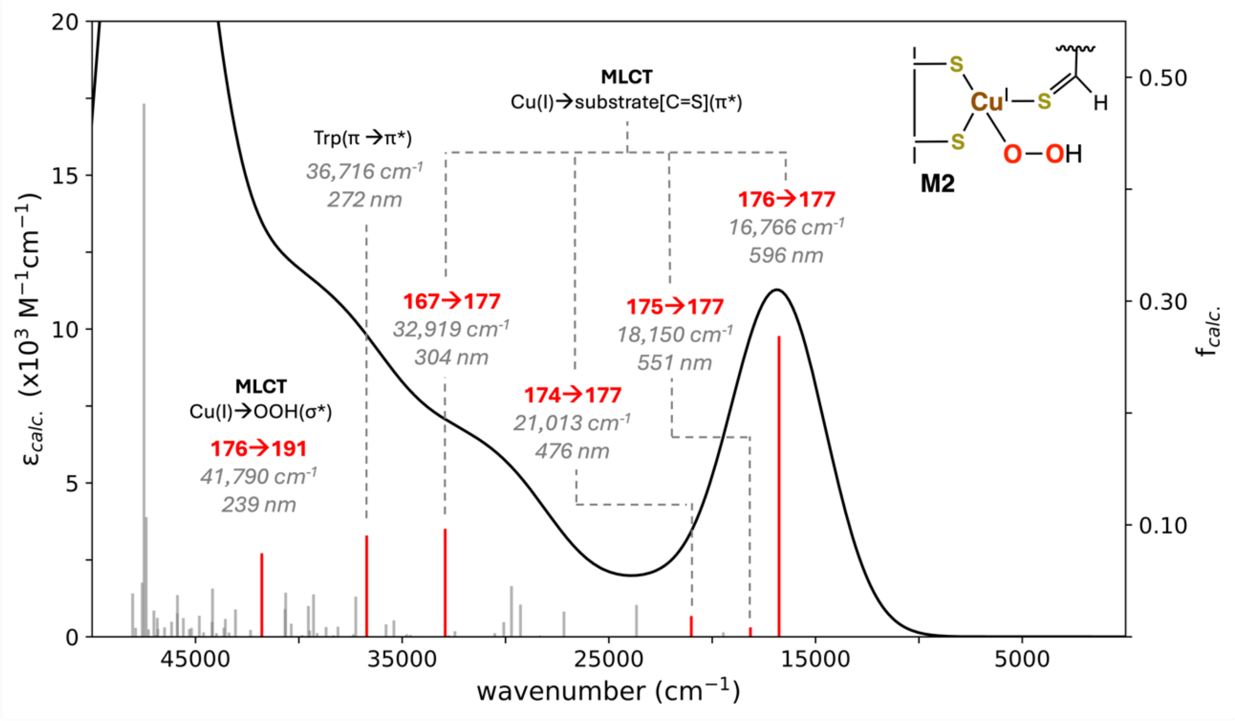

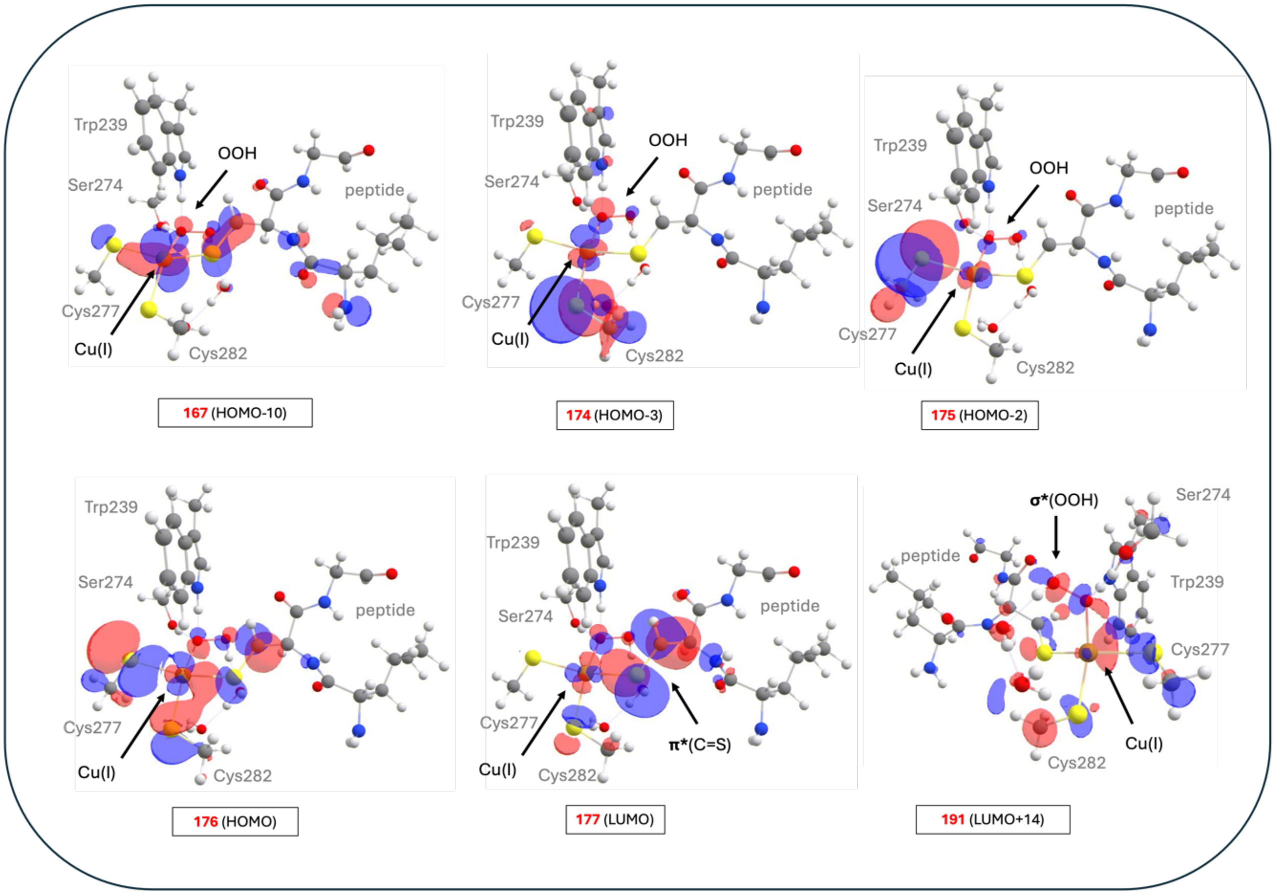

(**Fig.S15,** *continues to next page*)

Figure S15. Extended TD-DFT analysis for the M2 species. Selected transitions of the TD-DFT spectrum (*top*; cam-B3LYP/def2TZVP/N=60/ε=4.0) of M2 (S = 0) are highlighted in red and are annotated with the numbers of their associated MOs (*bottom*). Note that the TD-DFT spectrum of M2 exhibits a high-energy Cu(I)→OOH(σ*) MLCT transition at 41,790 cm^{-1} similar to the Cu(I)→OOR(σ*) MLCT transition in M3, both of which would be obscured by the intense 280 nm band from the FGE protein in our SF-Abs experiments. Therefore, the predicted UV-Vis absorption spectrum of M2 contains a single, lower-energy transition at 16,766 cm^{-1} and 12 x 10^3 $M^{-1}cm^{-1}$ intensity, that is in reasonable agreement with the experimental spectrum of intermediate A (23,600 cm^{-1} and 8 x 10^3 $M^{-1}cm^{-1}$; Fig.3A in the main text and Table S1). Note, that the underestimation of the energy of CT transitions by TD-DFT is consistent with previous studies (e.g. in equivalent approached on the ternary and peroxy-intermediates of the TPH enzyme)[22] which provide an energy calibration for our methodology.

Extended Analysis S1: ***Systematic correlation of model M3-6 to intermediate B.***
First, we consider the Cu(I)-OOR species (M3), which was previously excluded as intermediate A. While the 2S1O coordination of M3 is consistent with the EXAFS analysis for intermediate B (blue spectra in Fig.3E and Table S2), its TD-DFT spectrum (Fig.4B and Fig.S14) lacks a low-energy and weak-intensity feature that would correlate to the observed absorption band at 18,400 cm^{-1} for intermediate B (Fig.3B). Therefore, M3 is also excluded as intermediate B. Next, we consider the Cu(II)-oxyl species (M4 in Fig.4A and Fig.S16A) that was proposed to proceed M3 in previous computational work.[20] The +2 oxidation state and 3S1O coordination sphere of the Cu site are not consistent with the XANES data (blue spectrum in Fig.3D) and the EXAFS analysis (blue spectra in Fig.3E and Table S2), respectively. The calculated TD-DFT absorption spectrum for M4 (Fig.S16B) contains several absorption features with high intensities across the 35,000-15,000 cm^{-1} range, also inconsistent with the experimental spectrum for intermediate B (Fig.3B). Therefore, M4 is excluded as intermediate B. Dissociation of the thioaldehyde product from the assigned intermediate A (M2 in Fig.4A) would lead to another Cu(I)-OOH species (M5 in Fig.4A) that could correspond to intermediate B. While the +1 oxidation state and 2S1O coordination of the Cu site agree with the XANES data (blue spectrum in Fig.3D) and the EXAFS analysis (blue spectra in Fig.3E and Table S2), respectively, its TD-DFT spectrum (Fig.4B and Fig.S17) lacks the characteristic lower-energy and weak-intensity feature observed experimentally for intermediate B (Fig.3B). Therefore, M5 is also excluded as intermediate B. Finally, we consider a sulfenate-Cu(I) species coordinated by the thioaldehyde product (M6 in Fig.4A) that forms from the oxidation of one of the cysteine residues by the Cu(II)-OOH of intermediate A (M2). The +1 oxidation state and 2S1O coordination of the Cu site agree with the XANES data (blue spectrum in Fig.3D) and the EXAFS analysis (blue spectra in Fig.3E and Table S2), respectively, for intermediate B. The TD-DFT spectra of M6 exhibit a Cu(I)→ thioaldehyde(π*) MLCT transition at a similar energy (16,530 cm^{-1}; Fig.S18A) as the characteristic low-energy feature in the absorption data for intermediate B (Fig.3B). However, the TD-DFT-predicted intensity of this low-energy feature (ε_{calc} = 3 x 10^3 $M^{-1}cm^{-1}$) is much higher than its experimental value (ε_{exp} = 0.05 x 10^3 $M^{-1}cm^{-1}$). Thus, we proceeded to investigate whether this discrepancy could be explained by the strength of the thioaldehyde-Cu(I) bonding interaction in our structural model. To this end, we obtained the TD-DFT spectra for a series of DFT-optimized structures of M6 with its Cu(I)-S(thioaldehyde) bond systematically increased (Fig.S18B). As predicted, the calculated extinction coefficient for the lowest energy band (corresponding to the Cu(I)→thioaldehyde(π*) MLCT transition) decreases with increasing Cu(I)-S(thioaldehyde) bond length (Fig.S18B), and the TD-DFT spectrum of M6 with the longer Cu(I)-S(thioaldehyde) bond (3.00 Å; Fig.4B) is in reasonable agreement with the experimental UV-Vis absorption spectrum of intermediate B (Fig.3B). Upon further examination of the FGE active site, we identified Arg276 as a second-sphere residue that could interact with the ligand(s) of the Cu(I) site of M6 and result in the longer Cu(I)-S bond as revealed from the EXAFS analysis on intermediate B. Consistent with this hypothesis, the DFT-optimized structure of an expanded model of M6 that includes Arg276 exhibits a T-shape Cu(I) site with a weak Cu(I)-S(thioaldehyde) interaction (3.07 Å, Fig.S18C). Therefore, intermediate B is assigned as the Cu(I)-sulfenate species (M6) with a long Cu(I)-S(thioaldehyde) bonding interaction.

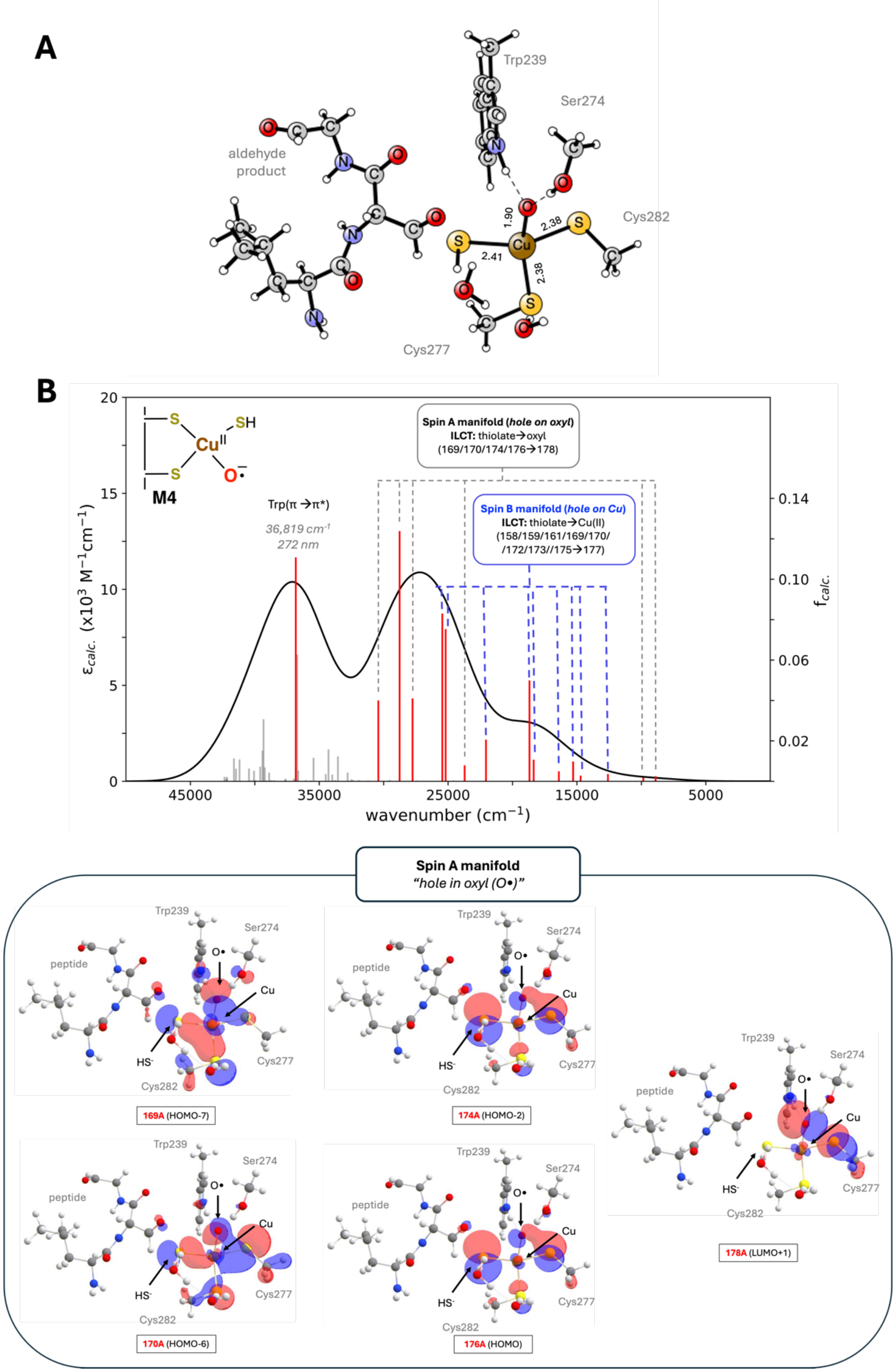

(**Fig.S16**, *continues to next page*)

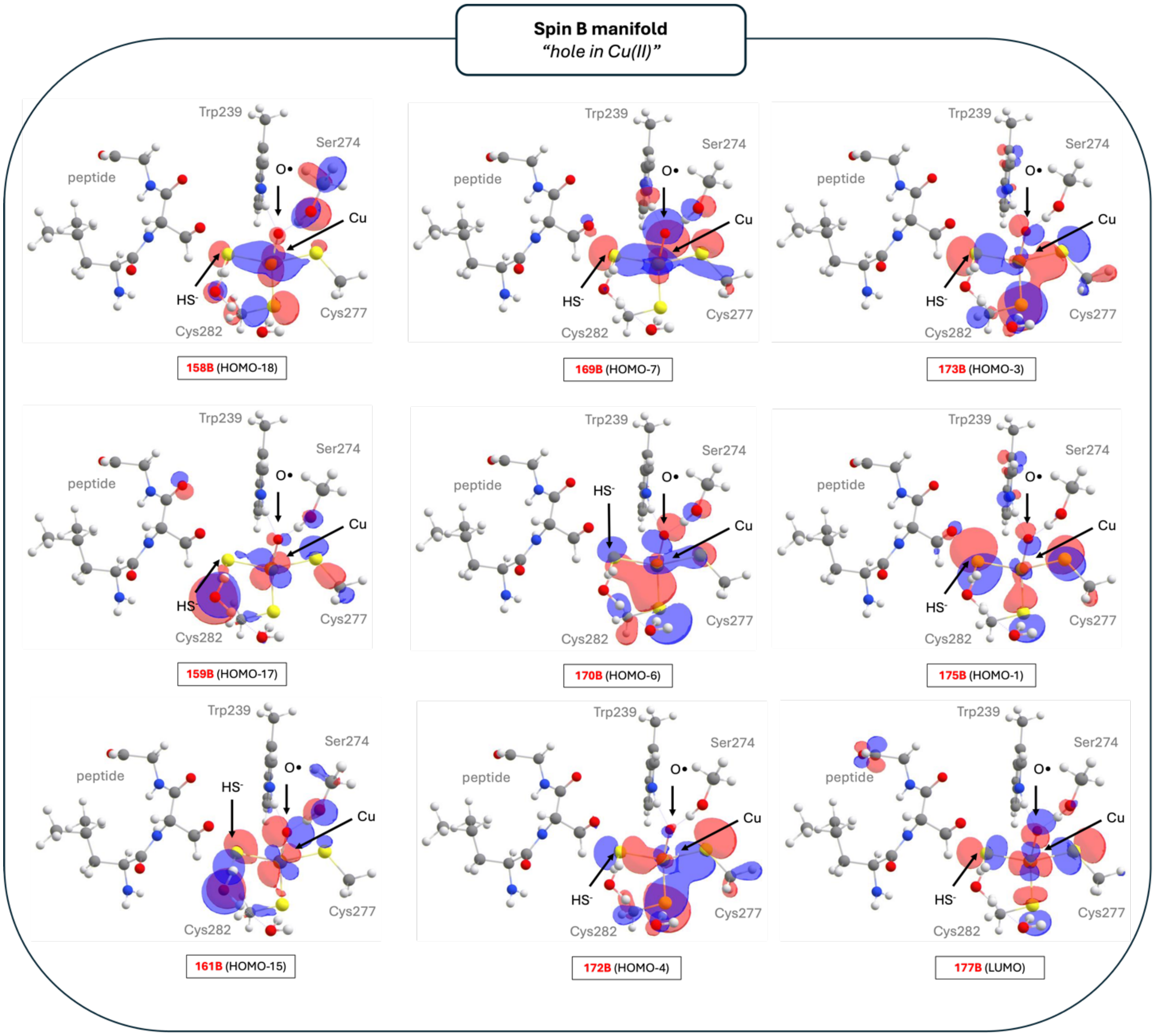

Figure S16. The DFT-optimized structure & extended TD-DFT analysis for the M4 species (A) The DFT-optimized structure of M4 (B3LYP/def2SVP/ε=4.0), obtained using the previously reported DFT-optimized structure,[20] as a starting structure to obtain the triplet electronic grounstate used for (B) the extended TD-DFT analysis (cam-B3LYP/def2TZVP/N=60/ε=4.0) for the M4 species, with the MOs from the spin A and B manifolds associated with the highlighted TD transitions (indicated with grey dashed lines for spin manifold A and blue dashed lines for B) shown in the corresponding boxes.

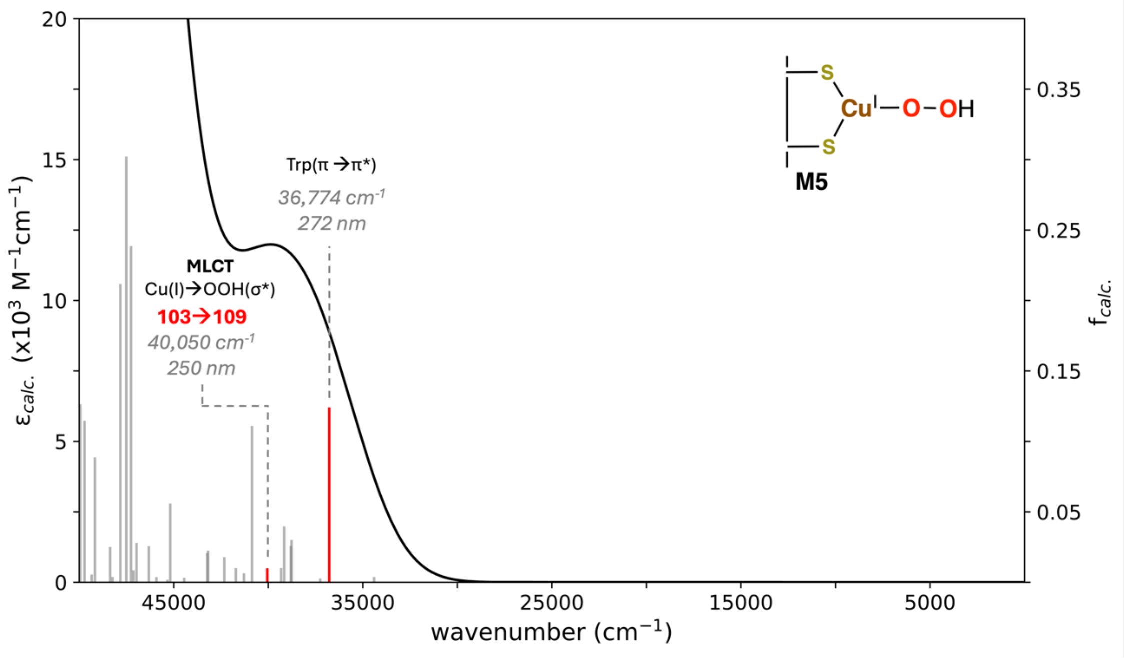

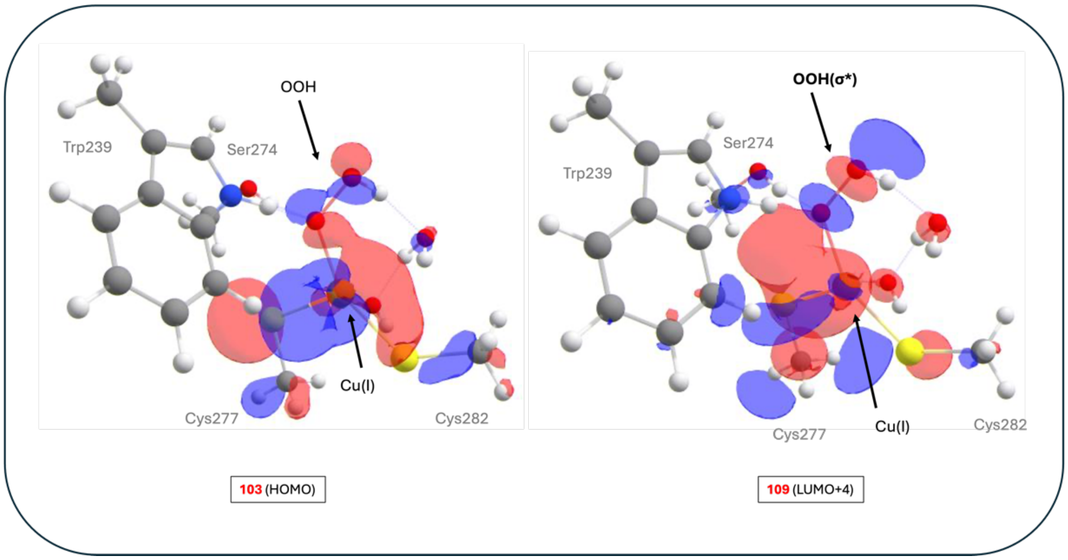

Figure S17. Extended TD-DFT analysis for the M5 species Selected transitions of the TD-DFT spectrum (*top*; cam-B3LYP/def2TZVP/N=60/ε=4.0) of M5 (S = 0) are highlighted in red and are annotated with the numbers of their associated MOs (*bottom*).

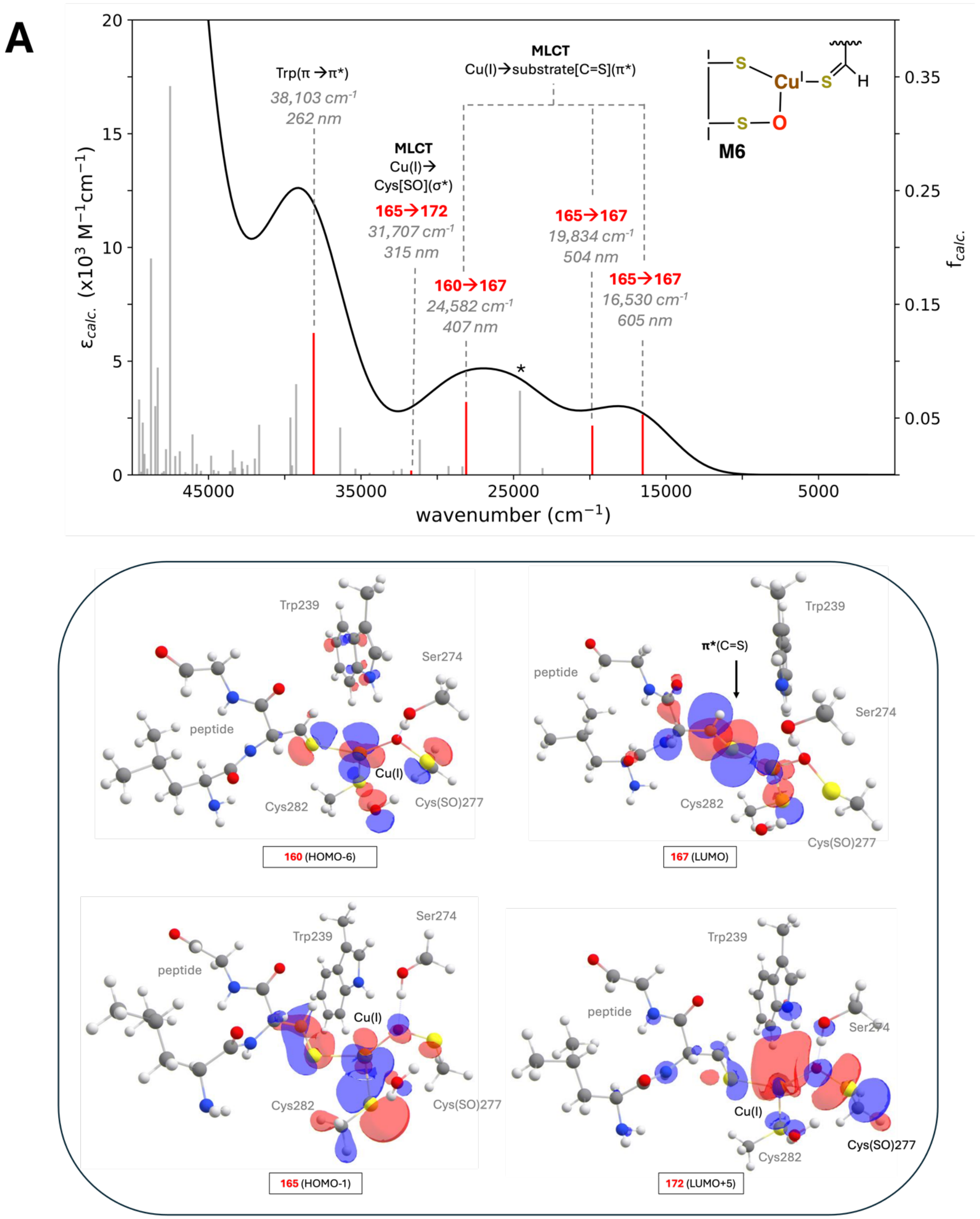

(**Fig.S18,** *continues to next page*)

B

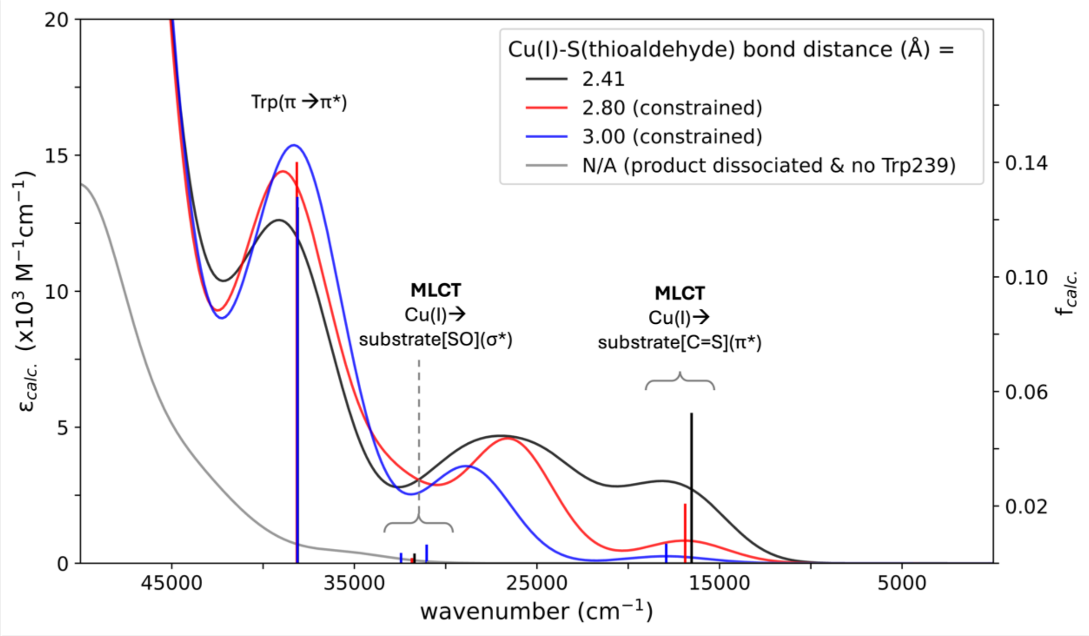

	M6 species with Cu(I)-S(thioaldehyde) bond (Å) =			
	**2.41**[a]	**2.80**[b]	**3.00**[b]	**N/A** [c]
energy (cm^{-1})	16,530	16,880	17,919	-
f_{calc} ($x10^{-2}$)	5.21	2.04	0.64	-
energy (cm^{-1})	31,707	31,848	32,442/31,040	30,978
f_{calc} ($x10^{-2}$)	0.30	0.14	0.32/0.61	0.03
Cu(I)-S(Cys) (Å)	2.30	2.23	2.22	2.19
Cu(I)-O(sulfenate) (Å)	1.99	1.95	1.94	1.90
S=C(thioaldehyde) (Å)	1.64	1.63	1.63	-

[a] *From unconstrained structure M6.*
[b] *Bond distance constrained during optimization.*
[c] *From species M8, DFT re-optimized after product dissociation from M6. Trp239 and the water molecule were also removed from the model.*

C

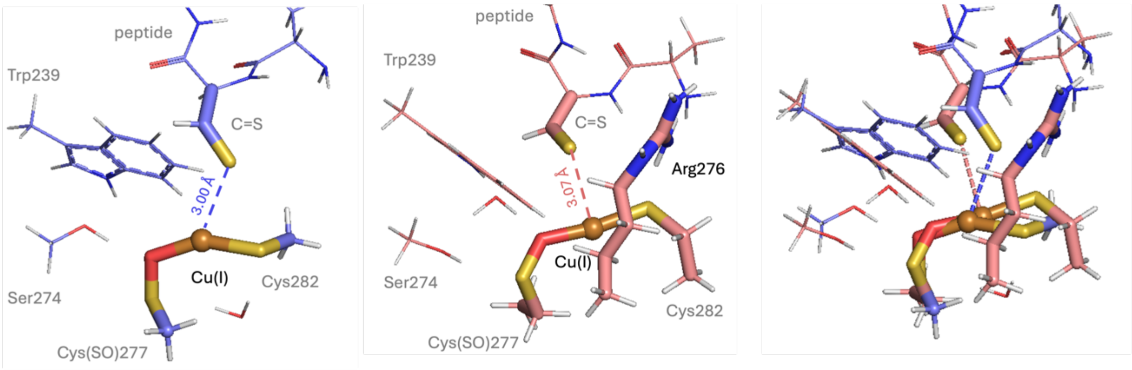

M6 with Cu-S(thioaldehyde) distance constrained to 3.00 Å

M6 with Arg276 and no bond length constraints

Overlay of two M6 structures with long Cu(I)-S bonds

(**Fig.S18,** *continues to next page*)

Figure S18. Extended TD-DFT analysis of M6 & evaluation of the Cu(I)-S(thiolate) bond length. (A) Selected transitions of the TD-DFT spectrum (*top*; cam-B3LYP/def2TZVP/N=60/ε=4.0) of M6 (S = 0) are highlighted in red and are annotated with the numbers of their associated MOs (*bottom*). The asterisk indicates a computational artifact for a Trp(π*)→thiolate transition. (B) Evaluation of the effect of its C-S(thioaldehyde) bond distance on the Cu(I)→sulfenate(σ*) and Cu(I)→thioaldehyde(π*) MLCT transitions. The TD-DFT spectra are shown at the top, and a summary table containing the corresponding energies and intensities for these transitions, and key bond distances for these structures is shown at the bottom. Increase of the Cu(I)-S(thioaldehyde) bond: (i) has minimal effect on the intensity of the higher energy Cu(I)→sulfenate(σ*) MLCT transition, and only slightly increases its energy, (ii) significantly decreases the intensity with moderate decrease in energy of the Cu(I)→thioaldehyde(π*) transition, and (iii) shows that the energy and intensity of the Trp(π→π*) transition (i.e., the internal reference transition) remains unaffected. Note that the intense absorption band around 25,000-27,000 cm^{-1} is associated with higher-energy Cu(I)→thioaldehyde(π*) MLCT transitions that also increase in energy with increasing Cu(I)-S(thioaldehyde) bond distance, and is expected to be too high in energy to be observed experimentally in the SF-Abs spectrum of intermediate B. (C) The DFT-optimized structures for M6 with the Cu(I)-S(thioaldehyde) constrained to 3.00 Å (left) and with Arg276 included but without any constrained distances (middle), as well as an overlay of these two structures (right). The longer Cu(I)-S(thioaldehyde) bond (either with constraints or in the presence of Arg276) results in similarly distorted T-shape 2S/1O Cu(I) sites.

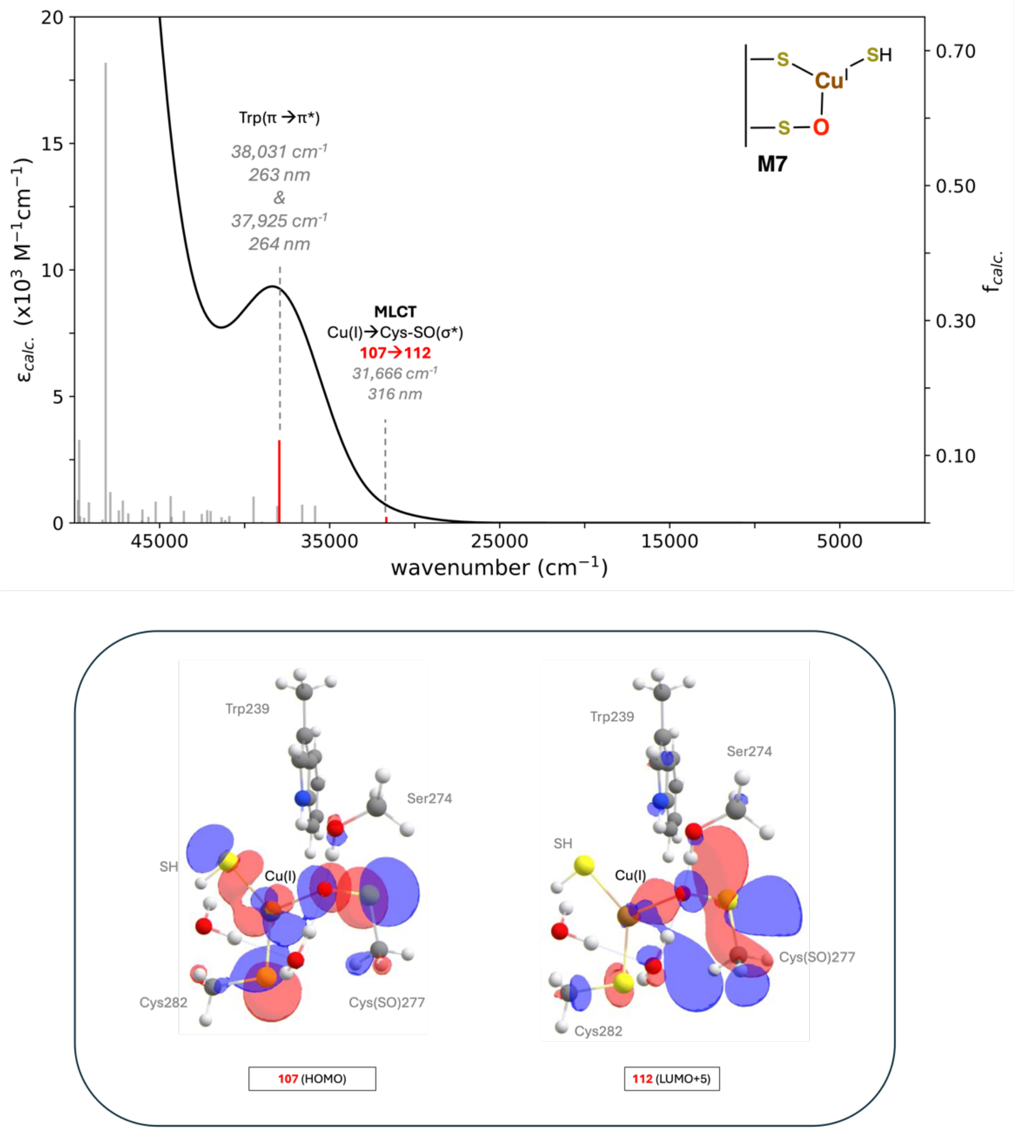

Figure S19. Extended TD-DFT analysis for the M7 species. Selected transitions of the TD-DFT spectrum (top; cam-B3LYP/def2TZVP/N=60/ε=4.0) of M7 (S = 0) are highlighted in red and are annotated with the numbers of their associated MOs (bottom). Note that this M7 model lacks the long Cu(I)-S bond observed experimentally from the EXAFS analysis for intermediate C (green spectra in Fig.3E of the main text and Table S3). Similar to the case for the smaller M6 model which lacks key second sphere interactions (i.e., Arg276, Fig.S18), inclusion of additional second-sphere interactions could result in a longer Cu(I)-S bond. However, as shown for M6 (in Fig.S18B), the energy and intensity of the Cu(I)→sulfenate(σ*) MLCT transition is insensitive to the Cu(I)-S(thioaldehyde) bond length, and thus the correlation of TD-DFT calculations to the UV-Vis absorption spectrum of intermediate C is sufficiently defined with the smaller M7 model employed here. Importantly, the TD-DFT spectrum for M7 reproduces the loss of the lower-energy band (from intermediate B to C) associated with the loss of the Cu(I)→thioaldehyde(π*) MLCT transition (shown in Fig.S18 and S19).

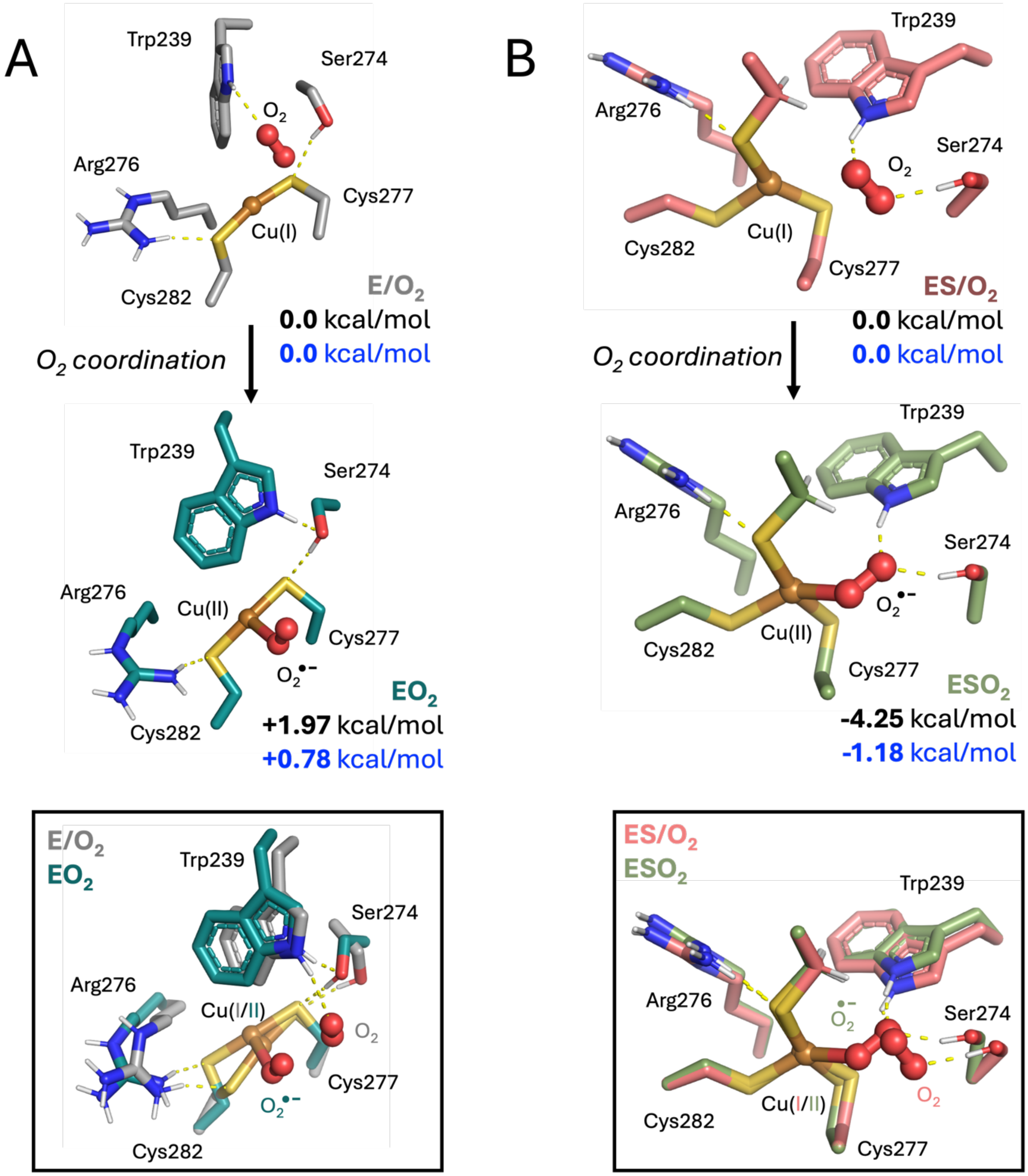

Figure S20. DFT calculations for O_2 binding & activation by E vs ES. DFT-optimized structures (B3LYP/def2SVP/ε=4.0) and relative electronic energies (in kcal/mol; the energy from DFT optimization in def2SVP are shown in black and the energies from single-point calculations of the DFT-optimized structures with def2TZVP are in blue) for O_2 coordination to the Cu(I) site of: (A) the E/O_2 intermediate (S = 0; FGE-Cu(I) without substrate and with O_2 bound in the protein pocket without metal coordination) to form the EO_2 complex containing a bis-thiolate Cu(II)-$O_2^{\bullet-}$ species (S = 1), and (B) the ES/O_2 intermediate (S = 0; as observed crystallographically, PDB: 6XTQ),[19] to form the reactive ESO_2 complex containing a tris-thiolate Cu(II)-$O_2^{\bullet-}$ species (S = 1).

These DFT calculations indicate that O_2 coordination in ES/O_2→ESO_2 is more energetically favorable (by 2 kcal/mol) than E/O_2→EO_2. Importantly, while O_2 binding in E/O_2→EO_2 requires noticeable structural changes in the metal coordination sphere (box in panel A), no structural change is required in ES/O_2→ESO_2 (box in panel B).

3. SI References:

(1) Appel, M. J.; Meier, K. K.; Lafrance-Vanasse, J.; Lim, H.; Tsai, C.-L.; Hedman, B.; Hodgson, K. O.; Tainer, J. A.; Solomon, E. I.; Bertozzi, C. R. Formylglycine-Generating Enzyme Binds Substrate Directly at a Mononuclear Cu(I) Center to Initiate O_2 Activation. *Proc. Natl. Acad. Sci. U.S.A.* **2019**, *116* (12), 5370–5375. https://doi.org/10.1073/pnas.1818274116.

(2) Carlson, B. L.; Ballister, E. R.; Skordalakes, E.; King, D. S.; Breidenbach, M. A.; Gilmore, S. A.; Berger, J. M.; Bertozzi, C. R. Function and Structure of a Prokaryotic Formylglycine-Generating Enzyme. *Journal of Biological Chemistry* **2008**, *283* (29), 20117–20125. https://doi.org/10.1074/jbc.M800217200.

(3) Tropea, J. E.; Cherry, S.; Waugh, D. S. Expression and Purification of Soluble His(6)-Tagged TEV Protease. In *Methods in molecular biology*; Clifton, N.J., 2009; Vol. 409, pp 297–307.

(4) Gill, S. C.; Von Hippel, P. H. Calculation of Protein Extinction Coefficients from Amino Acid Sequence Data. *Analytical Biochemistry* **1989**, *182* (2), 319–326. https://doi.org/10.1016/0003-2697(89)90602-7.

(5) Felsenfeld, G. The Determination of Cuprous Ion in Copper Proteins. *Archives of Biochemistry and Biophysics* **1960**, *87* (2), 247–251. https://doi.org/10.1016/0003-9861(60)90168-5.

(6) Hendrich, M. SpinCount. www.chem.cmu.edu/groups/hendrich/facilities/index.html.

(7) George, G. N. EXAFSPAK, 2000. https://www-ssrl.slac.stanford.edu/~george/exafspak/exafs.htm.

(8) Tenderholt, A.; Hedman, B.; Hodgson, K. O. PySpline: A Modern, Cross-Platform Program for the Processing of Raw Averaged XAS Edge and EXAFS Data. In *AIP Conference Proceedings*; AIP: Stanford, California (USA), 2007; Vol. 882, pp 105–107. https://doi.org/10.1063/1.2644442.

(9) Newville, M.; Stensitzki, T.; Allen, D. B.; Ingargiola, A. LMFIT: Non-Linear Least-Squares Minimization and Curve-Fitting for Python., 2023. https://doi.org/10.5281/zenodo.598352.

(10) Zabinsky, S. I.; Rehr, J. J.; Ankudinov, A.; Albers, R. C.; Eller, M. J. Multiple-Scattering Calculations of x-Ray-Absorption Spectra. *Phys. Rev. B* **1995**, *52* (4), 2995–3009. https://doi.org/10.1103/PhysRevB.52.2995.

(11) Ankudinov, A. L.; Rehr, J. J. Relativistic Calculations of Spin-Dependent x-Ray-Absorption Spectra. *Phys. Rev. B* **1997**, *56* (4), R1712–R1716. https://doi.org/10.1103/PhysRevB.56.R1712.

(12) Rehr, J. J.; Albers, R. C. Theoretical Approaches to X-Ray Absorption Fine Structure. *Rev. Mod. Phys.* **2000**, *72* (3), 621–654. https://doi.org/10.1103/RevModPhys.72.621.

(13) Rehr, J. J.; Kas, J. J.; Prange, M. P.; Sorini, A. P.; Takimoto, Y.; Vila, F. Ab Initio Theory and Calculations of X-Ray Spectra. *Comptes Rendus. Physique* **2008**, *10* (6), 548–559. https://doi.org/10.1016/j.crhy.2008.08.004.

(14) Brown, I. D.; Altermatt, D. Bond-Valence Parameters Obtained from a Systematic Analysis of the Inorganic Crystal Structure Database. *Acta Crystallogr B Struct Sci* **1985**, *41* (4), 244–247. https://doi.org/10.1107/S0108768185002063.

(15) Brese, N. E.; O'Keeffe, M. Bond-Valence Parameters for Solids. *Acta Crystallogr B Struct Sci* **1991**, *47* (2), 192–197. https://doi.org/10.1107/S0108768190011041.

(16) Thorp, H. H. Bond Valence Sum Analysis of Metal-Ligand Bond Lengths in Metalloenzymes and Model Complexes. *Inorg. Chem.* **1992**, *31* (9), 1585–1588. https://doi.org/10.1021/ic00035a012.
(17) Liu, W.; Thorp, H. H. Bond Valence Sum Analysis of Metal-Ligand Bond Lengths in Metalloenzymes and Model Complexes. 2. Refined Distances and Other Enzymes. *Inorg. Chem.* **1993**, *32* (19), 4102–4105. https://doi.org/10.1021/ic00071a023.
(18) Miarzlou, D. A.; Leisinger, F.; Joss, D.; Häussinger, D.; Seebeck, F. P. Structure of Formylglycine-Generating Enzyme in Complex with Copper and a Substrate Reveals an Acidic Pocket for Binding and Activation of Molecular Oxygen. *Chem. Sci.* **2019**, *10* (29), 7049–7058. https://doi.org/10.1039/C9SC01723B.
(19) Leisinger, F.; Miarzlou, D. A.; Seebeck, F. P. Non-Coordinative Binding of O_2 at the Active Center of a Copper-Dependent Enzyme. *Angew Chem Int Ed* **2021**, *60* (11), 6154–6159. https://doi.org/10.1002/anie.202014981.
(20) Wu, Y.; Zhao, C.; Su, Y.; Shaik, S.; Lai, W. Mechanistic Insight into Peptidyl-Cysteine Oxidation by the Copper-Dependent Formylglycine-Generating Enzyme. *Angew Chem Int Ed* **2023**, *62* (7), e202212053. https://doi.org/10.1002/anie.202212053.
(21) Quist, D. A.; Diaz, D. E.; Liu, J. J.; Karlin, K. D. Activation of Dioxygen by Copper Metalloproteins and Insights from Model Complexes. *J Biol Inorg Chem* **2017**, *22* (2–3), 253–288. https://doi.org/10.1007/s00775-016-1415-2.
(22) Iyer, S. R.; Tidemand, K. D.; Babicz, J. T.; Jacobs, A. B.; Gee, L. B.; Haahr, L. T.; Yoda, Y.; Kurokuzu, M.; Kitao, S.; Saito, M.; Seto, M.; Christensen, H. E. M.; Peters, G. H. J.; Solomon, E. I. Direct Coordination of Pterin to Fe^{II} Enables Neurotransmitter Biosynthesis in the Pterin-Dependent Hydroxylases. *Proc. Natl. Acad. Sci. U.S.A.* **2021**, *118* (15), e2022379118. https://doi.org/10.1073/pnas.2022379118.

4. SI Data: Cartesian coordinates for DFT-optimized structures. The two numbers in the header line indicate the total charge and spin multiplicity, and the numbers at the bottom of each entry indicate frozen atoms (X atom F) and constrained bond lengths (B atom1 atom2 F).

M5

-2 1

C	39.53646000	33.99041500	38.34675400
C	40.99429500	33.62847300	38.32675500
C	41.55115900	32.36220500	38.22046800
C	42.11293000	34.53228800	38.42127000
N	42.91909500	32.41741100	38.23645300
C	43.29689500	33.72382500	38.36855300
C	42.25103200	35.92939500	38.55764700
C	44.58445100	34.27565300	38.47206600
C	43.52961500	36.47948500	38.64596600
C	44.68416200	35.65896400	38.60852400
H	45.67244800	36.11971700	38.69766100
H	43.58328600	31.57740900	38.10343400
H	41.36597600	36.57290400	38.59695700
H	43.64706600	37.56226500	38.75412800
H	45.46077200	33.62040900	38.48023100
H	41.06097100	31.39082100	38.16867900
H	39.25920500	34.64332500	37.49848400
H	38.90288200	33.09082300	38.29178100
C	42.60880300	30.33539500	41.03714800
O	42.58327800	29.63481500	39.81512300
H	42.48961800	31.43183700	40.90900000
H	41.76610400	29.98152700	41.65976600
H	43.36789500	29.87240700	39.25366200
C	47.79732900	31.17418300	41.18286900
S	46.09992800	31.06145800	40.50278100
H	48.37885500	31.96676500	40.68241300
H	47.78621200	31.39121200	42.26630100
C	49.35958000	30.51694800	35.90118500
S	48.65886500	30.86615900	37.59975300
H	49.05659100	29.52255800	35.53118900
H	49.03531700	31.26903900	35.16289100
H	44.80426600	29.01924000	36.65477400
Cu	46.43890400	30.88275900	38.16654900
O	44.50173100	30.36181500	37.95039300
O	44.07503900	29.67167300	36.72172100
O	47.41438000	28.26194300	39.33961500
H	48.02526800	28.88789600	38.89081200
H	46.79610000	28.90154700	39.77004700
O	46.49391600	28.19362200	36.68376400
H	46.91275200	29.07156800	36.58948600
H	46.66126600	28.04170900	37.64326300
H	48.34962700	30.23071900	41.03316800
H	50.45994600	30.54258900	35.95946100
H	39.25800300	34.53668300	39.26665800
H	43.54798000	30.18389500	41.60256900

X 1 F

X 19 F
X 24 F
X 28 F

M6 (*no constraints*)
-1 1

C	39.53666800	33.99031500	38.34673000
C	41.00448300	33.68659400	38.20115600
C	41.65947500	32.52637200	38.56357300
C	42.02301500	34.55916400	37.66168700
N	43.00886700	32.62211200	38.28850800
C	43.26563800	33.85563600	37.74414500
C	42.01767700	35.85852800	37.11630600
C	44.47535900	34.41143800	37.30445700
C	43.21873500	36.41573400	36.67916700
C	44.43523300	35.69908200	36.77248500
H	45.36026000	36.16161000	36.41742900
H	43.71982200	31.96624600	38.60993500
H	41.08273900	36.42108500	37.03753400
H	43.22468500	37.42365800	36.25451900
H	45.40288300	33.83669900	37.36622700
H	41.26764000	31.60761400	38.99136900
H	39.06332500	34.20366500	37.37198800
H	38.99972500	33.14351400	38.80095600
C	42.60884100	30.33540100	41.03723700
O	43.05654200	29.97847800	39.74984100
H	42.52371300	31.43282900	41.17596800
H	41.60489300	29.90423200	41.19029900
H	43.97875200	30.30941300	39.62215000
C	47.79721800	31.17419800	41.18263600
S	46.28580200	30.24870100	40.68566300
H	47.55198500	32.20926500	41.46849700
H	48.19145900	30.62935800	42.05533300
C	49.35940800	30.51695800	35.90137800
S	48.81898200	31.22510900	37.51244300
H	48.89203900	29.53630000	35.71895500
H	49.10045000	31.17717100	35.05844700
N	46.86347900	28.37236400	30.59412500
H	46.95948000	27.48132000	31.08598900
C	45.44104900	28.72351000	30.49567500
H	45.38427000	29.72436700	30.03416200
C	44.72503500	27.69762600	29.61332000
H	45.25616500	27.67829600	28.64629900
H	44.86617000	26.70774600	30.07855200
C	43.22114700	27.91222800	29.38139000
H	42.72655500	27.88381600	30.36746600
C	42.90759100	29.26542700	28.72871500
H	41.82409700	29.38918600	28.57308400
H	43.40077700	29.35442700	27.74537500
H	43.24886500	30.11793400	29.33915600
C	42.64717400	26.76482300	28.54219400
H	42.83072300	25.78896900	29.02000300
H	41.55922400	26.87613000	28.40636900

H	43.10987100	26.74071000	27.54015300
C	44.79067700	28.78936800	31.89692100
O	44.23048900	27.81722000	32.38392200
N	44.86566600	29.98491700	32.58715800
C	44.08412100	30.14410400	33.79318400
C	42.58935200	30.52874600	33.52970400
O	41.79876600	30.57084800	34.45579500
C	44.58731000	30.98226900	34.94154200
S	46.10670600	31.49644400	35.28901000
H	45.40699600	30.76581400	32.23013500
H	43.96724800	29.12372300	34.21011700
H	43.77752400	31.19857200	35.65098900
N	42.24774700	30.77888700	32.23585000
H	42.98079100	30.77317500	31.53703100
C	40.90648400	31.15198100	31.84268000
H	40.31121400	31.24440500	32.76845700
H	40.88190500	32.12429300	31.32451800
C	40.22018700	30.11301100	30.96720400
O	39.44057000	30.38653300	30.08909400
Cu	46.52110800	31.11850700	37.63373300
O	45.59533500	31.06487200	39.39645500
O	47.38661500	28.39839600	38.22343000
H	48.04782200	29.10368100	38.06039000
H	47.02175200	28.70401400	39.07719900
H	48.53957600	31.16871900	40.36897100
H	50.45368500	30.38272800	35.90918300
H	47.36864400	29.05860200	31.15638300
H	40.49620900	29.05310800	31.20897200
H	39.36837300	34.87683000	38.98325200
H	43.27031700	29.95378600	41.83979500

X 1 F
X 19 F
X 24 F
X 28 F
X 34 F
X 39 F
X 52 F
X 65 F

M6 (*C-S constrained to 3.00 Å*)
-1 1

C	39.53666300	33.99033500	38.34671800
C	40.95247400	33.54446600	38.12905800
C	41.50386400	32.32936300	38.47711000
C	42.02392100	34.30485200	37.52984700
N	42.83698300	32.28567500	38.13447700
C	43.19411000	33.48153800	37.56236400
C	42.11688000	35.59697300	36.97515400
C	44.43012700	33.92130100	37.06502000
C	43.34313200	36.03463400	36.47772700
C	44.48761400	35.20432200	36.52444200
H	45.43754100	35.57208400	36.12713900

H	43.44912300	31.52078600	38.40568300
H	41.23705600	36.24600500	36.93713400
H	43.42624900	37.03574300	36.04514900
H	45.31020300	33.27508100	37.09210700
H	41.04034100	31.46790900	38.95231000
H	39.03133700	34.23683000	37.39593100
H	38.94475700	33.20701300	38.84482400
C	42.60883700	30.33536100	41.03728200
O	43.18093100	29.92493900	39.81428500
H	42.58748900	31.43794500	41.15639800
H	41.56544000	29.97925400	41.07308200
H	44.13756500	30.18798300	39.75892600
C	47.79718700	31.17422300	41.18264800
S	46.68156000	29.88320100	40.50794100
H	47.23093400	31.96179500	41.70426000
H	48.47161700	30.66157700	41.88633000
C	49.35952200	30.51694300	35.90136000
S	48.76899800	31.92231700	36.94357400
H	49.46792700	29.61064600	36.51499300
H	48.67293700	30.30804800	35.06722100
N	46.74341500	28.45914800	30.00210800
H	46.85735600	27.56370000	30.48180000
C	45.31621700	28.79485900	29.92609600
H	45.24242600	29.79908500	29.47421100
C	44.59978000	27.76834400	29.04517100
H	45.13103700	27.74827700	28.07835800
H	44.74106200	26.77920200	29.51180800
C	43.09627400	27.98356900	28.81181500
H	42.60283100	27.97471200	29.79923900
C	42.78613400	29.32507400	28.13375300
H	41.70201900	29.45417500	27.98713600
H	43.26913500	29.38815400	27.14348200
H	43.14161900	30.18910800	28.71950000
C	42.51638300	26.82289900	27.99531200
H	42.69358000	25.85587500	28.49292600
H	41.42935700	26.93807800	27.85570800
H	42.98034000	26.77615900	26.99471900
C	44.68342500	28.84311800	31.33889200
O	44.12567300	27.86837000	31.82282300
N	44.77152400	30.03162300	32.04085500
C	43.95914600	30.21545200	33.22354800
C	42.49317400	30.67263900	32.91982700
O	41.70152400	30.82057500	33.83511800
C	44.47505200	30.98383400	34.41800000
S	45.98859200	31.20524600	34.96885800
H	45.29324500	30.81868200	31.66860900
H	43.78125500	29.19419400	33.61791400
H	43.63303100	31.36099900	35.01754400
N	42.16420600	30.84499100	31.60988700
H	42.89441900	30.76026900	30.91374200
C	40.84146400	31.25250600	31.18662500
H	40.25479600	31.45126400	32.10088900
H	40.86552100	32.17921000	30.59093900

C	40.09534100	30.18436200	30.39762700
O	39.31742800	30.43111700	29.51041000
Cu	46.87454300	31.18334900	37.83500800
O	45.77098200	30.67024100	39.34730400
O	49.64313900	29.56572800	39.07658800
H	49.51566400	30.39984600	38.57840700
H	48.72981700	29.37791300	39.36587600
H	48.39111700	31.60898400	40.36424900
H	50.34657000	30.77166300	35.48188500
H	47.25006900	29.14533900	30.56292400
H	40.32332800	29.13275300	30.71432900
H	39.48822200	34.89753300	38.97485400
H	43.13898400	29.91480600	41.91528500

X 1 F
X 19 F
X 24 F
X 28 F
X 34 F
X 39 F
X 52 F
X 65 F
B 56 67 F

M6 (*small model, without thioaldehyde*)
-1 1

C	42.60876100	30.33545800	41.03709800
O	43.12730100	29.96442400	39.79000000
H	42.55408200	31.43695100	41.18924300
H	41.57669900	29.95009600	41.12482200
H	44.08332500	30.22223600	39.71777200
C	47.79742200	31.17415000	41.18280900
S	46.60244900	29.85213900	40.79862600
H	47.27968600	32.11422000	41.43651800
H	48.40575300	30.84528900	42.04203000
C	49.35948700	30.51697700	35.90129800
S	48.11943600	31.79947000	36.39656200
H	49.99878000	30.21416500	36.74653000
H	48.87943800	29.61114000	35.49621700
Cu	46.88834500	30.97030500	38.00378300
O	45.79745500	30.49339300	39.48332500
H	48.45112800	31.35235800	40.31129700
H	50.00771700	30.93987400	35.11632600
H	43.18532900	29.92647500	41.89500600

X 1 F
X 6 F
X 10 F

M6 (*extended model to include Arg276, no constraints*)
0 1

C	-12.91605100	-2.27597500	-12.35001500
C	-11.55038400	-2.88275100	-12.37821300

C	-11.22118500	-4.22303300	-12.32200600
C	-10.29939700	-2.17449500	-12.46040100
N	-9.85687800	-4.39313400	-12.36760100
C	-9.26299300	-3.15908600	-12.44636600
C	-9.94897900	-0.81095500	-12.53837100
C	-7.90591100	-2.80832700	-12.50711600
C	-8.60175600	-0.46067900	-12.59519900
C	-7.59131100	-1.45209200	-12.57757300
H	-6.54204800	-1.14574000	-12.61436900
H	-9.37491100	-5.30687500	-12.27274300
H	-10.72561800	-0.04059300	-12.54996800
H	-8.31689100	0.59391800	-12.64940400
H	-7.14970400	-3.59314800	-12.49757400
H	-11.88065500	-5.08749900	-12.24905200
C	-10.05108400	-5.35698400	-8.83896800
O	-8.95251300	-6.17738700	-9.21027800
H	-10.28949300	-4.59842000	-9.60694700
H	-10.93025800	-6.00865700	-8.71893100
H	-8.15409000	-5.62622100	-9.35393300
H	-9.87621300	-4.83996000	-7.87664600
C	-5.50995000	-7.54798500	-9.47001300
C	-4.88740900	-6.16489500	-9.59871700
S	-6.02565700	-4.92722000	-10.30718800
H	-6.38146700	-7.52705800	-8.79621500
H	-4.77981000	-8.28040200	-9.08738400
H	-5.85914500	-7.89017300	-10.45704400
H	-4.57584900	-5.75163700	-8.62352800
H	-3.99875100	-6.19040000	-10.25470200
C	-1.47492600	-6.58403000	-14.08909100
C	-2.50262800	-5.95112700	-15.01989000
S	-4.16324300	-6.77483900	-14.99275100
H	-1.81570500	-6.52113200	-13.04252200
H	-1.32752400	-7.64902700	-14.32826000
H	-0.50011600	-6.07151100	-14.16790400
H	-2.62559400	-4.88291900	-14.77908700
H	-2.15300100	-6.00181100	-16.06452500
C	-3.16902700	-2.22700300	-11.78993900
C	-4.04707500	-2.96439600	-12.78981100
C	-4.08730900	-2.33604300	-14.18666200
C	-5.16213400	-3.01384400	-15.03135400
N	-5.15989200	-2.61763700	-16.43742800
C	-5.22711100	-3.45234300	-17.48551900
N	-5.00884400	-4.75330700	-17.36915300
N	-5.52039600	-2.93969500	-18.70119000
H	-3.51049700	-1.18712400	-11.65038700
H	-3.70328100	-4.01050100	-12.88175700
H	-5.06757400	-3.03696000	-12.38581200
H	-4.29660600	-1.25302400	-14.11055000
H	-3.10509000	-2.43421600	-14.68165600
H	-5.02563900	-4.09953700	-14.97122600
H	-6.15505200	-2.80445000	-14.60442300
H	-5.27895200	-1.62766500	-16.62568800
H	-4.80787700	-5.22306500	-16.46687500

H	-5.26323900	-5.43151700	-18.11171600
H	-5.70118700	-1.95213800	-18.82615200
H	-5.36320900	-3.49062400	-19.53528700
N	-5.55044300	-7.08044400	-18.97793700
C	-6.79848900	-7.56006100	-19.58898500
C	-7.87115900	-7.90533400	-18.54447200
O	-8.78949900	-8.66692600	-18.81615700
C	-6.61901900	-8.72702800	-20.55602000
H	-5.16835100	-7.78557000	-18.34076400
H	-4.84391300	-6.96622200	-19.70739400
H	-7.22909300	-6.70840700	-20.14946700
H	-6.19701000	-9.60130700	-20.03436400
H	-5.93658700	-8.44563800	-21.37335600
N	-7.79811900	-7.26968000	-17.34540300
C	-8.83826000	-7.46293800	-16.36167000
C	-10.25248900	-7.06964800	-16.91920500
O	-11.25229700	-7.66519900	-16.56508000
C	-8.61858700	-6.65909200	-15.11109900
S	-7.62062500	-5.38326100	-14.98307400
H	-8.94942000	-8.52802100	-16.10208300
H	-9.20690500	-6.98065700	-14.24004400
H	-7.03087200	-6.64229600	-17.11156900
N	-10.21992800	-6.01150600	-17.76311100
C	-11.34794300	-5.59499400	-18.56695600
H	-11.48726300	-4.50374500	-18.51339600
H	-12.24956700	-6.08510900	-18.17671900
H	-9.30936200	-5.61964000	-17.97416300
Cu	-5.22037800	-6.07143000	-13.20098800
O	-8.66773500	-6.88053100	-11.91339700
H	-3.20274000	-2.72268100	-10.80731200
H	-2.11669600	-2.19010800	-12.11933200
H	-7.58481900	-9.02300300	-20.98615000
H	-11.21935300	-5.88630500	-19.62349900
H	-7.75678400	-6.46743500	-11.91043700
H	-8.89951000	-6.86024100	-10.96224400
O	-6.36546300	-5.60809400	-11.77699700
H	-13.04254900	-1.59297100	-11.49110000
H	-13.12051700	-1.67979700	-13.25734400
H	-13.69767900	-3.04815600	-12.27805400

X 1 F
X 17 F
X 23 F
X 31 F
X 39 F
X 63 F
X 79 F

M7
-2 1

C	39.636964	33.965124	38.337596
C	41.062595	33.587885	38.040028
C	41.641133	32.331442	38.024805

C	42.100705	34.503845	37.635517
N	42.963703	32.415814	37.652758
C	43.272954	33.729751	37.400414
C	42.145014	35.895213	37.433869
C	44.480275	34.312219	36.988932
C	43.343910	36.476750	37.031336
C	44.500655	35.693345	36.817127
H	45.421456	36.190188	36.503589
H	43.620255	31.644418	37.536272
H	41.244882	36.495991	37.577808
H	43.397372	37.551996	36.857560
H	45.354131	33.678060	36.839139
H	41.180475	31.372035	38.238893
H	39.290766	34.616303	37.519083
H	38.976970	33.082948	38.324461
C	42.758217	30.488022	41.051532
O	43.808218	29.751695	40.514926
H	42.242980	31.148330	40.324299
H	41.985806	29.779075	41.412781
H	44.536014	30.338726	40.124209
C	47.752518	31.152814	41.579470
S	46.651397	32.150584	40.476280
H	48.707489	31.697559	41.552774
H	47.390633	31.121573	42.619797
C	49.463944	30.543952	36.058439
S	49.084999	30.710755	37.860803
H	49.054885	29.600202	35.662694
H	48.946419	31.345705	35.510749
N	46.853284	28.530057	30.229992
H	47.210215	27.640917	30.590561
C	45.408360	28.745847	30.298135
H	45.177727	29.782717	30.010438
C	44.708567	27.784444	29.315411
H	45.228001	27.870482	28.348373
H	44.884291	26.761850	29.685047
C	43.198485	28.018288	29.104051
H	42.740440	28.254204	30.083777
C	42.928238	29.198087	28.159596
H	41.862975	29.475296	28.170044
H	43.193788	28.933719	27.122885
H	43.505473	30.094645	28.434213
C	42.537748	26.735995	28.581295
H	42.537764	25.947872	29.349244
H	41.496457	26.910343	28.269490
H	43.070819	26.346158	27.698585
C	44.868379	28.474530	31.710906
O	44.959908	27.359803	32.212400
N	44.240164	29.506429	32.338150
C	43.380265	29.245848	33.478279
C	42.176822	30.190470	33.575701
O	41.546644	30.306582	34.618639
C	44.115351	28.911525	34.781667
S	45.876311	31.238010	35.503678

H	44.257821	30.429597	31.902214
H	42.900916	28.264471	33.274379
H	45.676654	28.013585	36.714248
H	45.190169	28.699216	34.643685
N	41.763540	30.738015	32.410320
H	42.388228	30.760320	31.604042
C	40.388260	31.146795	32.165737
H	39.825251	31.033668	33.100375
H	40.326024	32.189108	31.819319
C	39.843832	30.249334	31.045704
O	39.682966	30.750121	29.950399
Cu	46.749703	31.077019	37.667865
O	45.795629	31.144888	39.494449
O	43.543242	28.660712	35.819644
O	48.400611	27.561869	38.052337
H	48.530999	28.536843	38.132491
H	48.045416	27.266477	38.919835
O	46.382604	27.567190	36.223115
H	46.565200	30.076707	35.401729
H	47.127654	27.482629	36.869083
H	47.907816	30.126088	41.248136
H	50.521956	30.569162	35.797594
H	47.502370	29.286154	30.313515
H	39.647875	29.187801	31.196752
H	39.499494	34.486308	39.284979
H	43.010319	31.140653	41.887345

X 1 F
X 19 F
X 24 F
X 28 F
X 34 F
X 39 F
X 52 F
X 66 F

E/O_2
0 3 0 1 0 1

C(Fragment=1)	-9.18494900	-8.64201000	-24.02904500
C(Fragment=1)	-9.91785800	-7.56934600	-24.85222100
C(Fragment=1)	-9.00974400	-6.92730300	-25.85732000
C(Fragment=1)	-8.99645800	-7.07586800	-27.22657300
C(Fragment=1)	-7.85261000	-6.13355600	-25.52762400
C(Fragment=1)	-7.17332500	-5.84823200	-26.74780700
C(Fragment=1)	-7.30906200	-5.68114100	-24.31011500
N(Fragment=1)	-7.90746100	-6.41406100	-27.76521800
C(Fragment=1)	-5.95337300	-5.16236200	-26.76900200
C(Fragment=1)	-6.09607900	-4.99958500	-24.32918000
C(Fragment=1)	-5.42427700	-4.76114400	-25.54684100
H(Fragment=1)	-8.28540400	-8.23373300	-23.54557100
H(Fragment=1)	-9.83616400	-9.05665700	-23.24168900
H(Fragment=1)	-8.86296600	-9.47461000	-24.67377700
H(Fragment=1)	-10.31902200	-6.80033300	-24.16693100

H(Fragment=1)	-10.78926400	-8.01638700	-25.35901300
H(Fragment=1)	-9.68508600	-7.62295000	-27.86868300
H(Fragment=1)	-7.82109900	-5.88102600	-23.36563200
H(Fragment=1)	-7.63255900	-6.42484900	-28.73837600
H(Fragment=1)	-5.40561400	-4.99006500	-27.69570600
H(Fragment=1)	-5.64381600	-4.66560200	-23.39163800
H(Fragment=1)	-4.44853800	-4.27944500	-25.53830500
C(Fragment=1)	-5.78416800	-9.08323900	-26.03637400
C(Fragment=1)	-5.53178500	-9.20070800	-27.53595600
O(Fragment=1)	-4.16899100	-9.07831100	-27.87485600
H(Fragment=1)	-6.85252100	-9.20733500	-25.80267500
H(Fragment=1)	-5.20525900	-9.84410700	-25.48782200
H(Fragment=1)	-6.14963900	-8.44592200	-28.06578500
H(Fragment=1)	-5.86671700	-10.18914400	-27.89961000
H(Fragment=1)	-3.84703200	-8.22524300	-27.51339900
H(Fragment=1)	-5.47949600	-8.08968700	-25.67156800
C(Fragment=1)	-0.33814900	-6.18812000	-28.50199600
C(Fragment=1)	-1.33718400	-7.19811300	-27.94726800
S(Fragment=1)	-2.55393200	-6.48573300	-26.74473300
H(Fragment=1)	-0.85089900	-5.41578000	-29.09869600
H(Fragment=1)	0.19687200	-5.67494400	-27.68647300
H(Fragment=1)	-0.81012000	-8.01444900	-27.42620400
H(Fragment=1)	-1.90381000	-7.66154100	-28.77177400
H(Fragment=1)	0.40895900	-6.68051600	-29.15034800
C(Fragment=1)	-0.26408800	-0.72713200	-28.12096200
C(Fragment=1)	-0.90012700	-1.98059400	-27.53518300
S(Fragment=1)	-2.48482900	-2.42617100	-28.39466400
H(Fragment=1)	-0.05372400	-0.85511300	-29.19495200
H(Fragment=1)	-0.93171600	0.14408900	-28.01062500
H(Fragment=1)	-1.10208200	-1.84481700	-26.46043400
H(Fragment=1)	-0.21507000	-2.83781100	-27.62350700
H(Fragment=1)	0.68545600	-0.49015000	-27.61034800
C(Fragment=1)	-1.99603000	-2.27002800	-23.02304100
C(Fragment=1)	-3.06530600	-2.46597300	-24.09459600
C(Fragment=1)	-3.66407600	-1.15060400	-24.59890200
C(Fragment=1)	-4.80523800	-1.37181200	-25.59510200
N(Fragment=1)	-5.43533400	-0.12412200	-26.03436300
C(Fragment=1)	-4.99132300	0.67752800	-27.00921600
N(Fragment=1)	-4.00316400	0.30303600	-27.81670600
N(Fragment=1)	-5.55687200	1.89123100	-27.16140900
H(Fragment=1)	-2.40644200	-1.75352200	-22.13895600
H(Fragment=1)	-1.58448800	-3.23538400	-22.68962200
H(Fragment=1)	-3.87255200	-3.09560200	-23.68564400
H(Fragment=1)	-2.64765000	-3.02408800	-24.95208100
H(Fragment=1)	-2.87782900	-0.54286800	-25.08132800
H(Fragment=1)	-1.15664100	-1.66274500	-23.40233700
H(Fragment=1)	-4.04013600	-0.55834600	-23.74484900
H(Fragment=1)	-5.59848900	-1.98621700	-25.14713700
H(Fragment=1)	-4.44045700	-1.93095800	-26.47009800
H(Fragment=1)	-6.16579300	0.24771900	-25.43794200
H(Fragment=1)	-3.71511100	0.92252200	-28.56456600
H(Fragment=1)	-3.60094900	-0.66505600	-27.85905300
H(Fragment=1)	-5.33918800	2.46679600	-27.96486300

O(Fragment=2) -5.38202600 -6.54434600 -29.76577500
O(Fragment=2) -4.20165500 -6.65342800 -29.53714300
Cu(Fragment=1) -2.72639200 -4.43000700 -27.51447100
H(Fragment=1) -6.31221900 2.19843500 -26.56185000

X 1 F
X 24 F
X 32 F
X 40 F
X 48 F

EO_2
0 3 0 1 0 1
C(Fragment=1) -9.18499700 -8.64200800 -24.02899700
C(Fragment=1) -9.85983200 -7.66622700 -25.00862100
C(Fragment=1) -8.89202300 -7.10853000 -26.01205200
C(Fragment=1) -8.60784000 -7.62071200 -27.25998000
C(Fragment=1) -7.96554000 -6.01753000 -25.81069200
C(Fragment=1) -7.16006100 -5.94020700 -26.98611300
C(Fragment=1) -7.71533800 -5.11979600 -24.75266300
N(Fragment=1) -7.59763700 -6.90152800 -27.86831100
C(Fragment=1) -6.11186300 -5.02153200 -27.11450400
C(Fragment=1) -6.67545700 -4.19951700 -24.87887600
C(Fragment=1) -5.88050200 -4.15785400 -26.04692600
H(Fragment=1) -8.36408800 -8.14697700 -23.48549300
H(Fragment=1) -9.90155100 -9.02998900 -23.28579900
H(Fragment=1) -8.75273500 -9.49844700 -24.57063100
H(Fragment=1) -10.32729400 -6.84266000 -24.44045000
H(Fragment=1) -10.68176400 -8.18353700 -25.53201000
H(Fragment=1) -9.06139200 -8.47149500 -27.76745900
H(Fragment=1) -8.31830700 -5.15643200 -23.84098400
H(Fragment=1) -6.96081100 -7.29687300 -28.56680700
H(Fragment=1) -5.48335900 -5.00362600 -28.00554300
H(Fragment=1) -6.45824700 -3.50790400 -24.06030200
H(Fragment=1) -5.05303700 -3.44914300 -26.12177900
C(Fragment=1) -4.37826600 -10.16834100 -27.30039800
C(Fragment=1) -5.53201100 -9.20099000 -27.53599800
O(Fragment=1) -5.26801300 -8.29684100 -28.60221300
H(Fragment=1) -4.62876200 -10.89675100 -26.51108900
H(Fragment=1) -4.14460100 -10.72003800 -28.22536100
H(Fragment=1) -5.76605500 -8.65236000 -26.60249100
H(Fragment=1) -6.44519700 -9.75663800 -27.81007800
H(Fragment=1) -4.55342900 -7.68997300 -28.28681000
H(Fragment=1) -3.47703900 -9.61655500 -26.98917000
C(Fragment=1) -0.33798800 -6.18799700 -28.50200300
C(Fragment=1) -1.65532300 -6.94639300 -28.65513900
S(Fragment=1) -2.84622500 -6.66478500 -27.26708600
H(Fragment=1) -0.50770700 -5.10005300 -28.54054500
H(Fragment=1) 0.13987900 -6.41776300 -27.53643300
H(Fragment=1) -1.47189200 -8.03229500 -28.70602100
H(Fragment=1) -2.14734300 -6.65662200 -29.59789300
H(Fragment=1) 0.36700200 -6.44831500 -29.31138100
C(Fragment=1) -0.26399900 -0.72700800 -28.12099600

C(Fragment=1)	-0.97107400	-2.07377400	-28.21699300
S(Fragment=1)	-2.12706400	-2.34343500	-26.79992800
H(Fragment=1)	-0.99004800	0.10329700	-28.13030100
H(Fragment=1)	0.31514900	-0.65186100	-27.18683100
H(Fragment=1)	-0.23098700	-2.88916800	-28.21343200
H(Fragment=1)	-1.53414300	-2.16224200	-29.16017300
H(Fragment=1)	0.42891000	-0.58352700	-28.96804700
C(Fragment=1)	-1.99600500	-2.26999700	-23.02300600
C(Fragment=1)	-3.40934600	-2.10732800	-23.58009600
C(Fragment=1)	-3.65102800	-0.71473500	-24.17274600
C(Fragment=1)	-4.95314500	-0.64523100	-24.97112900
N(Fragment=1)	-5.14215300	0.61228800	-25.69390100
C(Fragment=1)	-4.83923700	0.82896200	-26.98049100
N(Fragment=1)	-4.19954000	-0.08177500	-27.71002800
N(Fragment=1)	-5.21556700	1.99502800	-27.54096300
H(Fragment=1)	-1.77613000	-1.51349000	-22.25020500
H(Fragment=1)	-1.85703800	-3.26432000	-22.56971900
H(Fragment=1)	-4.16164300	-2.31479500	-22.79755300
H(Fragment=1)	-3.56667800	-2.86723800	-24.36439500
H(Fragment=1)	-2.81733900	-0.46392500	-24.84687600
H(Fragment=1)	-1.25751400	-2.16279700	-23.83301800
H(Fragment=1)	-3.66247200	0.04671900	-23.37207900
H(Fragment=1)	-5.82687800	-0.78235800	-24.31462000
H(Fragment=1)	-4.99065200	-1.46006500	-25.70598100
H(Fragment=1)	-5.46827200	1.41044000	-25.15980100
H(Fragment=1)	-4.10637700	0.06814800	-28.70773300
H(Fragment=1)	-3.62093700	-0.86526300	-27.31376700
H(Fragment=1)	-4.91449500	2.24399000	-28.47446200
O(Fragment=2)	-4.19752200	-4.42553100	-29.98175300
O(Fragment=2)	-3.13534900	-4.12403000	-29.44285800
Cu(Fragment=1)	-2.75846500	-4.43968500	-27.21808700
H(Fragment=1)	-5.88297900	2.60515000	-27.08579200

X 1 F
X 24 F
X 32 F
X 40 F
X 48 F

ES/O_2
-1 3 0 1 0 1

C(Fragment=1)	-9.25939800	-8.62030400	-24.22690000
C(Fragment=1)	-9.33210500	-7.87752600	-25.57770200
C(Fragment=1)	-8.18330800	-6.94706800	-25.87131600
C(Fragment=1)	-7.21495200	-7.09753900	-26.84882000
C(Fragment=1)	-7.87957900	-5.70070900	-25.20756300
C(Fragment=1)	-6.70946600	-5.16765400	-25.83506400
C(Fragment=1)	-8.47916400	-4.96608400	-24.16408900
N(Fragment=1)	-6.33553000	-6.03882100	-26.82414400
C(Fragment=1)	-6.13245900	-3.95204900	-25.44224700
C(Fragment=1)	-7.91282500	-3.75375500	-23.77426000
C(Fragment=1)	-6.74862300	-3.25400400	-24.40525300
H(Fragment=1)	-10.14655800	-9.25750400	-24.07822800

H(Fragment=1)	-8.36319000	-9.26024700	-24.18069200
H(Fragment=1)	-9.20212600	-7.91205100	-23.38469700
H(Fragment=1)	-10.27787400	-7.30433100	-25.60852000
H(Fragment=1)	-9.40270700	-8.61913500	-26.39137100
H(Fragment=1)	-7.08108100	-7.90207100	-27.56941600
H(Fragment=1)	-9.37826700	-5.34312700	-23.66828600
H(Fragment=1)	-5.51520600	-5.93470900	-27.42108100
H(Fragment=1)	-5.23164000	-3.59062600	-25.94189400
H(Fragment=1)	-8.37003600	-3.17826700	-22.96435300
H(Fragment=1)	-6.32357600	-2.30532800	-24.06694900
C(Fragment=1)	-4.52583900	-9.81558000	-26.23286700
C(Fragment=1)	-5.86332200	-10.23916500	-26.82315700
O(Fragment=1)	-6.00156400	-9.91918600	-28.19828400
H(Fragment=1)	-4.46313100	-10.09619200	-25.16876800
H(Fragment=1)	-3.69336000	-10.30117400	-26.76931200
H(Fragment=1)	-6.67936900	-9.77119200	-26.23510000
H(Fragment=1)	-5.99456600	-11.33313900	-26.72333500
H(Fragment=1)	-5.39904800	-9.18177400	-28.41680100
H(Fragment=1)	-4.37879200	-8.72623000	-26.31170000
C(Fragment=1)	-0.72473900	-6.51661600	-28.39721400
C(Fragment=1)	-1.06651700	-6.38029800	-26.91083300
S(Fragment=1)	-2.82270100	-5.94295700	-26.52274400
H(Fragment=1)	-1.39697000	-7.24214300	-28.88264000
H(Fragment=1)	-0.85083600	-5.54220700	-28.89511200
H(Fragment=1)	-0.42930400	-5.59996600	-26.46140100
H(Fragment=1)	-0.84985200	-7.32327300	-26.37948500
H(Fragment=1)	0.32054400	-6.84842500	-28.53397100
C(Fragment=1)	-0.20228300	-0.31298700	-28.23667000
C(Fragment=1)	-1.26706800	-1.35856000	-28.55450400
S(Fragment=1)	-1.09766800	-2.85841200	-27.50629300
H(Fragment=1)	0.80978400	-0.72578500	-28.38267400
H(Fragment=1)	-0.30101200	0.58127900	-28.88102800
H(Fragment=1)	-1.21439600	-1.63784400	-29.62134200
H(Fragment=1)	-2.26714200	-0.92097100	-28.40834200
H(Fragment=1)	-0.27179100	0.01358800	-27.18535000
C(Fragment=1)	-1.72117600	-2.35711600	-23.68013000
C(Fragment=1)	-3.00330200	-2.21498300	-24.49634800
C(Fragment=1)	-3.07174900	-0.86307000	-25.20466500
C(Fragment=1)	-4.21614900	-0.76465700	-26.20766700
N(Fragment=1)	-4.29920400	0.59771700	-26.74393100
C(Fragment=1)	-4.57138700	0.97536000	-27.99616700
N(Fragment=1)	-4.76611500	0.09710800	-28.97102100
N(Fragment=1)	-4.68182100	2.30083600	-28.25490200
H(Fragment=1)	-1.69317800	-3.32034000	-23.14590400
H(Fragment=1)	-0.85141200	-2.32239300	-24.35577900
H(Fragment=1)	-3.89128800	-2.35044100	-23.85423900
H(Fragment=1)	-3.02505400	-3.01598300	-25.25265600
H(Fragment=1)	-2.12810200	-0.70365300	-25.75050300
H(Fragment=1)	-1.61719600	-1.55053600	-22.93255600
H(Fragment=1)	-3.16404200	-0.05380500	-24.45284000
H(Fragment=1)	-5.18192800	-1.02728900	-25.74179600
H(Fragment=1)	-4.04591900	-1.48087600	-27.02563400
H(Fragment=1)	-4.20916300	1.34053400	-26.05865700

H(Fragment=1) -4.89806300 0.43977700 -29.91532700
H(Fragment=1) -4.59500700 -0.95528500 -28.90983400
H(Fragment=1) -4.40775100 2.98417300 -27.56016600
H(Fragment=1) -4.72026000 2.62555100 -29.21268200
C(Fragment=1) -6.91156300 -2.49639900 -30.27621900
C(Fragment=1) -6.11214600 -3.41471600 -29.34902100
S(Fragment=1) -4.32438400 -2.95265600 -29.33283700
H(Fragment=1) -6.87309200 -1.45063600 -29.92951900
H(Fragment=1) -6.50834700 -2.52648500 -31.30191000
H(Fragment=1) -6.19892000 -4.46512500 -29.66816900
H(Fragment=1) -6.51276300 -3.36318300 -28.32334600
H(Fragment=1) -7.97373900 -2.79752400 -30.31722300
Cu(Fragment=1) -3.08323200 -4.08869300 -27.78703400
O(Fragment=2) -4.88585900 -6.73324000 -29.32997700
O(Fragment=2) -4.33636600 -7.68585700 -28.72516500

X 1 F
X 23 F
X 32 F
X 40 F
X 48 F
X 70 F

ESO_2
-1 3 0 1 0 1
C(Fragment=1) -9.25898700 -8.61994000 -24.22696600
C(Fragment=1) -9.75549000 -7.55370400 -25.22305100
C(Fragment=1) -8.66320200 -6.62104400 -25.66504300
C(Fragment=1) -7.85664500 -6.75554100 -26.77889900
C(Fragment=1) -8.13136500 -5.49975700 -24.93163400
C(Fragment=1) -6.99416900 -5.02638800 -25.66383700
C(Fragment=1) -8.49194000 -4.84895100 -23.73476400
N(Fragment=1) -6.86331500 -5.80538500 -26.78326300
C(Fragment=1) -6.21934700 -3.94568400 -25.21682500
C(Fragment=1) -7.73350100 -3.76478800 -23.29860500
C(Fragment=1) -6.60728200 -3.32136100 -24.03153600
H(Fragment=1) -10.07577900 -9.28974200 -23.90866500
H(Fragment=1) -8.46606800 -9.23360600 -24.68332100
H(Fragment=1) -8.83314300 -8.14773500 -23.32690800
H(Fragment=1) -10.57781000 -6.97926400 -24.75923500
H(Fragment=1) -10.19156500 -8.05608300 -26.10353700
H(Fragment=1) -7.87715100 -7.50930000 -27.56489500
H(Fragment=1) -9.35435800 -5.19336900 -23.15621100
H(Fragment=1) -6.06298600 -5.83255600 -27.45233900
H(Fragment=1) -5.33955200 -3.62552100 -25.77956800
H(Fragment=1) -8.00245000 -3.25120200 -22.37114300
H(Fragment=1) -6.02575600 -2.47832800 -23.65115600
C(Fragment=1) -4.52590400 -9.81597100 -26.23305500
C(Fragment=1) -5.90965700 -9.37475100 -26.68952100
O(Fragment=1) -5.97656000 -9.03093900 -28.05974900
H(Fragment=1) -4.54612600 -10.14690600 -25.18102300
H(Fragment=1) -4.14996600 -10.64400800 -26.85655500
H(Fragment=1) -6.25173800 -8.54354100 -26.04422300

H(Fragment=1)	-6.63330800	-10.19769300	-26.54272900
H(Fragment=1)	-5.46036900	-8.21614500	-28.23346200
H(Fragment=1)	-3.81738500	-8.97206900	-26.30944500
C(Fragment=1)	-0.72404800	-6.51698900	-28.39695600
C(Fragment=1)	-0.76022200	-6.14962800	-26.90958300
S(Fragment=1)	-2.42799500	-5.70517700	-26.23976500
H(Fragment=1)	-1.45319600	-7.30963600	-28.62921200
H(Fragment=1)	-0.97862000	-5.63792700	-29.01020900
H(Fragment=1)	-0.07534000	-5.30375600	-26.73169200
H(Fragment=1)	-0.39496200	-6.99648600	-26.30216400
H(Fragment=1)	0.28477400	-6.86047100	-28.69153600
C(Fragment=1)	-0.20198600	-0.31304100	-28.23697900
C(Fragment=1)	-1.37050800	-1.25521300	-28.52652600
S(Fragment=1)	-1.22797000	-2.80250500	-27.54458400
H(Fragment=1)	0.76028600	-0.79389900	-28.47850700
H(Fragment=1)	-0.27574900	0.61965400	-28.82642800
H(Fragment=1)	-1.41498000	-1.48921600	-29.60391900
H(Fragment=1)	-2.32005100	-0.75011600	-28.28411200
H(Fragment=1)	-0.17333000	-0.04346300	-27.16796100
C(Fragment=1)	-1.72101200	-2.35705400	-23.67897500
C(Fragment=1)	-3.00905500	-2.22674000	-24.48332900
C(Fragment=1)	-3.17868600	-0.83264900	-25.08392100
C(Fragment=1)	-4.38246100	-0.73719200	-26.01650700
N(Fragment=1)	-4.59899500	0.64929800	-26.44241600
C(Fragment=1)	-4.85596000	1.10462000	-27.67132700
N(Fragment=1)	-4.93027600	0.29642300	-28.72204200
N(Fragment=1)	-5.07067500	2.43087500	-27.83110200
H(Fragment=1)	-1.60401100	-3.38371200	-23.29642600
H(Fragment=1)	-0.85343800	-2.15152700	-24.32680700
H(Fragment=1)	-3.87697900	-2.46999600	-23.84783800
H(Fragment=1)	-2.98378300	-2.96661500	-25.29787800
H(Fragment=1)	-2.27416800	-0.57708600	-25.66077700
H(Fragment=1)	-1.69184700	-1.66043600	-22.82230100
H(Fragment=1)	-3.27552000	-0.08267700	-24.27357700
H(Fragment=1)	-5.29791600	-1.10147300	-25.52011000
H(Fragment=1)	-4.21806300	-1.37860300	-26.89554000
H(Fragment=1)	-4.60136000	1.33800000	-25.69739800
H(Fragment=1)	-5.05745200	0.69806300	-29.64323400
H(Fragment=1)	-4.71417300	-0.74227800	-28.71606800
H(Fragment=1)	-4.91866200	3.07821100	-27.06839400
H(Fragment=1)	-5.15718400	2.82763800	-28.75760000
C(Fragment=1)	-6.91306400	-2.49600500	-30.27706900
C(Fragment=1)	-6.08863600	-3.31448800	-29.28546300
S(Fragment=1)	-4.34667200	-2.72102800	-29.22706500
H(Fragment=1)	-6.94791500	-1.43328000	-29.98330300
H(Fragment=1)	-6.47857600	-2.55035100	-31.28881600
H(Fragment=1)	-6.08099700	-4.38043200	-29.55807800
H(Fragment=1)	-6.53307600	-3.25173800	-28.27828200
H(Fragment=1)	-7.95323000	-2.86424300	-30.32972800
Cu(Fragment=1)	-3.09558900	-4.19455200	-27.91644500
O(Fragment=2)	-5.02125000	-6.43741200	-28.64279100
O(Fragment=2)	-3.89323900	-5.86749300	-28.94899900

X 1 F
X 23 F
X 32 F
X 40 F
X 48 F
X 53 F
X 70 F
